# Ancient feeding-related neuropeptides regulate alloparenting in ants

**DOI:** 10.1038/s41586-026-10747-6

**Published:** 2026-07-08

**Authors:** Alexander Paul, Tomas Kay, Ivan Lacroix, Vikram Chandra, Asaf Gal, Patrick K. Piekarski, Stephany Valdés-Rodríguez, Amelia L. Ritger, Katelyn S. Lee, Kip D. Lacy, Daniel J. C. Kronauer

**Affiliations:** 1 Laboratory of Social Evolution and Behaviour, The Rockefeller University, New York, NY USA; 2 Howard Hughes Medical Institute, The Rockefeller University, New York, NY USA; 3 Present Address: Department of Organismic and Evolutionary Biology, Museum of Comparative Zoology, Harvard University, Cambridge, MA USA; 4 Present Address: Department of Ecology, Evolution, and Marine Biology, University of California, Santa Barbara, Santa Barbara, CA USA; 5 Present Address: Department of Mechanical Engineering, Columbia University, New York, NY USA; 6 Present Address: Zuckerman Mind Brain Behavior Institute, Columbia University, New York, NY USA

**Keywords:** Social behaviour, Social evolution

## Abstract

Alloparental care and division of labour are hallmarks of insect societies^1^. Social insect workers typically care for brood within the nest when they are young and transition to foraging outside the nest as they age^2–5^. This provides a powerful paradigm to study the neural basis of parenting and age-related behavioural change. Although previous work has interrogated aspects of these dynamics^6–14^, the underlying neural and molecular mechanisms remain poorly understood. Here, using an unbiased pharmacological screen of neuropeptides, we show that two ancestral regulators of feeding, neuropeptide F (NPF) and allatostatin A (AstA), modulate brood-care behaviour in the clonal raider ant. Through functional manipulations, we show that NPF increases brood-care behaviour, whereas AstA has the opposite effect. Furthermore, we find that the levels of NPF and AstA in the brain change naturally as ants age, suggesting that these changes underlie the age-related changes in brood-care behaviour. Finally, we show that, as in solitary species^15,16^, NPF and AstA remain sensitive to nutritional state, and nutritional state affects brood-care behaviour accordingly. Our results reveal that evolution has co-opted molecular mechanisms that regulated feeding ancestrally to enable cooperative brood care and age-associated division of labour.

## Abstract (web summary)

Pharmacological screening and behavioural assays in ants identify two specific neuropeptides that have key and opposite effects in regulating brood care, and that link nutritional state to age-dependent changes in parenting behaviour.

## Main

Animal societies are often organized around alloparental care, in which adults raise the offspring of others^17^. In social insect colonies, adults of different ages engage in distinct brood-care tasks^5^. Young workers typically care for larvae inside the nest, whereas older workers leave the nest to forage^1^. These notable and stereotyped age-associated changes in behaviour could provide fundamental insights into neural plasticity. However, how alloparental care has evolved and how age-associated division of labour is regulated at the molecular and neural level remain poorly understood.

**Fig. 1:**
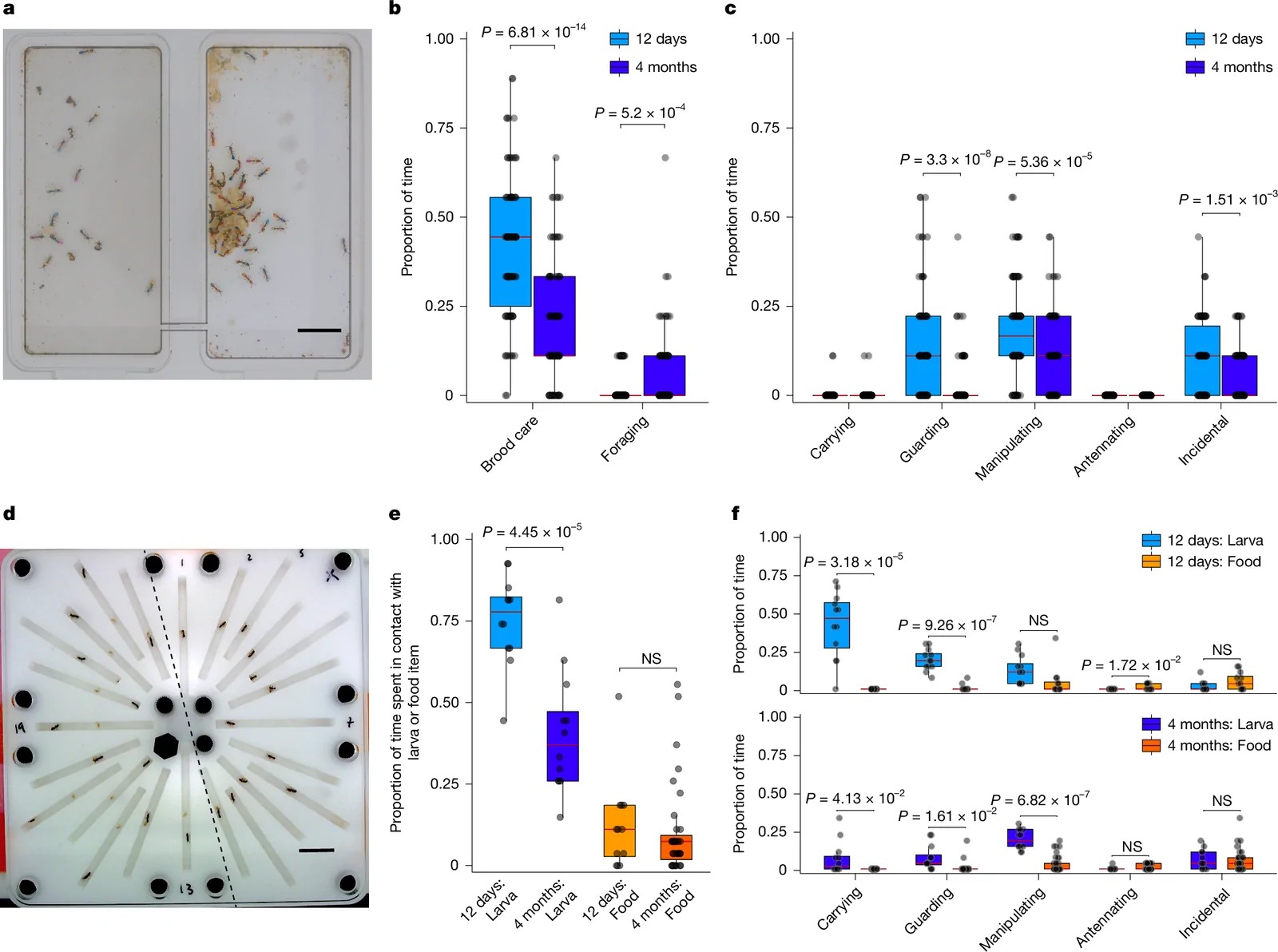
A high-throughput behavioural assay quantifies brood-care behaviour. **a**–**c**, The clonal raider ant (*O. biroi*) exhibits an age-based division of labour. **a**, Three colonies of 30 young (12 days old) and 30 old (4 months old) ants were tracked for 3 days in 2-chamber set-ups. Scale bar, 1 cm. **b**, On average, young ants spend approximately twice as much time in physical contact with larvae as do old ants (mean = 0.415 versus 0.194), whereas old ants spend about three times as much time foraging as do young ants (mean = 0.580 versus 0.160). **c**, Physical contact with the larvae is subcategorized as carrying, guarding (standing over a larva and being stationary), manipulating (antennal and mouthpart contact with the larva and active movement), antennating (antennal contact only, with antennae stretched forwards to contact the larva) or incidental (adjacent to, or oriented away from, the larva without obvious interaction). In **b**,**c**, *n* = 90 young and 90 old ants. **d**–**f**, These behavioural differences are recapitulated in a one-ant/one-larva assay. **d**, In the one-on-one assay, 24 ant–larva pairs are tracked simultaneously under a single camera. Young and old ants are in corridors 1–12 (right of the dashed line) and 13–24 (left of the dashed line), respectively. Whereas most larvae with old ants are visible, most young ants are occluding the larva while performing brood care. Scale bar, 1 cm. **e**, As in the colony assay, young ants spend approximately twice as much time in contact with larvae as do old ants (mean = 0.753 versus 0.404). By contrast, age had no effect on the proportion of time spent in physical contact with a food item (a fire ant (*Solenopsis invicta*) pupa). **f**, In the one-on-one assay, ant–larva interactions are qualitatively similar to those in the colony context (**c**), although larval carrying, a behaviour ants display when disturbed, increases substantially. By contrast, when ants are given a food item, the behaviours exhibited are qualitatively distinct; ants never carry and almost never guard food items. In **e**,**f**, *n* = 24 young and 47 old ants. Data in **b**,**c**,**e**,**f** were analysed with two-tailed Welch’s *t*-tests. NS, not significant. Boxes in **b**,**c**,**e**,**f** show the interquartile range (IQR), centre lines indicate medians and whiskers extend to 1.5× IQR. Source data

Accumulating evidence suggests that novel social behaviours frequently evolve through the co-option of ancient neuromodulators^6–11,13,14,18^. An emerging pattern is that the neuromodulators co-opted to regulate parenting-associated social behaviour have conserved roles in feeding, growth and reproduction, suggesting that involvement in these processes predisposes neuromodulators to regulate social behaviour^18–24^. We investigated this by studying the neuropeptidergic regulation of brood care in the clonal raider ant, *Ooceraea biroi*^25–27^. Within a colony, these ants are genetically identical^28,29^, providing a well-controlled experimental system in which to study the dynamics of age-dependent neural plasticity. Workers exhibit an ant-typical age-linked transition from brood care to foraging. Young ants (12 days old) spend on average over twice as much time nursing the brood as do old ants (4 months old), and less than half as much time foraging (Fig. 1a–c).

## Age-associated patterns of brood care

**Extended Data Fig. 1:**
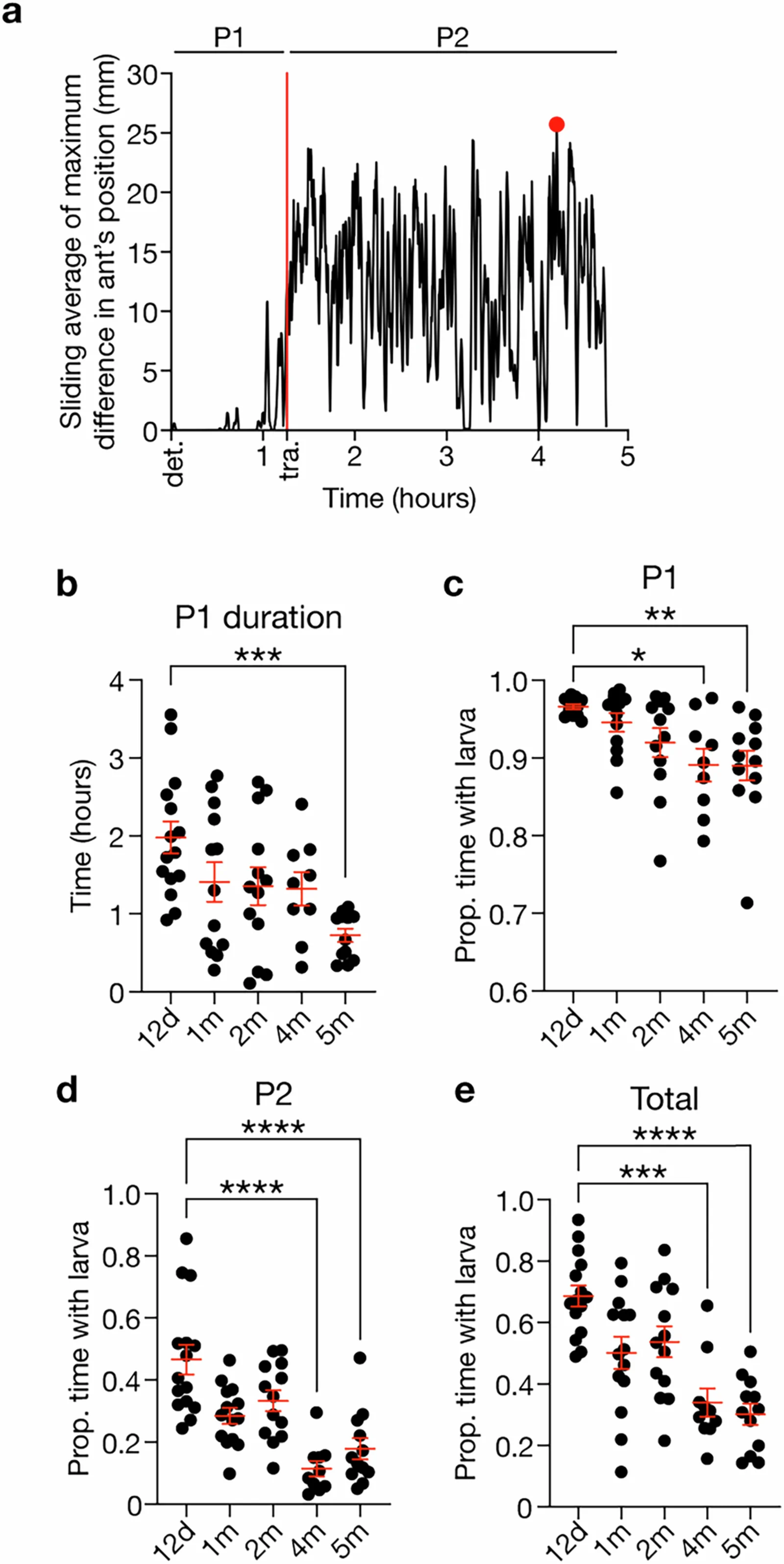
Definitions of P1 and P2, and varying brood-care responses with age. **a**, Example behavioural trace in the brood-care assay illustrating how the transition point between behavioural phases P1 and P2 was defined. The curve shows the sliding average of the maximum difference in the ant’s position per 80 s window over the brood-care assay. The transition point (red line) is defined as the moment in time when the curve first reaches half of its absolute maximum value (red dot). Details can be found in the Methods. **b**–**e**, Time course of brood-care responses to larvae in age-matched cohorts of 12-day-old, and 1-, 2-, 4-, and 5-month-old, ants. Quantifications are shown for time spent in P1 (**b**, n = 15 (12 d), 13 (1 m, 2 m), 9 (4 m) and 12 (5 m) ants), and the proportion of time ants physically interact with larvae in phases P1 (**c**, n = 12 (12 d, 1 m, 2 m, 5 m) and 10 (4 m) ants), P2 (**d**, total n = 15 (12 d), 14 (1 m), 13 (2 m), 10 (4 m), and 12 (5 m)), and over the total (**e**, n = 15 (12 d), 14 (1 m), 14 (2 m), 10 (54), and 12 (5 m) ants**)** assay. Data in **b**–**e** were analysed using Kruskal–Wallis ANOVAs (**b**: *H* = 14.95, **p = 0.0048, 12 d v 5 m p = 0.0011; **c**: *H* = 16.27, **p = 0.0027, 12 d v 4 m p = 0.0309, 12 d v 5 m p = 0.0075; **d**: *H* = 32.83, ****p = 1.29e-6, 12 d v 4 m p = 5.76e-6, 12 d v 5 m p = 2.51e-4; **e**: *H* = 29.03, ****p = 7.71e-6, 12 d v 4 m p = 3.44e-4, 12 d v 5 m p = 2.62e-50.0001) followed by Dunn’s multiple comparison tests, which are indicated in the figure panels. Bars represent mean ± s.e.m. Source data

To investigate the role of neuropeptides in alloparental care, we first developed a behavioural assay to measure the responses of young and old ants to larvae (Extended Data Fig. 1a). In this assay, individual ants are placed into a narrow corridor along with a single fourth instar larva and are filmed for 5 h (Fig. 1d, Extended Data Fig. 1a and Supplementary Video 1). Naturally, *O. biroi* colonies oscillate between reproductive phases, in which colonies comprise workers, eggs and pupae, and foraging phases, in which colonies comprise workers and larvae. The composition of developmental stages in our assay thus represents colonies in the foraging phase. In our assay, as in colonies, young ants spend twice as much time in contact with larvae as old ants do (Fig. 1e). Ants also exhibit the same nursing behaviours towards fourth instar larvae in this assay as they do in colonies (Fig. 1f). Of note, if presented with a food item instead of a larva, both young and old ants exhibit qualitatively different behaviours, and the age-linked behavioural differences disappear (Fig. 1f). Finally, ants never killed or consumed larvae in this assay. Together, this shows that our behavioural assay captures core aspects of alloparental behaviour. Although this assay is simple and high throughput, it has limitations. First, because no food source is provided, and because *O. biroi* adults do not feed larvae by trophallaxis, our assay does not capture brood-care behaviours associated with larval feeding. Second, the short duration precludes assessment of the functional consequences of brood care on larval growth and survival.

**Fig. 2:**
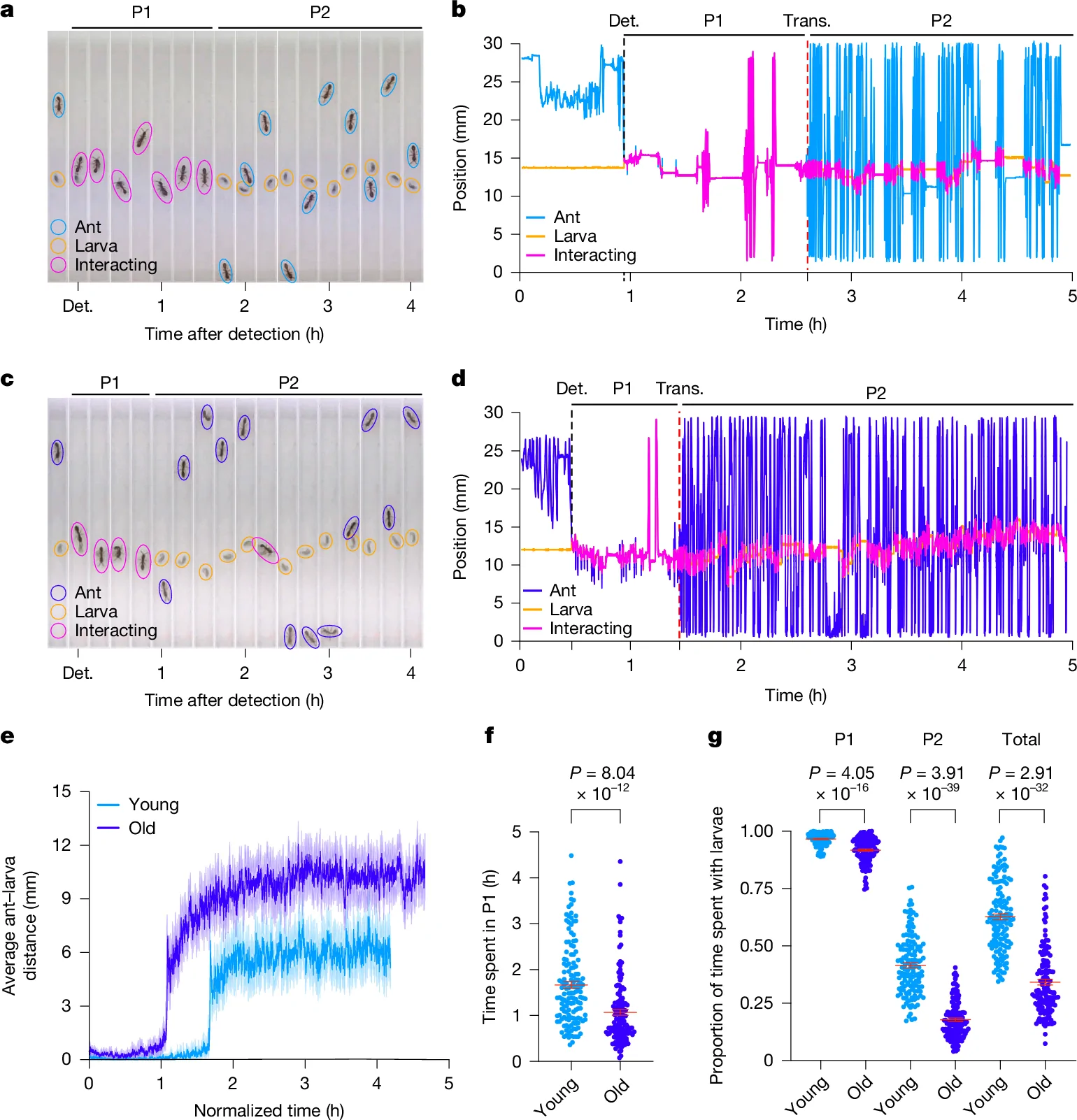
Automated behavioural analysis reveals biphasic and age-dependent responses to larvae. **a**–**d**, Representative chronograms and positional traces of a young (12 days old) (**a**,**b**) and an old (at least 2 months old) (**c**,**d**) ant interacting with a fourth instar larva in the brood-care assay. **a**,**c**, The first image shows the start of the assay. Images are then shown every 15 min from larva detection (Det.) onwards. **b**,**d**, Positional traces of the ant (blue), larva (orange) and physical interactions between ant and larva (magenta). Larva detection (Det.; black dashed line) and the transition point (Trans.; red dashed line) between the first (P1) and second (P2) behavioural phases are indicated. **e**, Average ant–larva distance in young (*n* = 143; light blue) and old (*n* = 143; dark blue) ants, with 95% confidence intervals in surround, aligned to larva detection at time 0. **f**, Quantification of the time ants spend in the P1 phase (*n* = 143 ants per age group). **g**, Quantification of the proportion of time ants physically interact with larvae in phases P1 and P2, and over the total assay. P1: *n* = 137 young and *n* = 133 old ants. P2 and total: *n* = 143 young and *n* = 142 old ants. Data in **f**,**g** were analysed with one-sided Mann–Whitney tests (**g**, *U* = 5,512; **f**, P1, *U* = 3,945; P2, *U* = 1,085; total, *U* = 1,967). Mean ± s.e.m. Source data

**Extended Data Fig. 2:**
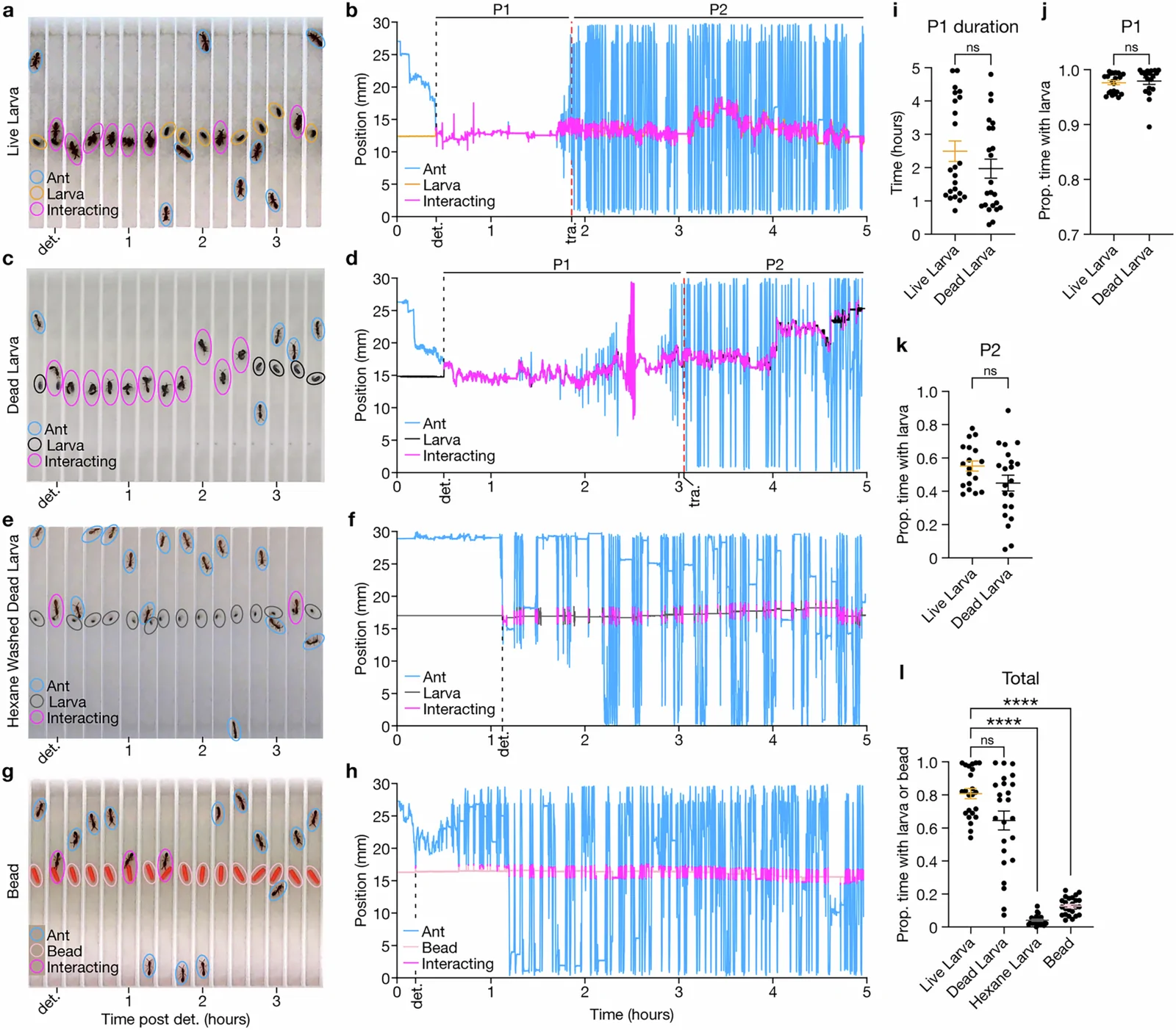
Larval chemical cues are required for ant brood-care responses. **a**–**h**, Representative chronograms and positional traces of young ants interacting with a live larva (**a**,**b**), a dead larva (**c**,**d**), a dead larva washed in hexane to remove cuticular hydrocarbons (CHCs) and possibly other chemical cues (**e**,**f**), or a plastic bead (**g**,**h**) in the brood-care assay. **a**,**c**,**e**,**g**, First image shows the start of the assay, then starting at larva or bead detection (det.) images are shown every 15 min. **b**,**d**, Positional trace of the ant (blue), live larva (orange) (**b**), dead larva (black) (**d**), and physical interactions between ant and larva (magenta). Larva detection (det.; black dashed line) and transition point (tra.; red dashed line) between the first (P1) and second (P2) behavioural phases are indicated. **f**,**h**, Positional trace of the ant (blue), hexane-washed dead larva (grey) (**f**), bead (pink) (**h**), and physical interactions between ant and larva or bead (magenta). Larva or bead detection (det.; black dashed line) is indicated. However, no distinct P1 and P2 phases were observed in these interactions. **i**–**k**, Quantification of time spent in P1 (**i**, n = 23 ants per condition) and the proportion of time ants physically interact with larvae in phases P1 (**j**, n = 23 (live larva) and 19 (dead larva) ants), and P2 (**k**, n = 18 (live larva) and 21 (dead larva) ants). **l**, Quantification of the total proportion of time ants physically interact with larvae or beads (total n = 23 (live larva), 24 (dead larva, bead), and 21 (hexane larva) ants). Data in **i**–**k** were analysed using one-sided Mann-Whitney tests (**i**: *U* = 193; **j**: *U* = 163; **k**: *U* = 132). Data in **l** were analysed using a Kruskal–Wallis ANOVA (*H* = 71.37, ****p = 2.17e-15, live larva v hexane-washed dead larva p = 5.55e-13, live larva v plastic bead p = 1.6e-6) followed by Dunn’s multiple comparison tests, which are indicated in the figure panel. Bars represent mean ± s.e.m. Source data

Ants exhibit a stereotypical two-phase response to larvae. In the first phase (P1), ants spend most of their time in direct physical contact with the larva. In the second phase (P2), ants tend to leave the larva for short bouts as they move around the corridor (Fig. 2a–e and Extended Data Fig. 1b). This two-phase response is specific towards larvae and depends on hexane-soluble larval compounds^30^ (Extended Data Fig. 2). Young ants spend significantly more time in P1 than do old ants (Fig. 2e,f). The age-linked difference in time spent with larvae is evident within each phase as well as across the total assay period (Fig. 2g). Time-course analysis of age-matched cohorts of ants in this assay reveals a gradual decrease in time spent with larvae with age (Extended Data Fig. 1c–f).

## NPF and AstA regulate brood-care behaviour

**Extended Data Fig. 3:**
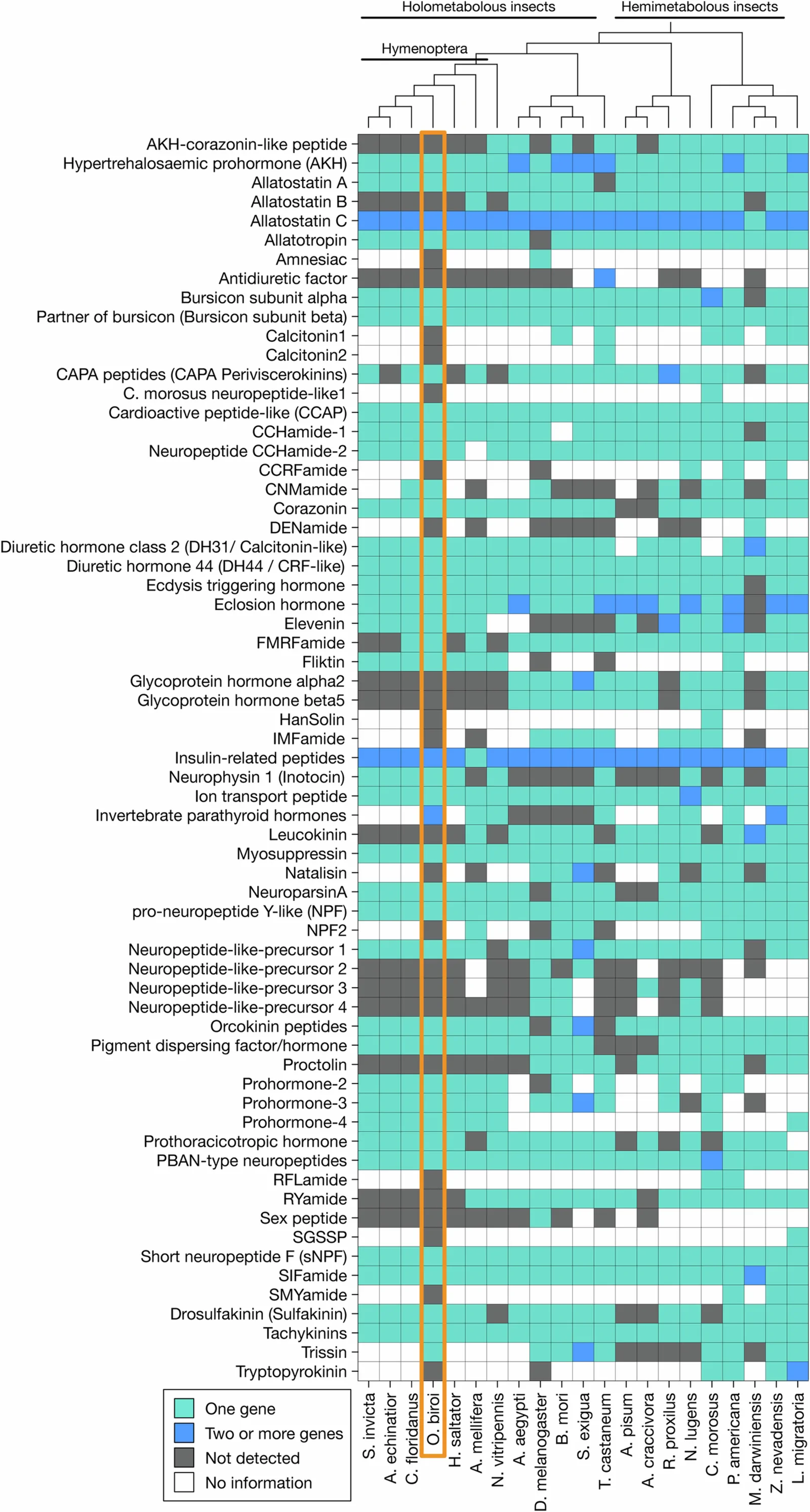
The clonal raider ant neuropeptidome is similar to those of other Hymenoptera. Colour map indicating presence and absence of neuropeptide precursor genes across insects. Light blue indicates one precursor gene present, dark blue indicates two or more precursor genes present, grey indicates cases in which precursor genes were searched for but not found, and white indicates in which no information was given in the neuropeptidome publication(s) for the respective species. Neuropeptide precursor genes in *O. biroi* were identified by sequence homology to known neuropeptide genes of the insect species shown in the colour map, as well as to those of additional species across the animal kingdom. For clarity and brevity, some of the shown gene names are common names. Additional details are provided in the Methods and Supplementary Table 1. We identified 42 neuropeptide precursor genes in *O. biroi*. The *O. biroi* neuropeptidome is overall similar to those of other Hymenoptera, showing similar conservation, loss, and duplications of neuropeptide precursor genes. Abbreviations: *S. invicta* (*Solenopsis invicta*), *A. echinatior* (*Acromyrmex echinatior*), *C. floridanus* (*Camponotus floridanus*), *O. biroi* (*Ooceraea biroi*), *H. saltator* (*Harpegnathos saltator*), *A. mellifera* (*Apis mellifera*), *N. vitripennis* (*Nasonia vitripennis*), *A. aegypti* (*Aedes aegypti*), *D. melanogaster* (*Drosophila melanogaster*), *B. mori* (*Bombyx mori*), *S. exigua* (*Spodoptera exigua*), *T. castaneum* (*Tribolium castaneum*), *A. pisum* (*Acyrthosiphon pisum*), *A. craccivora* (*Aphis craccivora*), *R. prolixus* (*Rhodnius prolixus*), *N. lugens* (*Nilaparvata lugens*), *C. morosus* (*Carausius morosus*), *P. americana* (*Periplaneta americana*), *M. darwiniensis* (*Mastotermes darwiniensis*), *Z. nevadensis* (*Zootermopsis nevadensis*), *L. migratoria* (*Locusta migratoria*).

**Extended Data Fig. 4:**
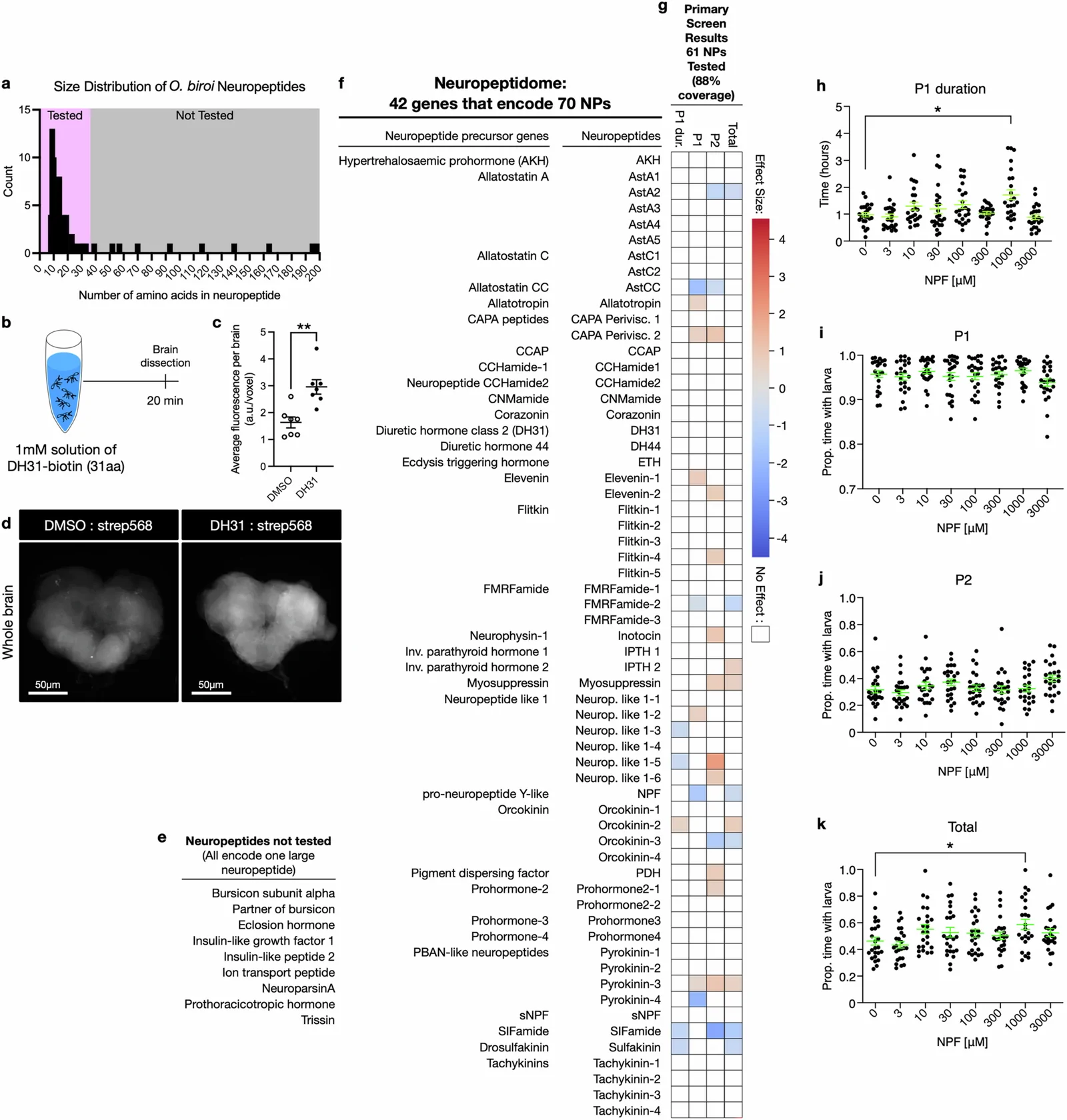
Pharmacological screens. **a**, Histogram showing the size distribution of *O. biroi* neuropeptides by the number of amino acids in each neuropeptide. Pink shading indicates those neuropeptides that were tested in the primary screen, and grey shading indicates those neuropeptides that could not be synthesized and therefore were not tested. **b**–**d**, Control experiment verifying that neuropeptides across the size range of those tested here do enter the brain in our pharmacological treatment (see Methods for details). **b**, Schema of experiment. Live ants were soaked in a 1 mM solution of biotinylated-DH31, a 31-amino-acid neuropeptide, for 20 min. Brains were then dissected and streptavidin-fluorophore stained to measure DH31 incorporation. **c**, Quantification of average fluorescence per brain of ants soaked in biotinylated-DH31 or DMSO control. n = 7 brains per condition, **p = 0.0012. **d**, Representative confocal z-stack projections of whole brains from ants soaked in biotinylated-DH31 or DMSO control. **e**,**f**, The *O. biroi* neuropeptidome contains 42 precursor genes that are post-translationally cleaved into 70 unique neuropeptides. Nine precursor genes each encode one large neuropeptide with complex secondary structure. These could not be synthesized and thus were not tested (**e**).** g**, Heat map showing the primary screen results by effect size (Cohen’s d) on brood-care behaviour. Significant effects (p < 0.05 in Mann-Whitney U tests compared to control, see Supplementary Table 4 for details) are indicated by colour, and white indicates no effect. Reds and blues indicate more or less proportion of time spent interacting with larvae in P1, P2, and total, or longer or shorter P1 duration, respectively. n = 12 ants per neuropeptide were tested. **h**–**k**, Additional dose–response of NPF. Graphs show quantification of P1 duration (**h**, n = 21 ants per condition) and the proportion of time in physical contact with larvae in P1 (**i**, n = 24 ants per condition), P2 (**j**, n = 23 (10 µM, 1,000 µM) and 24 ants for all other conditions), and total (**k**, n = 24 ants per condition) of 12-day-old ants treated with increasing doses of NPF. Data in **c** were analysed using a one-sided Mann-Whitney test (*U* = 2). Data in **h**,**i**,**j**,**k** were analysed by ordinary ANOVA (**h**: *F*_*(7,184)*_ = 3.59, **p = 0.00122, *0 µM v 1,000 µM p = 0.00415; **i**: ns, **j**: ns, **k**: *F*_*(7,184)*_ = 2.39, p = 0.0230, *0 µM v 1,000 µM p = 0.0293) followed by Dunnett’s multiple comparison tests, which are indicated in the figure panel. Bars in **h**,**i**,**j**,**k** represent mean ± s.e.m. Source data

Next, we annotated the *O. biroi* neuropeptidome to build a pharmacological screening library. We identified 42 neuropeptide precursor genes on the basis of sequence homology to other animals (Supplementary Table 1), which is consistent with previously described hymenopteran neuropeptidomes^31–37^ (Extended Data Fig. 3). Neuropeptide precursor proteins are post-translationally cleaved into one or more neuropeptides that then undergo various modifications as they are transported to axon terminals^38^. Neuropeptide sequences show high levels of amino acid conservation across species and are flanked by distinct proteolytic cleavage site motifs^39^. On the basis of these features, we identified 70 unique neuropeptides encoded by the 42 precursor genes (Supplementary Tables 2 and 3 and Extended Data Fig. 4).

For our screening library, we synthesized 61 of the 70 neuropeptides, ranging from 7 to 35 amino acids in length (Extended Data Fig. 4a). The remaining 9 neuropeptides are large (more than 35 amino acids), structurally complex and challenging to synthesize. They were thus excluded (Supplementary Table 3 and Extended Data Fig. 4e).

In a primary screen, we treated ants by soaking them in 1-mM solutions of neuropeptides. This method was previously used in *O. biroi*^6^, and we also validated its effectiveness for neuropeptides at the upper size limit of those in our screening library (Extended Data Fig. 4b–d). In this screen, we tested all 61 neuropeptides in the behavioural assay (Extended Data Fig. 4f). We found 24 candidate neuropeptides that modulated the time that ants spent with larvae (Supplementary Table 4 and Extended Data Fig. 4f). Given that this initial screen was designed for high throughput and our statistical approach was permissive, we expected many false positives among our candidates (see Methods for details on and limitations of this screen). We therefore ranked the hits from the primary screen by their *P* value and effect size (Cohen’s *d*; see Methods), and selected the top half of the candidate neuropeptides (*n* = 12) for more rigorous testing in a secondary round of screening using high-quality peptide solutions and dose–response analyses (Supplementary Table 4).

**Fig. 3:**
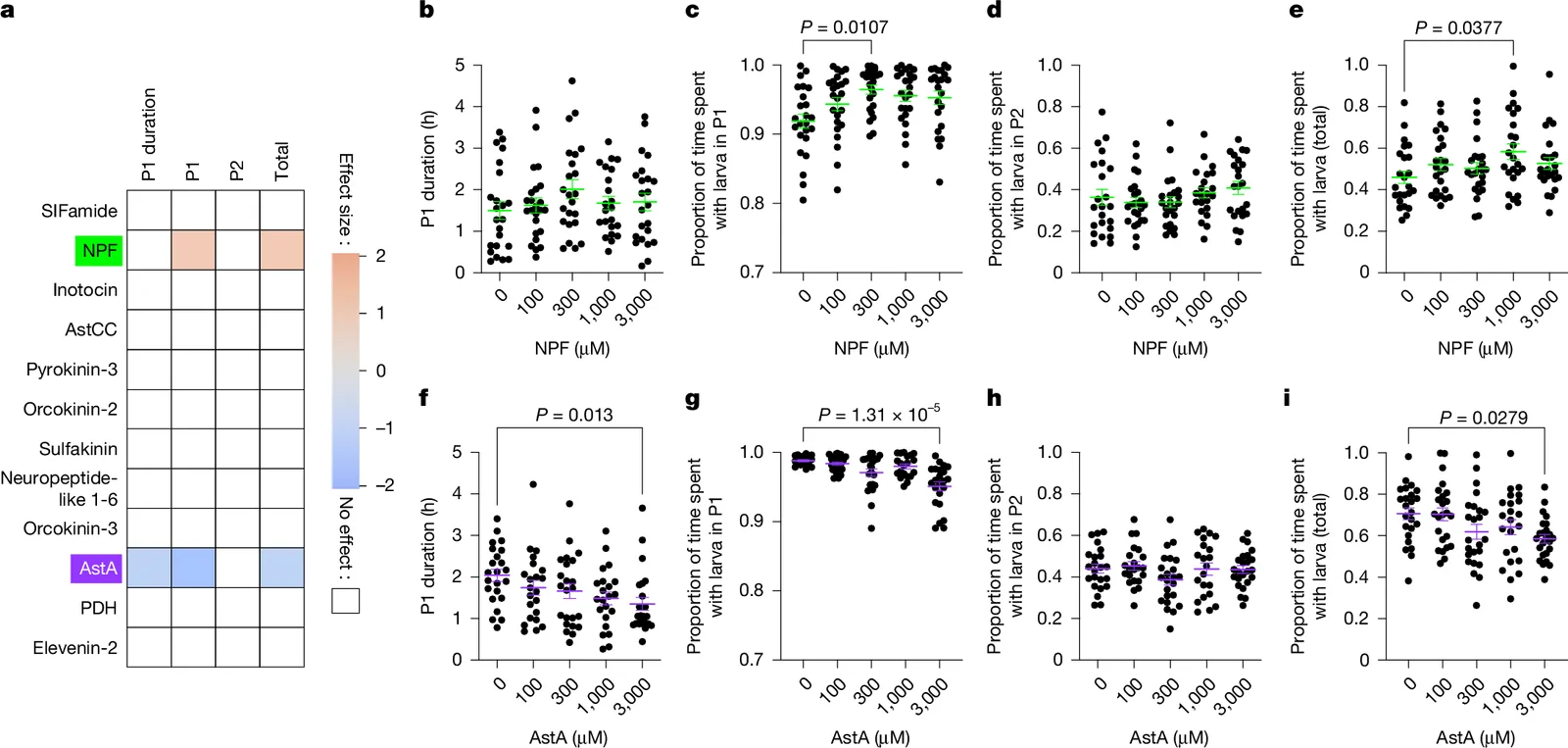
An unbiased neuropeptide screen identifies NPF and AstA as regulators of brood-care behaviour. **a**, Heat map showing the neuropeptides tested in the secondary screen, and the results by effect size (Cohen’s *d*) on brood-care behaviour. The two peptides that had significant effects are highlighted in green (NPF) and purple (AstA). Reds and blues indicate longer or shorter P1 duration, or more or less time spent with larvae in different phases of the assay (P1, P2 and total), relative to control ants. *n* = 24 young ants paired with 1 larva each were treated with peptide solutions of 0, 100, 300, 1,000 and 3,000 μM. **b**–**i**, Quantification of P1 duration (**b**,**f**) and the proportion of time in physical contact with larvae in P1 (**c**,**g**), P2 (**d**,**h**) and the total assay (**e**,**i**), in ants treated with increasing doses of NPF (**b**–**e**; *n* = 22 ants for 1,000 μM in **b**,**d**; 23 ants for 0 μM, 300 μM and 3,000 μM in **b**,**d** and 3,000 μM in **c**; and 24 ants for all other conditions) or AstA (**f**–**i**; *n* = 20 ants for 100 μM in **g**; 21 ants for 0 μM in **g** and 1,000 μM in **h**; 22 ants for 100 μM in **f**,**h** and 1,000 μM in **f**; 23 ants for 0 μM and 300 μM in **f**,**h** and 1,000 μM in **i**; and 24 ants for all other conditions) peptides. Data in **b**,**c**,**f**,**g** were analysed with Kruskal–Wallis ANOVAs (**b**, NS; **c**, *H* = 12.93, *P* = 0.0116; **f**, *H* = 11.34, *P* = 0.0230; **g**, *H* = 27.89, *P* = 1.32 × 10^−5^) followed by Dunn’s multiple comparison tests. *P* values for significant pairwise comparisons are provided in the figure. Data in **d**,**e**,**h**,**i** were normalized by square root transformation and analysed by ordinary ANOVAs (**d**,**e**,**h**: NS; **i**: *F*_(4,114)_ = 2.911, *P* = 0.0246) followed by Dunnett’s multiple comparison tests. Statistically significant results of multiple comparison tests are indicated in the figure panels. Mean ± s.e.m. Source data

Out of the 12 tested candidate neuropeptides, the secondary screen recovered 2 hits: NPF (encoded by the precursor gene pro-neuropeptide-y-like (LOC113562173)) and AstA (the precursor gene allatostatin A (LOC105283611) encodes five AstA peptides, but the effect was specific to AstA2) (Fig. 3a). These neuropeptides are evolutionarily conserved, with NPF being homologous to vertebrate NPY and the AstA receptor being homologous to the vertebrate galanin receptor^40,41^. NPF increased the amount of time that ants spent with larvae in the secondary screen (Fig. 3b–e). However, because this result was inconsistent with the primary screen (Extended Data Fig. 4g), we repeated the dose–response experiment. As expected, given the caveats associated with the primary screen, the extra dose–response experiment confirmed the results from the secondary screen and implied that the effect observed in the primary screen was due to chance (Extended Data Fig. 4h–k). AstA, on the other hand, decreased the ants’ affinity to larvae (Fig. 3f–i). This suggests that NPF promotes a nurse-like behavioural state typical of young ants, whereas AstA promotes a forager-like behavioural state characteristic of old ants. Although neuropeptides can have highly pleiotropic effects, NPF and AstA are arguably best known as ancestral regulators of feeding^15,42^. Our results raise the possibility that, during the evolutionary origin of parental (and/or alloparental) behaviour in ants, these two neuropeptides were co-opted to regulate brood-care behaviour and the age-associated reduction in brood care.

## Levels of NPF and AstA change with age

**Extended Data Fig. 5:**
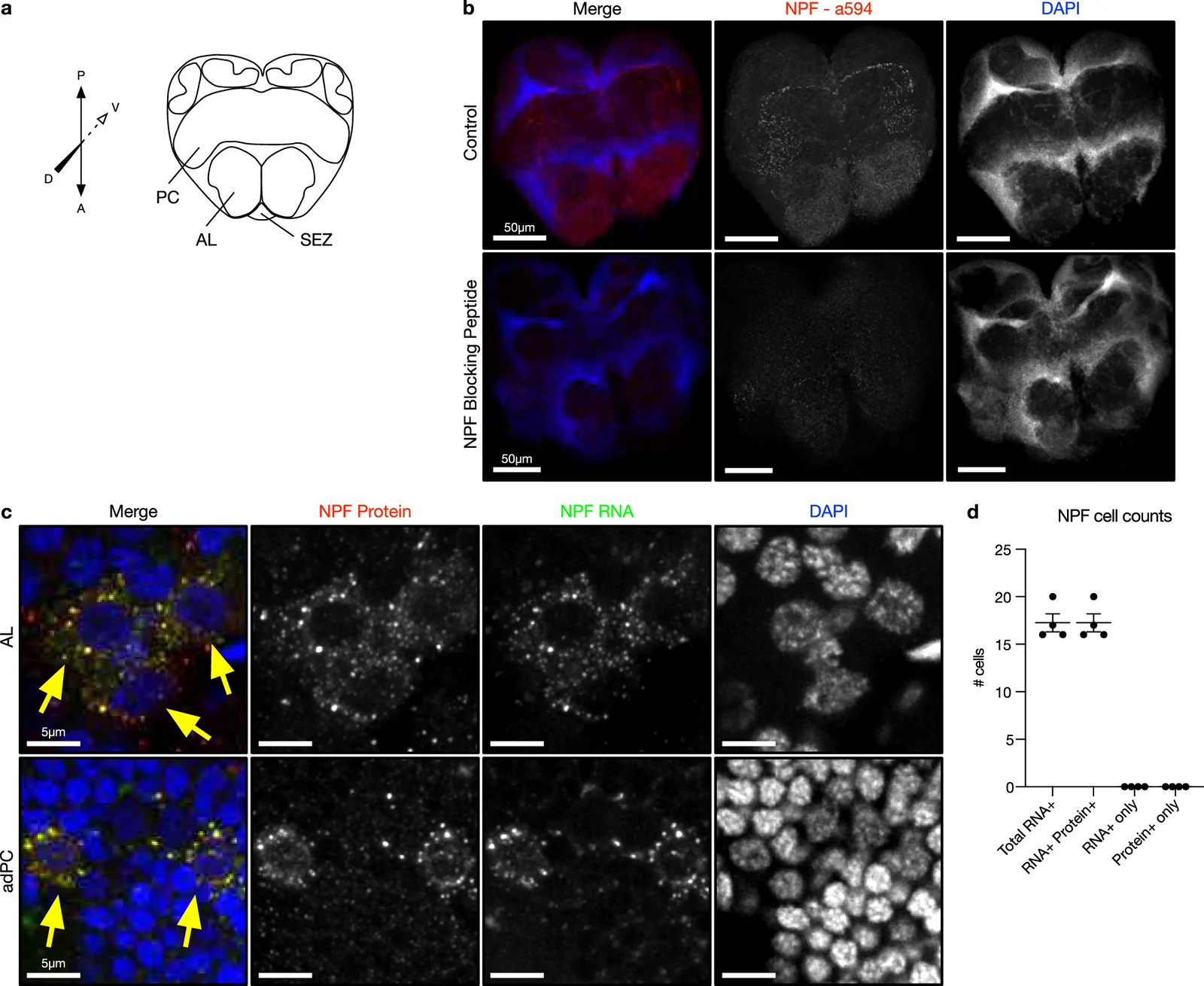
The NPF antibody specifically labels NPF-expressing cells. **a**, Schema of frontal view of ant brain indicating the protocerebrum (PC), antennal lobe (AL), and suboesophageal zone (SEZ). Axes indicate orientation (A: anterior; P: posterior; D: dorsal; V: ventral). **b**, Representative, frontal view confocal z-projection images of whole brains stained with control NPF antibody (top row) and NPF antibody preabsorbed with NPF blocking peptide (bottom row). Preabsorbing the NPF antibody with NPF peptide abrogates staining. NPF antibody staining in red, DAPI (DNA) staining in blue. **c**, Representative confocal z-projection images of brains co-stained with RNA FISH probes and antibody against NPF to simultaneously label NPF RNA and protein. Top row shows cells in the antennal lobe (AL) and bottom row shows cells in the adPC. The NPF antibody only labels NPF RNA+ cells (yellow arrows). **d**, Quantification of the number of cells stained per brain for both NPF RNA and protein. n = 4 brains analysed; bars represent mean ± s.e.m. Source data

**Extended Data Fig. 6:**
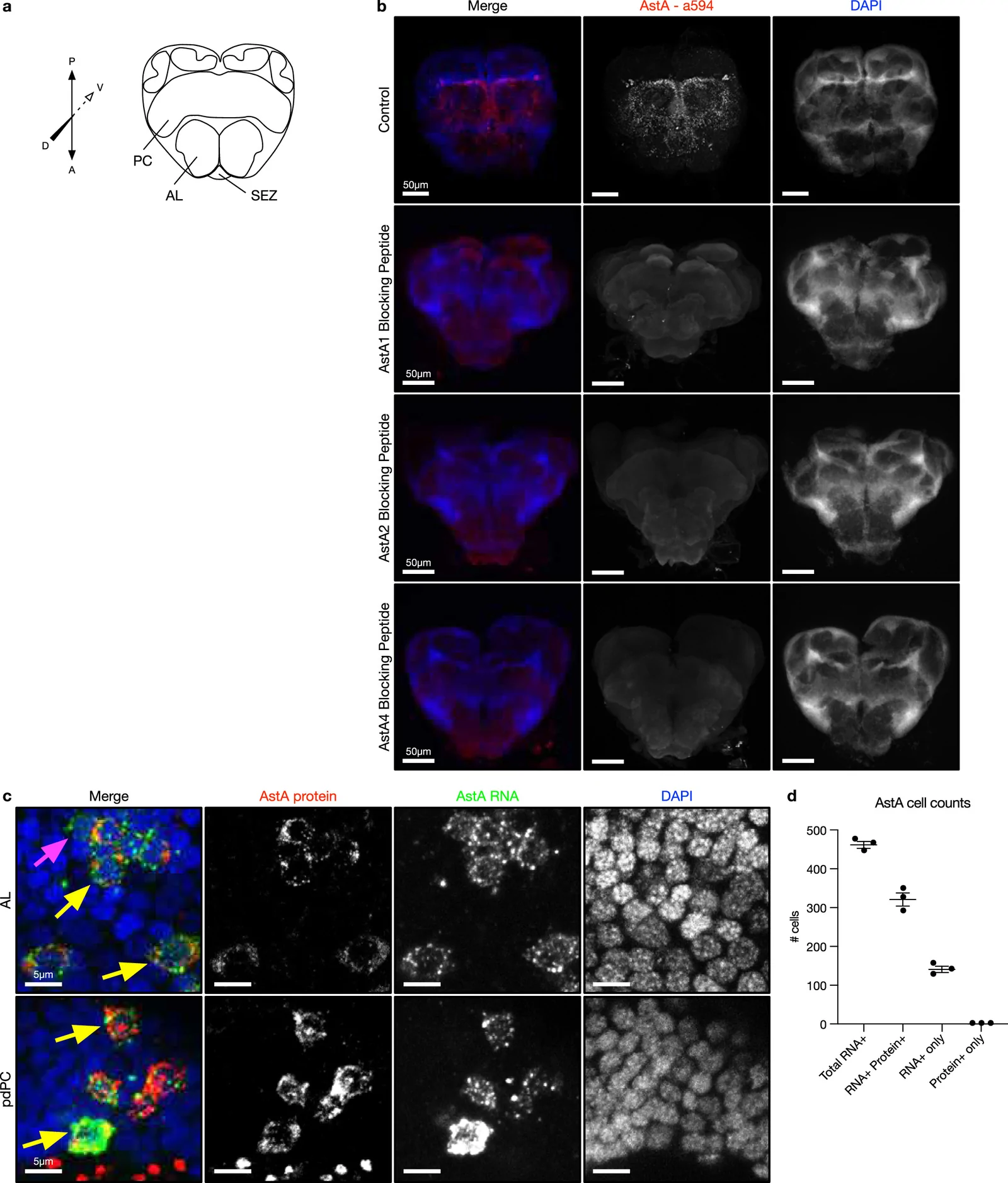
The AstA antibody specifically labels AstA-expressing cells. **a**, Schema of frontal view of ant brain indicating the protocerebrum (PC), antennal lobe (AL), and suboesophageal zone (SEZ). Axes indicate orientation (A: anterior; P: posterior; D: dorsal; V: ventral). **b**, Representative frontal view confocal z-projection images of whole brains stained with control AstA antibody (top row) and AstA antibody preabsorbed with AstA1, AstA2, or AstA4 blocking peptides (2nd, 3rd and 4th row, respectively). Preabsorbing the AstA antibody with AstA peptides abrogates staining. AstA antibody staining in red, DAPI (DNA) staining in blue. **c**, Representative confocal z-projection images of brains co-stained with RNA FISH probes and antibody against AstA to label AstA RNA and protein simultaneously. Top row shows cells in the antennal lobe (AL) and bottom row shows cells in the pdPC. The AstA antibody only labels AstA RNA+ cells (yellow arrows), while some AstA RNA+ cells are not labelled by the AstA antibody (magenta arrow). It is possible that the AstA ‘RNA+ only’ cells were not translating AstA protein at the time of brain dissection, or that these cells only store AstA5, a neuropeptide derived from the same gene but that our AstA antibody does not bind (see Methods). **d**, Quantification of the number of cells stained per brain for both AstA RNA and protein. n = 3 brains analysed; bars represent mean ± s.e.m. Source data

**Extended Data Fig. 7:**
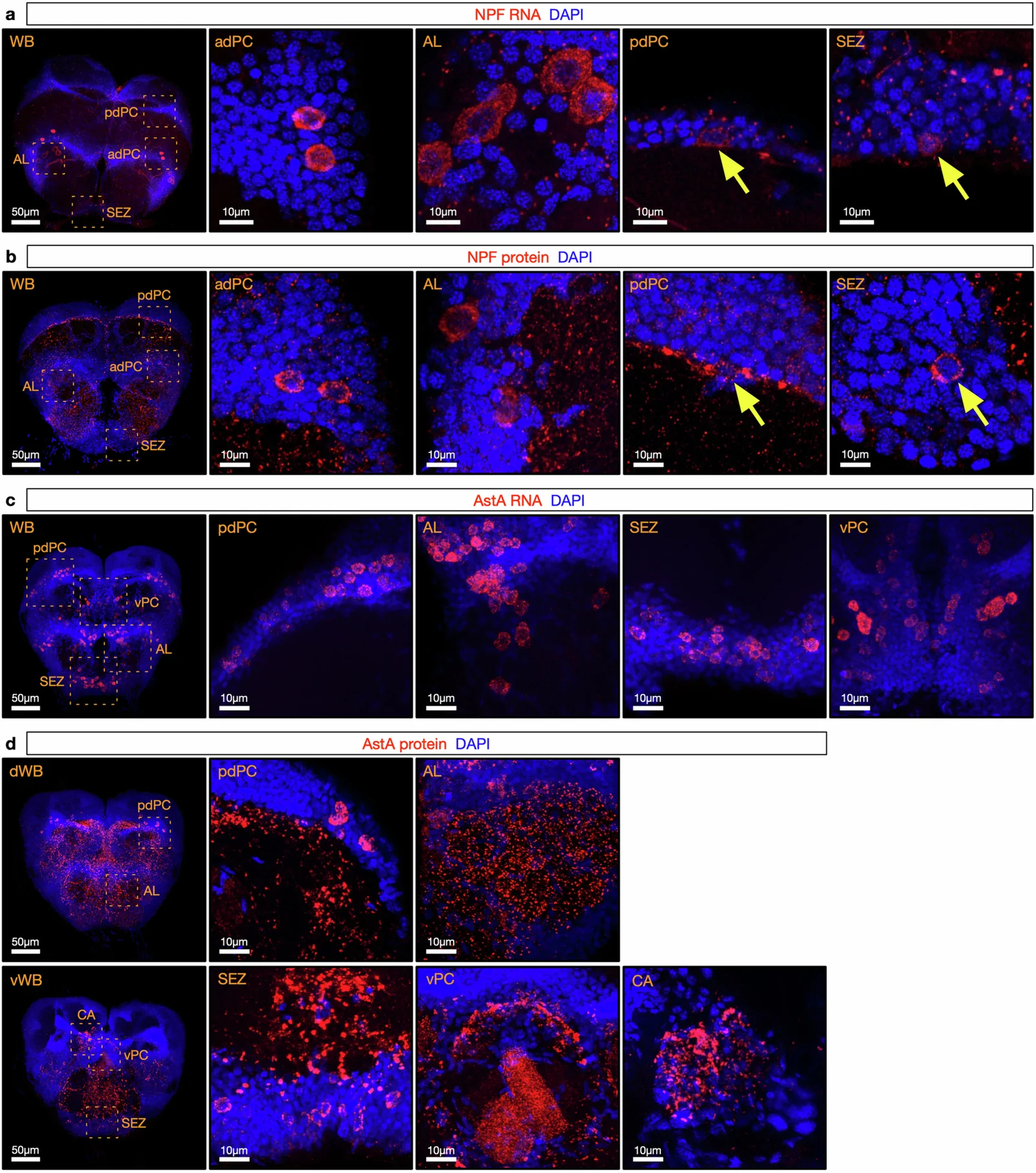
NPF and AstA RNA and protein localization patterns in the ant brain. **a**–**d**, Representative frontal view confocal z-projection images of ant brains stained in red for *NPF* RNA (**a**), NPF protein (**b**), *AstA* RNA (**c**), and AstA protein (**d**) with nuclear counterstain DAPI in blue. Images on the left show whole brains (WB), with orange boxes indicating regions shown in zoomed images to the right. **a**, Four bilaterally symmetric *NPF* RNA+ cells are located adjacent to the adPC. Eight cells are adjacent to the antennal lobes (AL), two cells are adjacent to the pdPC, and two cells are adjacent to the SEZ. *NPF* RNA staining is dim in the somas of SEZ and pdPC cells (yellow arrows). **b**, The NPF antibody labels cells adjacent to the adPC, AL, pdPC, and SEZ. NPF protein is located in neuropil in the dorso-lateral protocerebrum and the antennal lobes. NPF protein staining is dim in the somas of SEZ and pdPC cells (yellow arrows). **c**, *AstA* RNA+ cells are located adjacent to the pdPC, the AL, the SEZ, and the ventral protocerebrum (vPC). **d**, The AstA antibody labels cells adjacent to the pdPC, AL, SEZ, and vPC as well as neuropil throughout these regions. AstA protein is also located in the corpora allata (CA). However, no *AstA* RNA is found in the CA (not shown), suggesting that the AstA protein staining is a result of innervation from AstA-expressing neurons elsewhere in the brain as seen in other insects (see ref. ^59^). The brains displayed in this figure are representative of the >90 brains imaged for this study.

To investigate this further, we localized and measured the RNA and protein levels of NPF and AstA in ant brains (Extended Data Figs. 5–7). RNA fluorescent in-situ hybridization (FISH) staining revealed that *NPF* is expressed in 16–20 cells, whereas *AstA* is expressed in approximately 500 cells (Extended Data Figs. 5d, 6d and 7a,c and Supplementary Video 2). Immunohistochemistry (IHC) staining for NPF revealed that this peptide is localized to small clusters of cells in the protocerebrum (six cells), antennal lobe (eight cells) and suboesophageal zone (SEZ; two cells) (Extended Data Fig. 7b and Supplementary Video 3). IHC staining showed that AstA peptide is broadly present throughout the brain in the protocerebrum, antennal lobe, SEZ and corpora allata, a neuroendocrine accessory organ (Extended Data Fig. 7d and Supplementary Video 3). NPF and AstA peptides show anatomical overlap in the dorso-lateral protocerebrum and antennal lobe, suggesting that these neuropeptides might modulate circuitry in these brain regions to exert their effects on brood-care behaviour (Extended Data Fig. 7 and Supplementary Video 3).

**Fig. 4:**
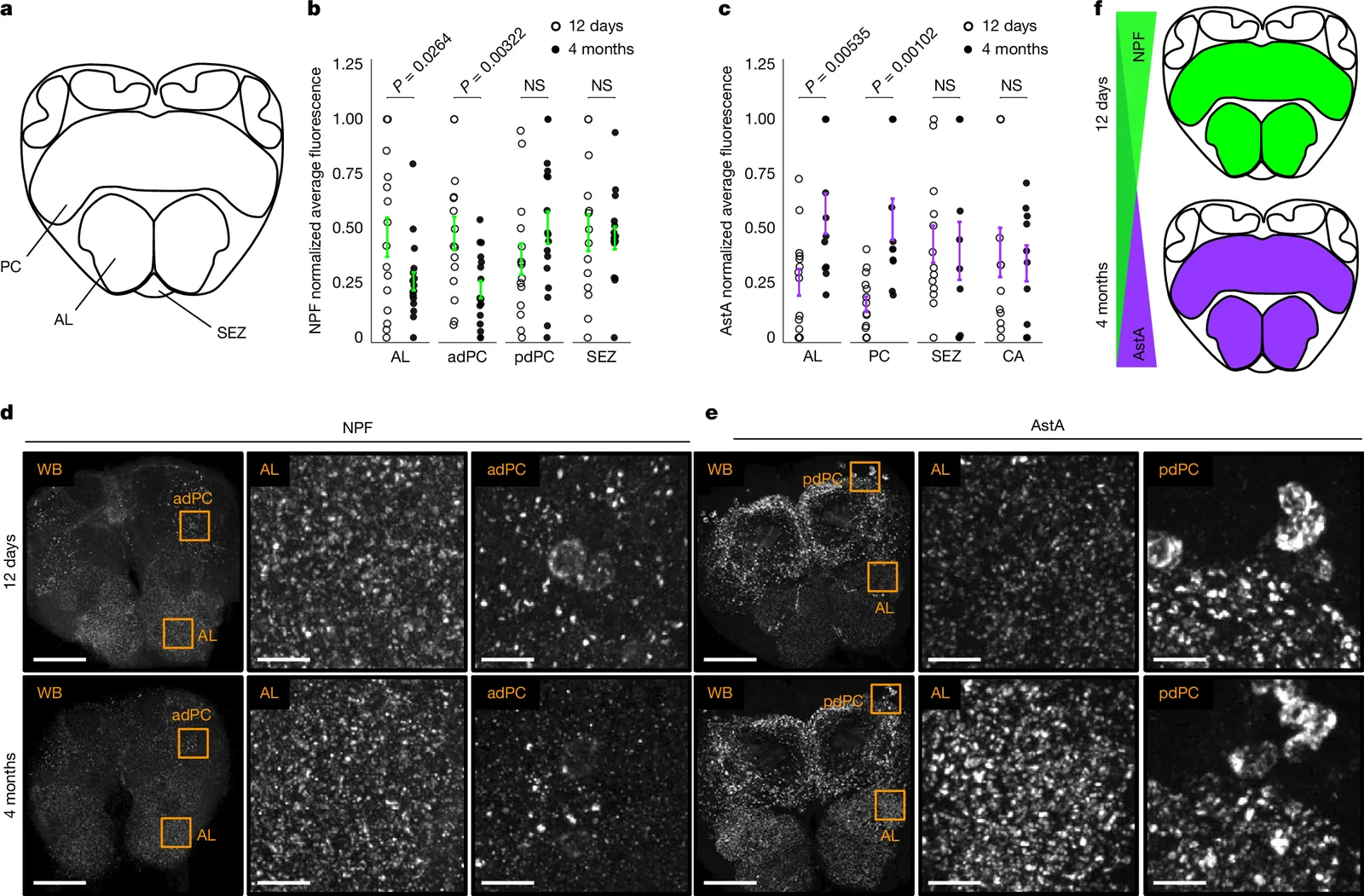
Levels of NPF and AstA peptides in the brain change with age. **a**, Schema of frontal view of the ant brain with the protocerebrum (PC), antennal lobe (AL) and SEZ neuropils indicated. **b**,**c**, Quantification of normalized average peptide levels of NPF (**b**; AL, *n* = 15 (12 days (d)) and 18 (4 months (m)); adPC, *n* = 15 (12 d) and 17 (4 m); pdPC, *n* = 15 (12 d) and 17 (4 m); SEZ, *n* = 14 (12 d) and 16 (4 m)) and AstA (**c**; AL, *n* = 13 (12 d) and 11 (4 m); PC, *n* = 13 (12 d) and 11 (4 m); SEZ, *n* = 13 (12 d) and 9 (4 m); corpora allata (CA), *n* = 11 (12 d) and 11 (4 m)) through antibody staining in the brains of young (12 days old) and old (4 months old) ants. Data are shown separately for the AL, adPC, pdPC and SEZ for NPF, and for the AL, PC, SEZ and CA for AstA. **d**,**e**, Representative frontal view confocal *z*-stack projections of brains from 12-day-old and 4-month-old ants stained for NPY (**d**) and AstA (**e**) peptides. The left images show the whole brain (WB); orange boxes indicate areas of adPC (**d**) or pdPC (**e**) and AL that are shown in magnified images on the right. Scale bars, 50 μm (left images) and 5 μm (right (magnified) images). **f**, Schema of changes in the levels of NPF and AstA peptides in ageing ant brains. NPF levels in the PC and AL decrease with age, whereas AstA levels in the same neuropils increase. Data in **b**,**c** were analysed with one-sided Welch’s *t*-tests (**b**, AL, *t* = 2.06, degrees of freedom (df) = 19.9, *P* = 0.0264; adPC, *t* = 3.02, df = 21.5, *P* = 0.00322; **c**, AL, *t* = −2.85, df = 18.0, *P* = 0.00535; PC, *t* = −3.86, df = 13.7, *P* = 0.00102). Mean ± s.e.m. Source data

**Extended Data Fig. 8:**
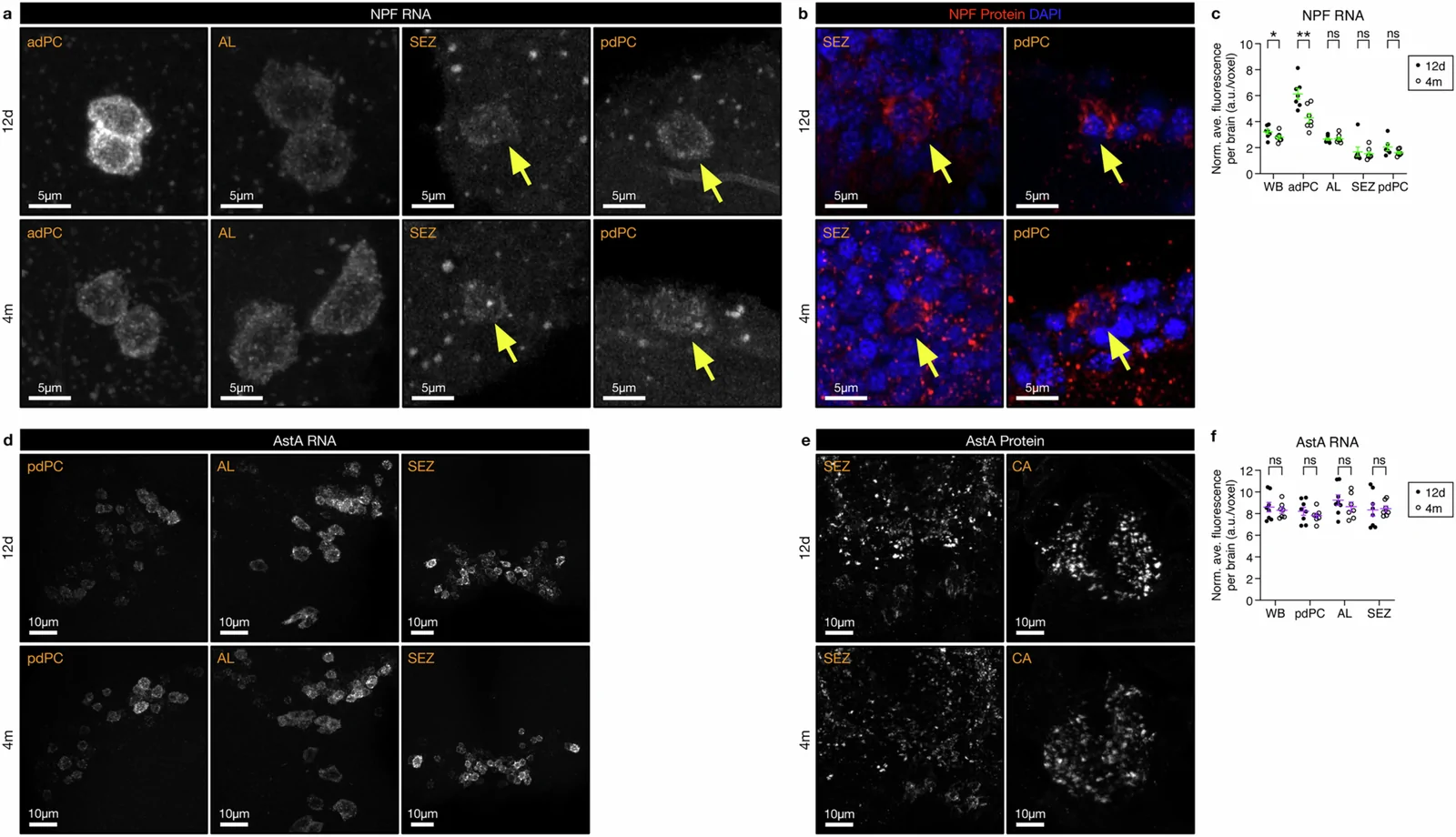
Additional changes in NPF and AstA RNA expression and protein levels with age. **a**, Representative confocal z-projection images of *NPF* RNA-expressing cells in the adPC, AL, SEZ and pdPC of 12 d old and 4 m old ants. *NPF* RNA staining is dim in SEZ and pdPC cells (yellow arrows). **b**, Representative confocal z-projection images of NPF protein in cells in the SEZ and pdPC of 12 d old and 4 m old ants. Red shows NPF protein and blue shows the nuclear counterstain DAPI. NPF protein staining is dim in SEZ and pdPC cells (yellow arrows). **c**, Quantification of normalized average *NPF* RNA expression in the whole brain (WB), adPC, AL, SEZ and pdPC of 12 d old and 4 m old ants (n = 6 (pdPC, 12 d) or 7 (all other conditions)). **d**, Representative confocal z-projection images of *AstA* RNA-expressing cells in the pdPC, AL and SEZ of 12 d old and 4 m old ants. **e**, Representative confocal z-projection images of AstA protein in cells and neuropil in the SEZ and CA of 12 d old and 4 m old ants. **f**, Quantification of normalized average *AstA* RNA expression in the whole brain (WB), pdPC, AL and SEZ of 12 d old and 4 m old ants (n = 8 (pdPC, 12 d; AL, 12 d; SEZ, 12 d), 6 (WB, 4 m), or 7 (all other conditions)). Data in **c**,**f** were analysed using one-sided Mann-Whitney tests (**c**: WB: *U* = 11, *p = 0.0487; adPC: *U* = 5, **p = 0.0055; **h**: SEZ: *U* = 4, *p = 0.0152; CA: *U* = 0, **p = 0.0022). ns = not significant; bars represent mean ± s.e.m. Source data

Next, we quantified RNA and peptide levels in the brains of young and old ants (Fig. 4 and Extended Data Fig. 8). We found that, on average, *NPF* RNA expression decreases with age when measured across the whole brain. This effect is due to a large decrease in the expression of *NPF* with age in four cells in the anterior-dorsal protocerebrum (adPC); by contrast, we did not detect a change in *NPF* RNA expression in other brain regions (Extended Data Fig. 8a,c). NPF peptide levels decrease in both the adPC and the antennal lobe, but not in the posterior-dorsal protocerebrum (pdPC) or in the SEZ (Fig. 4a,b,d and Extended Data Fig. 8b). We did not detect changes in *AstA* RNA expression with age, either in the whole brain or in brain subregions (Extended Data Fig. 8d,f). However, AstA peptide levels increased with age in the protocerebrum and antennal lobe (Fig. 4c). We did not detect changes in AstA peptide levels in the SEZ or in the corpora allata (Fig. 4c,e). The age-related decrease in NPF peptide, and increase in AstA peptide, in the protocerebrum and antennal lobe correlate with the results of our pharmacological screen, and are consistent with our finding that NPF promotes nursing and AstA promotes foraging. These age-associated changes in neuropeptide abundance could thus shift the internal state of the brain and underlie division of labour by modulating the neural circuitry that governs brood-care behaviour (Fig. 4f).

## NPF and AstA act in opposite directions

**Fig. 5:**
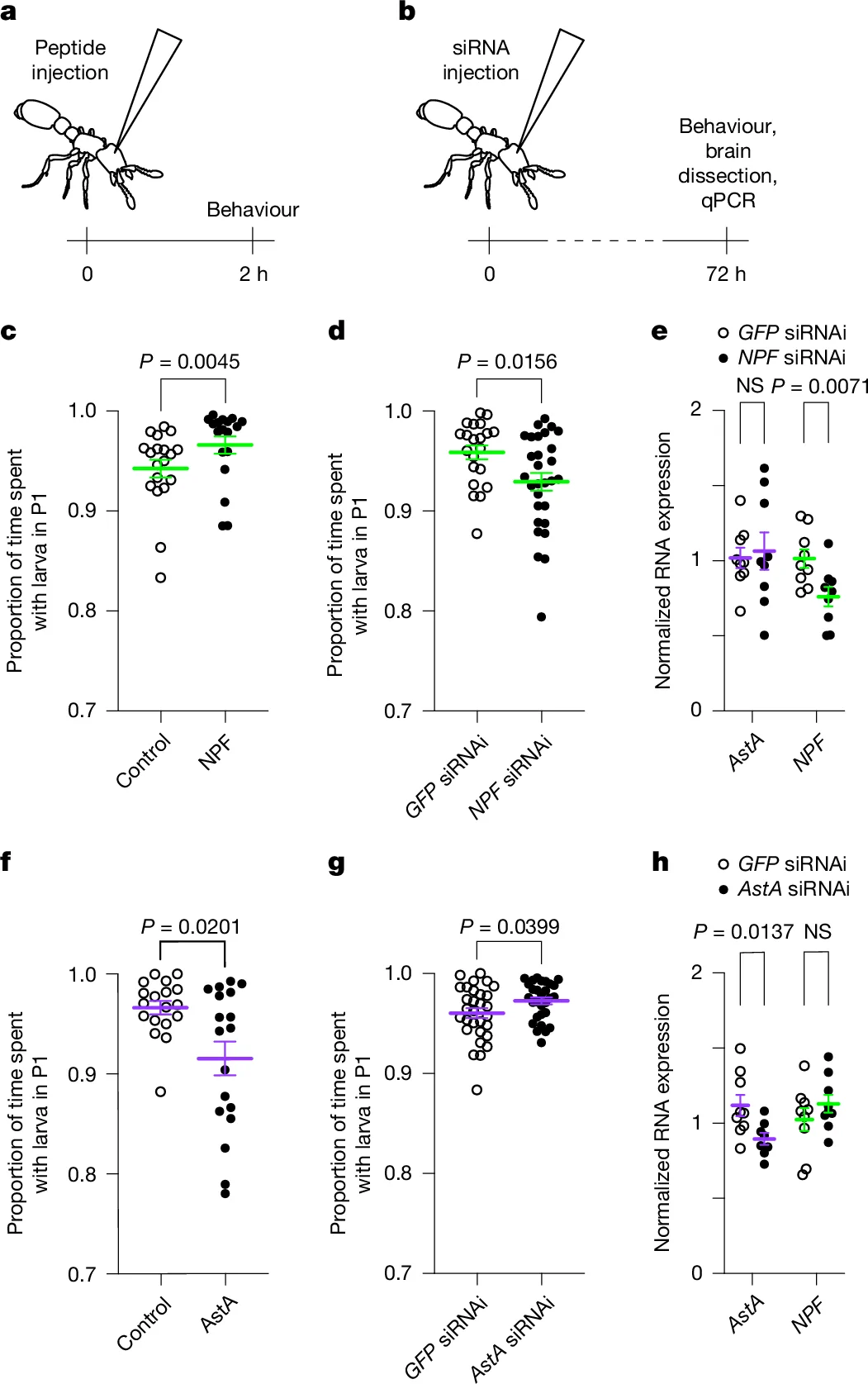
Modulating the balance of NPF and AstA in the brain alters brood-care behaviour. **a**,**b**, Schemas of peptide injection (**a**) and siRNAi knockdown (**b**) experiments. Peptide solutions or siRNAi reagents were injected into the heads of live anaesthetized young (12 days old) ants. Brood-care behaviour was assayed 2 h after peptide injections or 72 h after siRNA injections. After the behavioural assays, brains from siRNA-injected ants were dissected and RNA expression was measured by quantitative PCR (qPCR). **c**,**f**, Quantification of the proportion of time ants physically interact with larvae in P1 after injection of NPF (**c**; *n* = 19 (control) and 18 (NPF) ants) or AstA (**f**; *n* = 18 ants per condition) peptides, compared with solvent control. **d**,**g**, Quantification of the proportion of time ants physically interact with larvae in P1 after injection of siRNA against *NPF* (**d**; *n* = 21 (control) and 29 (*NPF*)) or *AstA* (**g**; *n* = 30 (control) and 29 (*AstA*)), compared with control *GFP* siRNA. **e**,**h**, Quantification of *NPF* and *AstA* RNA expression by qPCR in the heads of ants injected with siRNA against *NPF* (**e**; *n* = 9 samples per condition) or *AstA* (**h**, *n* = 8 samples for AstA expression with *AstA* siRNA and *n* = 9 for all other conditions), compared with ants injected with control *GFP* siRNA. Each sample represents three to four pooled ant heads. Data in **c**–**h** were analysed with one-sided Mann–Whitney tests (**c**, *U* = 86, *P* = 0.0045; **d**, *U* = 195, *P* = 0.0156; **e**, AstA, NS; NPF, *U* = 13, *P* = 0.0071; **f**, *U* = 97, *P* = 0.0201; **g**, *U* = 319, *P* = 0.0399; **h**, AstA, *U* = 13, *P* = 0.0137; NPF, NS). Mean ± s.e.m. Source data

**Extended Data Fig. 9:**
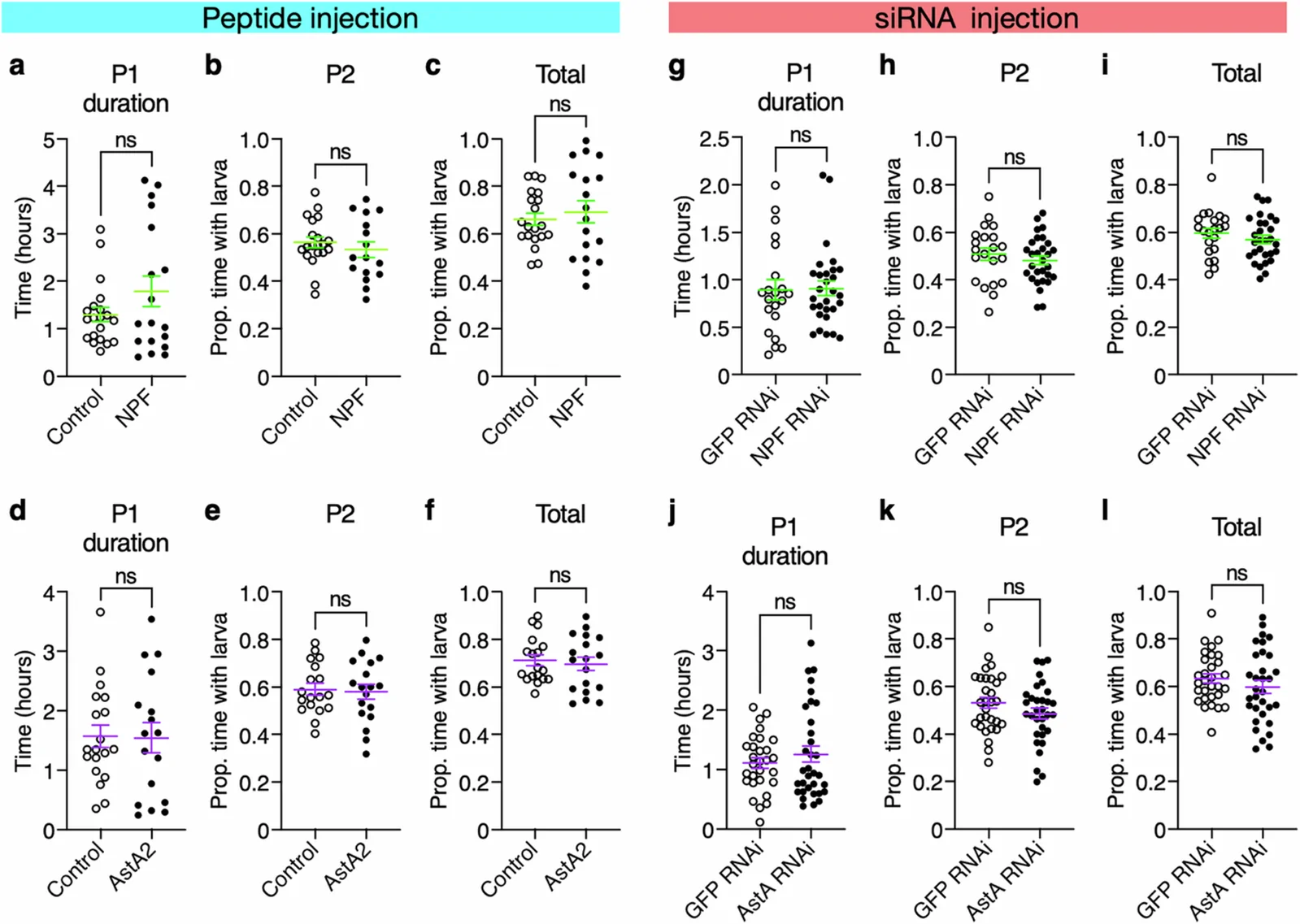
Additional behaviour measurements are not significantly affected by peptide and siRNA injections. **a**–**c**, Quantification of the amount of time ants spend in P1 (**a**, n = 20 (control) and 18 (NPF) ants), and the proportion of time ants interact with larvae in P2 (**b**, n = 20 (control) and 16 (NPF) ants) or over the total assay period (**c**, n = 20 (control) and 18 (NPF) ants) after injection of NPF peptide or vehicle control. **d**–**f**, Quantification of the amount of time ants spend in P1 (**d**, n = 19 (control) and 17 (AstA) ants), and the proportion of time ants interact with larvae in P2 (**e**, n = 18 ants per condition) or over the total assay period (**f**, n = 18 ants per condition) after injection of AstA2 peptide or vehicle control. **g**–**i**, Quantification of the amount of time ants spend in P1 (**g**, n = 21 (GFP) and 29 (NPF) ants), and the proportion of time ants interact with larvae in P2 (**h**, n = 21 (GFP) and 29 (NPF) ants) or over the total assay period (**i**, n = 21 (GFP) and 29 (NPF) ants) after siRNAi knockdown of *NPF* or *GFP* as control. For **g**–**i** total n = 50 ants. **j**–**l**, Quantification of the amount of time ants spend in P1 (**j**, n = 29 (GFP) and 33 (AstA) ants), and the proportion of time ants interact with larvae in P2 (**k**, n = 30 (GFP) and 32 (AstA) ants) or over the total assay period (**l**, n = 30 (GFP) and 32 (AstA) ants) after siRNAi knockdown of *AstA* or *GFP* as control. Data were analysed using two-tailed Mann-Whitney U tests. ns = not significant; bars represent mean ± s.e.m. Source data

To substantiate our screening results, we injected synthetic peptides to increase the levels of NPF and AstA, and small interfering RNAs (siRNAs) to decrease the levels of NPF and AstA. All solutions were injected through the lateral aspect of the heads of young ants. We then measured the responses of the ants to larvae (Fig. 5a,b). Consistent with the results from our pharmacological screen, injecting NPF peptide increased the amount of time that ants interacted with larvae in P1 (Fig. 5c), but had no significant effects on other aspects of the ants’ responses to larvae (Extended Data Fig. 9a–c). Conversely, injecting siRNA against *NPF* decreased the amount of time that ants interacted with larvae in P1 (Fig. 5d), but had no other significant effects (Extended Data Fig. 9g–i). *NPF* siRNA interference (siRNAi) significantly reduced the levels of *NPF* RNA, whereas the levels of *AstA* RNA remained unchanged with this treatment (Fig. 5e).

Injecting AstA peptide decreased the amount of time that ants interacted with larvae in P1 (Fig. 5f), but had no other discernible effects (Extended Data Fig. 9d–f). Conversely, *AstA* siRNAi increased the amount of time that ants interacted with larvae in P1 (Fig. 5g), whereas other aspects of the ants’ responses to larvae seemed to be unaffected (Extended Data Fig. 9j–l). *AstA* siRNAi significantly reduced the levels of *AstA* RNA, whereas the levels of *NPF* RNA remained unchanged (Fig. 5h).

**Extended Data Fig. 10:**
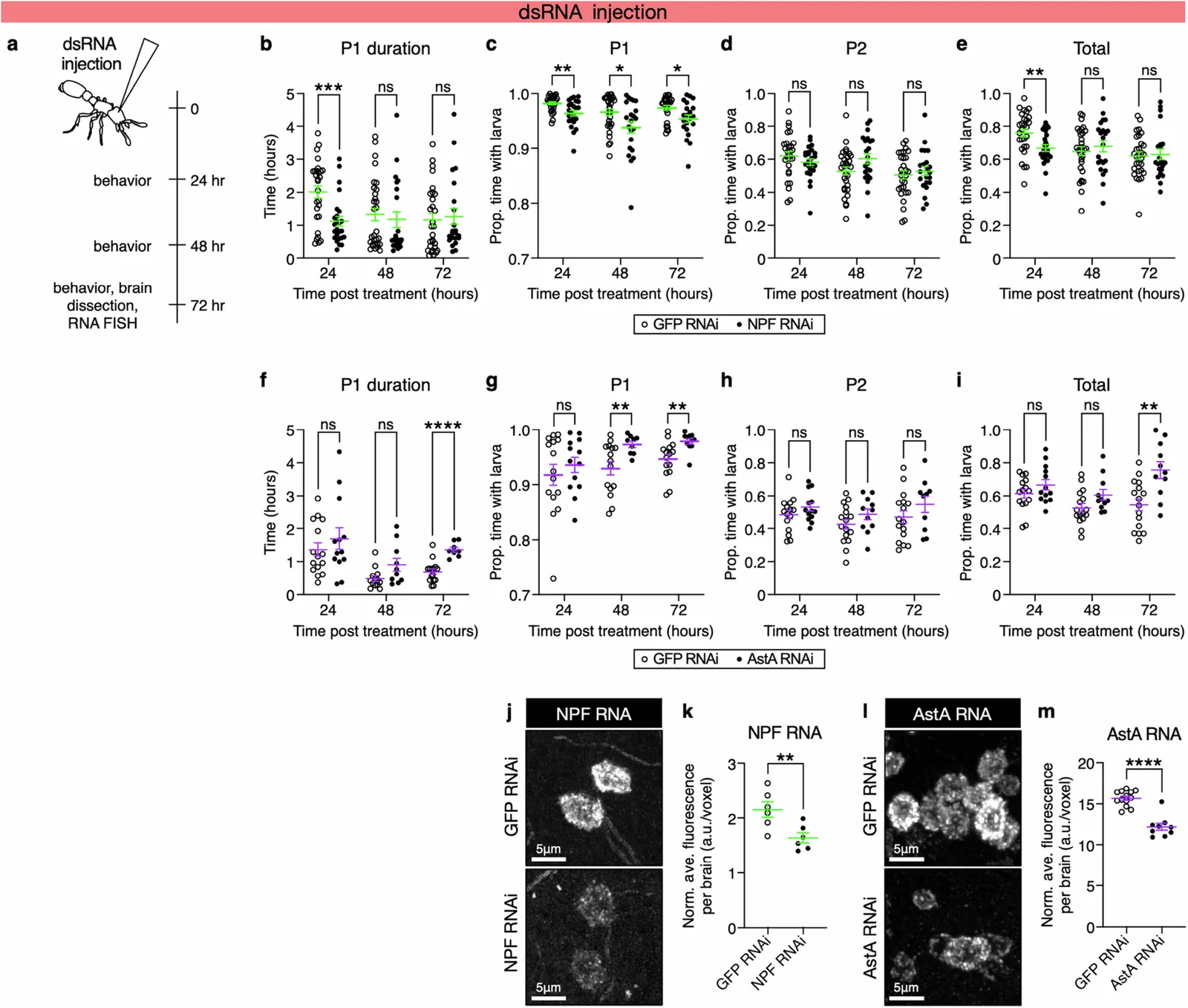
dsRNAi knockdown of *NPF* or *AstA* alters brood-care behaviour. **a**, Schema of experiment. Long double-stranded RNA (dsRNA) was injected into the heads of live anaesthetized ants. These ants were tested in the brood-care assay at 24, 48 and 72 h after injection. Following the 72-hour behavioural test, ant brains were dissected and processed for RNA FISH to quantify knockdown. **b**–**e**, Quantification of the duration of the P1 phase (**b**, n = 28 (GFP, 24 h), 25 (NPF, 24 h), 30 (GFP, 48 h), 22 (NPF, 48 h; NPF, 72 h), and 29 (GFP, 72 h) ants), and the proportion of time ants interacted with larvae in P1 (**c**, n = 27 (GFP, 24 h; GFP, 72 h), 23 (NPF, 24 h), 30 (GFP, 48 h), 22 (NPF, 48 h), and 21 (NPF, 72 h) ants), P2 (**d**, n = 28 (GFP, 24 h), 25 (NPF, 24 h), 30 (GFP, 48 h), 22 (NPF, 48 h; NPF, 72 h), and 29 (GFP, 72 h) ants), and total (**e**, 28 (GFP, 24 h), 25 (NPF, 24 h), 30 (GFP, 48 h), 22 (NPF, 48 h; NPF, 72 h), and 29 (GFP, 72 h) ants) when treated with *NPF* dsRNA or control *GFP* dsRNA. **f**–**i**, Quantification of the duration of the P1 phase (**f**, n = 15 (GFP, 24 h; GFP, 72 h), 13 (AstA, 24 h; GFP, 48 h), 10 (AstA, 48 h), and 8 (AstA, 72 h) ants), and the proportion of time ants interacted with larvae in P1 (**g**, n = 15 (GFP, 24 h; GFP, 48 h), 13 (AstA, 24 h), 9 (AstA, 48 h), 15 (GFP, 72 h), and 11 (AstA, 72 h) ants), P2 (**h**, n = 15 (GFP, 24 h), 13 (AstA, 24 h), 16 (GFP, 48 h; GFP, 72 h), 11 (AstA, 48 h), and 10 (AstA, 72 h) ants), and total (**i**, n = 15 (GFP, 24 h), 13 (AstA, 24 h), 16 (GFP, 48 h; GFP, 72 h), and 11 (AstA, 48 h; AstA, 72 h) ants) when treated with *AstA* dsRNA or control *GFP* dsRNA. **j**, Representative confocal z-projections of *NPF* RNA-expressing cells in the brains of *NPF* dsRNA or control *GFP* dsRNA-injected ants. **k**, Quantification of the normalized average fluorescence per brain of NPF dsRNA or control *GFP* dsRNA-injected ants. n = 6 brains per condition. **l**, Representative confocal z-projections of *AstA* RNA-expressing cells in the brains of *AstA* dsRNA or control *GFP* dsRNA-injected ants. **m**, Quantification of the normalized average fluorescence per brain of *AstA* dsRNA or control *GFP* dsRNA-injected ants (n = 13 (GFP) and 9 (AstA) brains). Data in **b**–**i** were analysed using mixed-effects two-way ANOVAs to account for repeated measures (**b**: Time x RNAi *F*_*(2, 95)*_ = 3.759, *p = 0.0268; **c**: Time *F*_*(1.874, 83.40)*_ = 6.774, **p = 0.0023 and RNAi *F*_*(1, 55)*_ = 14.93, ***p = 0.0003; **d**: ns; **e**: Time *F*_*(1.958, 93.03*_ = 5.649, **p = 0.0051 and Time x RNAi *F*_*(2,95)*_ = 3.122, *p = 0.0486; **f**: Time *F*_*(1.313, 44.66)*_ = 9.638, **p = 0.0015 and RNAi *F*_*(1, 68)*_ = 8.415, **p = 0.005; **g**: Time *F*_*(1.529, 32.11)*_ = 4.561, *p = 0.0260 and RNAi *F*_*(1, 30)*_ = 6.640, *p = 0.0151; **h**: ns; **i**: Time *F*_*(1.893, 71.93)*_ = 3.477, *p = 0.0386 and RNAi *F*_*(1, 76)*_ = 16.85, ***p = 0.0001) followed by Dunn’s multiple comparison tests, of which results for RNAi effects are indicated in the figure panels. Data in **k**,**m** were analysed using one-sided Mann-Whitney tests (**k**: *U* = 3, **p = 0.0076; **m**: *U* = 3, ****p = 1.41e-5). ns = not significant; bars represent mean ± s.e.m. Source data

We confirmed the siRNAi results using an independent RNAi method. In this experiment, we injected long double-stranded RNA (dsRNA) against *NPF* or *AstA* RNA into ant heads to stimulate RNAi knockdown. These long RNA strands are cleaved into siRNA products that cover multiple sites across the target mRNA. This can lead to more robust knockdown than is achieved with the siRNAi method, which targets only a single site per mRNA. We then measured behaviour in the brood-care assay at 24 h, 48 h and 72 h after injection (Extended Data Fig. 10a). Consistent with the siRNAi results, *NPF* dsRNA decreased the amount of time that ants interacted with larvae, especially during P1, whereas *AstA* dsRNA had the opposite effect (Extended Data Fig. 10b–i). Levels of *NPF* and *AstA* RNA were significantly decreased in the brains of dsRNA-injected ants after the 72-h time point, demonstrating that this is an efficient method of gene knockdown (Extended Data Fig. 10j–m). Together, these results show that shifting the balance of NPF and AstA in the brain can recapitulate the age-linked change in responsiveness to larvae.

## NPF and AstA link nutritional status with brood care

**Fig. 6:**
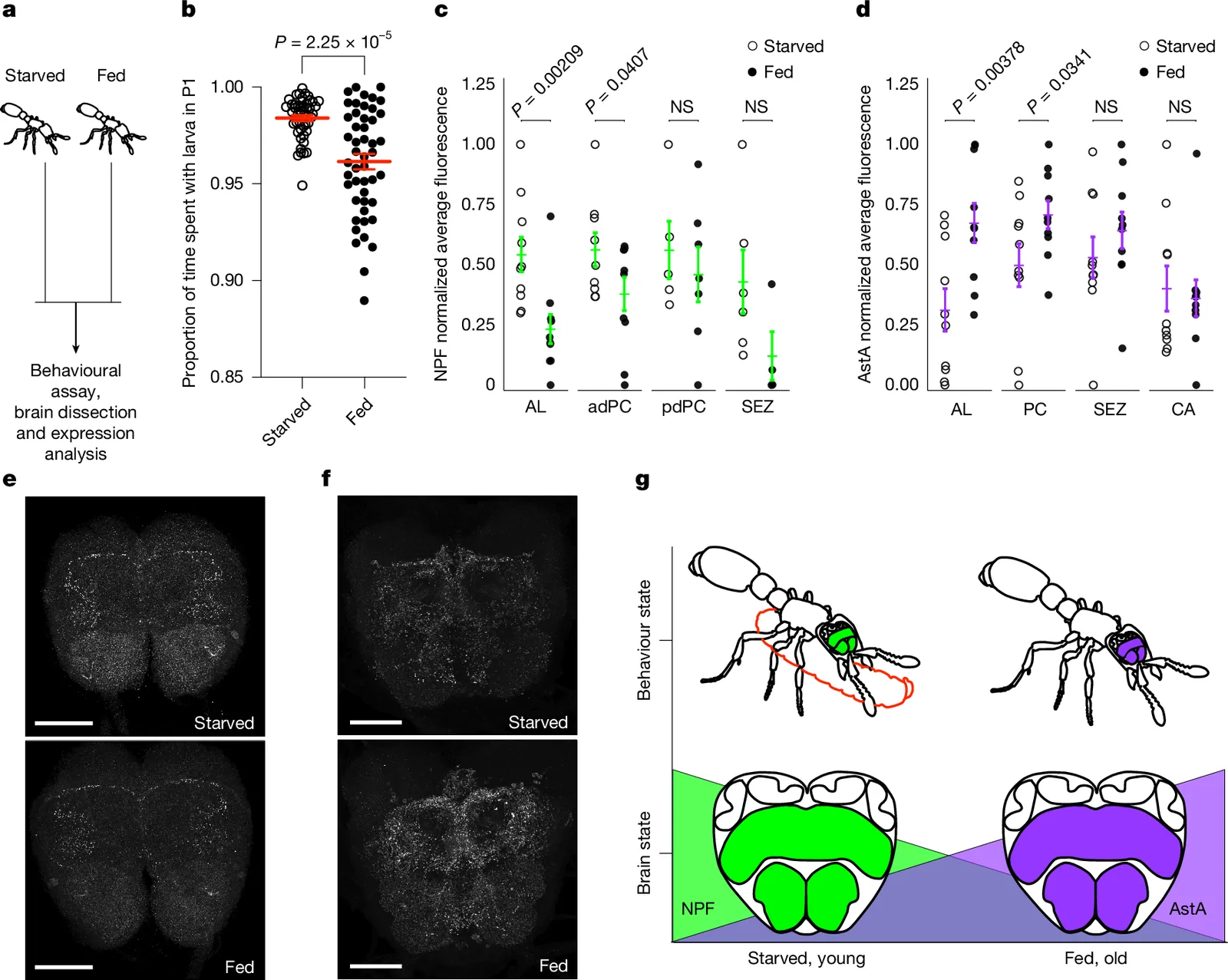
Nutritional status regulates NPF and AstA levels and brood-care behaviour. **a**, Schema of experiment. Two groups of 50 young (12 days old) ants each were fed or starved for 8 days and then tested in the brood-care behaviour assay. Subsequently, their brains were dissected and NPF and AstA peptide levels were measured using antibody staining. **b**, Quantification of the proportion of time that fed and starved ants physically interact with larvae in P1 (*n* = 48 (fed) and 46 (starved) ants). **c**,**d**, Quantification of normalized average NPF (**c**) and AstA (**d**) peptide levels in the brains of fed and starved ants. Measures are shown separately for the antennal lobe (AL, *n* = 10 per condition), adPC (*n* = 9 (starved) and 10 (fed)), pdPC (*n* = 5 (starved) and 7 (fed)) and SEZ (*n* = 6 (starved) and 4 (fed)) for NPF, and for the AL (*n* = 10 per condition), protocerebrum (PC, *n* = 10 per condition), SEZ (*n* = 10 per condition) and corpora allata (CA; *n* = 10 per condition) for AstA. **e**,**f**, Representative frontal view confocal *z*-stack projections of whole brains from fed and starved ants stained for NPF (**e**) and AstA (**f**) peptides. Scale bars, 50 μm. **g**, Model of how nutritional status and age regulate behaviour through neuropeptide levels in the antennal lobe and protocerebrum of the ant brain. Both starved and young ants have high levels of NPF and low levels of AstA and spend more time with larvae (outlined in red). Both fed and old ants have low levels of NPF and high levels of AstA and spend less time with larvae. Data in **b** were analysed with a one-sided Mann–Whitney test (*U* = 564, *P* = 2.25 × 10^−5^). Data in **c**,**d** were analysed with one-sided Welch’s *t*-tests (**c**, AL, *t* = −3.29, df = 17.5, *P* = 0.00209; adPC, *t* = −1.85, df = 16.9, *P* = 0.0407; **d**, AL, *t* = 3.01, df = 17.9, *P* = 0.00378; PC, *t* = 1.69, df = 15.5, *P* = 0.0341). Mean ± s.e.m. Source data

**Extended Data Fig. 11:**
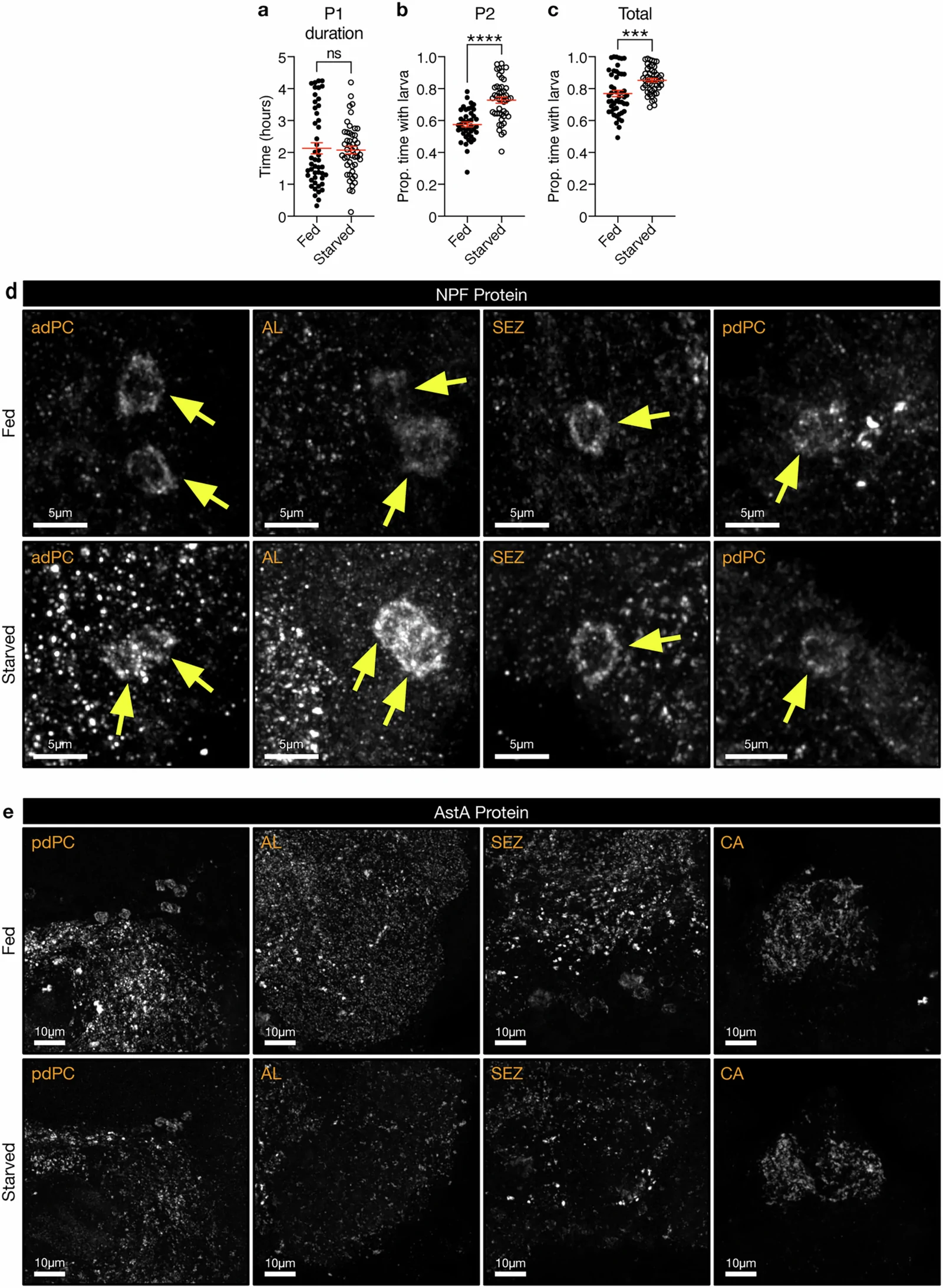
Nutritional status alters brain peptide levels and brood-care behaviour. **a**–**c**, Quantification of the duration of the P1 phase (**a**, n = 48 ants per condition), and the proportion of time ants interact with larvae in P2 (**b**, n = 42 (fed) and 46 (starved) ants), and total (**c**, n = 48 ants per condition) for the fed and starved conditions. **d**, Representative confocal z-projections of NPF protein in cells of the pdPC, adPC, AL and SEZ (yellow arrows) in fed and starved ants. **e**, Representative confocal z-projections of AstA protein in cells and neuropils of the pdPC, AL, SEZ and CA in fed and starved ants. Data in **a**–**c** were analysed using one-sided Mann-Whitney tests (**b**: *U* = 331, ****p = 1.17e-8; **c**: *U* = 704, ***p = 0.0004). ns = not significant; bars represent mean ± s.e.m. Source data

Parental behaviour has evolved repeatedly from ancestors that did not care for their young, and, in various lineages, this transition seems to have involved the co-option of ancestral molecules and neural circuits that govern feeding behaviour^18,43^. NPF and AstA, as well as their orthologues, are widely conserved, and their expression levels are sensitive to nutritional status^23,42,44–48^. These two neuropeptides generally modulate feeding behaviour in opposite directions. Hunger increases the expression of NPF, which promotes feeding, whereas satiety increases the expression of AstA, which inhibits feeding^15,16^. We therefore wondered whether the ancestral link between nutritional status and the expression of NPF and AstA was conserved in *O. biroi*, and, if so, how nutrition would affect brood-care behaviour. To investigate this, we tested starved and fed ants in the brood-care assay and then measured the levels of NPF and AstA peptides in their brains (Fig. 6a). Starved ants spent significantly more time with larvae than fed ants did (Fig. 6b and Extended Data Fig. 11a–c). Notably, no larvae were cannibalized. Starvation increasing brood care might seem surprising because typically, in both solitary and social insects, low nutritional status is associated with increased foraging activity^49,50^. However, this result is consistent with a previous study showing that well-nourished *O. biroi* in fact forage more than starved ones do, possibly owing to evolutionary changes in the underlying endocrine pathways and neural circuits that link nutritional state to foraging behaviour^51^. The inversion of the ancestral relationship between hunger and foraging might relate to the phasic lifecycle of the species, in which workers undergo regular periods of hunger during a reproductive phase in which foraging activity ceases. These periods are then followed by a foraging phase in which both foraging and feeding increase^27^.

The levels of both NPF and AstA changed with nutritional status, and these changes recapitulated what is known from solitary animals. In starved ants, NPF increased in the adPC and antennal lobe, but not in the SEZ or pdPC (Fig. 6c,e and Extended Data Fig. 11d,f). In fed ants, AstA increased in the protocerebrum and antennal lobe, but not in the SEZ or corpora allata (Fig. 6d,f). The behavioural responses of starved and fed ants to larvae were thus associated with NPF and AstA levels in a manner consistent with our experimental manipulations of these peptides (Fig. 5 and Extended Data Fig. 10). Indeed, both age and nutritional status shift the neuromodulatory state of the ant brain between NPF-high–AstA-low and NPF-low–AstA-high conditions in the protocerebrum and antennal lobe (Figs. 4 and 6). In *O. biroi*, a conserved and ancient neuromodulatory system has thus been co-opted to regulate brood-care behaviour, producing the distinct alloparental responses of young and old ants to larvae (Fig. 6g).

## Discussion

Our results show that the neuropeptides NPF and AstA, which regulate feeding in solitary animals, have been evolutionarily co-opted to regulate alloparental care in the clonal raider ant. Nutritional status altered the expression of these neuropeptides in the brain and, in parallel, modulated brood-care behaviour, indicating that the ancestral link between nutrition and the expression of these neuropeptides is maintained alongside their novel roles in caregiving. Expression of NPF and AstA also changed with age, consistent with the effects on brood care revealed by our functional manipulations. These findings imply that, in addition to governing alloparental care, NPF and AstA mediate the age-related shift from nursing to foraging.

AstA and NPF form part of an ancient, deeply conserved and highly pleiotropic regulatory network^41,52^. This network centres around insulin, juvenile hormone (JH) and ecdysone, and co-ordinates feeding, growth and reproduction with nutritional state. Insulin modulates the synthesis of both JH and ecdysone, whereas JH and ecdysone are mutually antagonistic and ecdysone antagonizes the insulin and insulin-like signalling pathway. NPF generally promotes JH synthesis, although the details of this relationship can vary between taxa^53,54^. In *Drosophila*, gut-derived NPF stimulates insulin production, and the action of NPF-responsive neurons is inhibited by insulin, forming a negative-feedback loop that co-ordinates feeding with nutritional status^55,56^. AstA generally inhibits both JH synthesis and NPF neurons, and stimulates insulin-producing cells^16,57,58^.

Our data fit with an emerging pattern whereby elements of this regulatory network have been consistently co-opted to govern social behaviour in ants and other insects^24^. Both insulin and JH regulate the transition from nursing to foraging across social Hymenoptera^59–62^. Other elements of this network have been shown to have important regulatory roles in ant species with social structures that differ from that of *O. biroi*^13,62,63^. In the leaf-cutter ant *Atta cephalotes*, labour is divided between morphologically distinct worker castes. Majors specialize in colony defence, whereas minors specialize in brood care. The behavioural differences between these castes are maintained by the neuropeptide neuroparsin A (NPA), which is synthesized by insulin-producing cells in the brain and interacts with insulin signalling^13^. NPA also seems to regulate the reproductive division of labour across social insects^64^. In the ponerine ant *Harpegnathos saltator*, worker behavioural roles are controlled by the neuropeptide corazonin^14^. Corazonin is homologous to vertebrate gonadotropin-releasing hormone, and in *Drosophila melanogaster*, corazonin neurons modulate the activity of insulin-producing cells^65^. In the carpenter ant *Camponotus floridanus*, behavioural differences between majors and minors are controlled by a JH esterase in glial cells, which constitute the blood–brain barrier and control JH titres in the brain^62^. Across species, these age- and behaviour-associated patterns are likely to be underpinned by epigenetic modifications^66,67^. Together, this suggests that NPF and AstA modulate brood-care behaviour through their interactions with insulin and JH signalling, and helps explain why nutritional state, ageing and the division of labour are so intricately intertwined in social insects^68^.

Parallel research into the regulation of vertebrate parental care has found that, as in social insects, molecular factors that ancestrally governed feeding, growth and reproduction have been convergently co-opted to regulate caregiving. The neuropeptide NPY, the vertebrate homologue of NPF, is a primary driver of feeding, and seems to have been co-opted to modulate caregiving at independent evolutionary origins of parental behaviour in frogs, birds and mammals^69–71^. Virgin female mice typically care for pups, but will show aggression towards them when hungry. This switch is mediated by the release of NPY, which inhibits care-promoting neurons in the medial preoptic area of the hypothalamus^72^. Galanin, the receptor for which is homologous to the insect AstA receptor, is also expressed by hypothalamic neurons in mice, and optogenetic manipulation of these cells can toggle male behaviour between parental care and pup-directed aggression^71,73,74^.

Various overlapping hypotheses have been proposed to explain the molecular evolution of social insect behaviour. The ovarian ground-plan and reproductive ground-plan hypotheses predict that molecular factors that governed foraging and reproduction ancestrally have been co-opted to control queen–worker dimorphism^20,75,76^. The maternal heterochrony hypothesis posits that worker behaviour evolved through the molecular mechanisms that governed parental care ancestrally becoming active in females without having reproduced^77^. Our data, together with recent findings from other taxa, support these hypotheses and simultaneously reveal them to capture only part of a broader evolutionary trend: an ancient and deeply conserved regulatory network pleiotropically controls feeding, growth and reproduction. Independent origins of parental (and/or alloparental) behaviour from insects to mammals, seem to have convergently co-opted elements of this network^21,22,24^. This suggests that the evolution of parental (and/or alloparental) behaviour is mechanistically more constrained than previously thought^24^.

Although similar molecular factors seem to have been co-opted recurrently across taxa, there are some notable species-specific effects. For example, parental care is promoted by JH in certain insect taxa and suppressed by JH in others^78^. The fact that NPF promotes nursing in *O. biroi* is surprising. In most social insect species, JH inhibits nursing, and in model insects, NPF promotes JH, suggesting that NPF should inhibit brood care. In *O. biroi*, one of these ancestral molecular relationships either has been inverted or is outweighed by a counterbalancing force. Understanding how NPF and AstA interplay with insulin and JH in *O. biroi* brood care will shed light on how ancient molecular networks are tweaked during the evolution of complex social behaviours. Species specificities in these relationships might reflect the varied ways in which caregiving relates to feeding and reproduction. There is substantial diversity in adult–larva interactions across ants that could be used to explore such effects. For example, in certain contexts, adults feed larvae mouth-to-mouth, and interacting with larvae reduces adult nutritional status^79^. In other contexts, larvae regurgitate pre-digested food to feed adults, or adults pierce the sides of larvae and drink their haemolymph, in which case interacting with larvae increases adult nutritional status^80^. In some ant lineages, reproduction is phasic and larvae inhibit egg laying, whereas in others, reproduction is continuous and there is no such inhibition^50,51^. Ultimately, understanding species-to-species variation in the regulation of caregiving will enable an ecologically informed understanding of how social behaviours evolve.

## Methods

### General ant husbandry and maintenance

*Ooceraea biroi* colonies of clonal line B^81^ were housed in 0.6-quart ClickClack boxes with an approximately 3-cm-deep plaster of Paris floor. Colonies were kept in a climate-controlled environmental room at around 25 °C and around 60% humidity, and the plaster floor was kept damp by regularly adding water. *O. biroi* colonies cycle between a reproductive phase during which colonies contain eggs and pupae and ants do not forage, and a foraging phase when larvae are present and ants forage for food^27^. An entire colony cycle takes around 5 weeks. Towards the end of the reproductive phase, eggs hatch into larvae. The foraging phase begins when larvae enter the third instar about 1 week later, coinciding with the emergence of the previous cohort of callow workers. During the foraging phase, which lasts around 2 weeks, colonies are composed entirely of workers and third and/or fourth instar larvae. In this phase, colonies were fed three times per week with frozen fire ant (*Solenopsis invicta*) brood. At the time of feeding, the plaster floor was briefly cleaned with Q-tips soaked in 10% bleach, and the plaster was then watered.

### Ant maintenance for experiments

Naturally cycling *O. biroi* colonies contain several generations of adults. During the foraging phase, the youngest generation of ants are easily recognizable by their lightly melanized cuticle for up to 6–7 days after they eclose from pupae. For the primary and secondary neuropeptide screens, 300–500 6-to-7-day-old ants were separated from their colonies into new boxes together with larvae in an approximately 1:1 ratio and aged to 12 days old. At that time, they were tested in the screens. For all other experiments, we generated age-matched colonies of ants by seeding new colonies with 300–500 6-to-7-day-old ants and larvae in an approximately 1:1 ratio. At each subsequent foraging phase, the newly eclosed callow ants were removed and transferred into new colonies with larvae in an approximately 1:1 ratio. This procedure was repeated for every foraging phase in every colony. Ants in all resulting colonies were aged up to 5 months old. This resulted in a regular supply of age-matched ants that were 12 days old, and 1, 2, 3, 4 and 5 months old, as well as larvae for experiments. All ants and larvae used in experiments came from colonies in the foraging phase when the larvae were in the fourth instar, and 12–16 days after callow workers had eclosed. For one week before behavioural experiments, ant colonies were fed every other day with frozen *S. invicta* brood stained with 0.5% weight/volume bromophenol blue (Sigma Aldrich; B5525) in autoclaved reverse-osmosis (RO) water. This stain accumulates in the larval gut, increasing the visual contrast and thus facilitating automated tracking (see below). Ant colonies were last fed 24 h before behavioural experiments.

### Neuropeptidome annotation

Lists of neuropeptide precursor protein sequences were compiled from literature focusing on insects^31–36,82–101^, as well as from a broad survey of neuropeptides across metazoan phyla^23^. We identified putative *O. biroi* neuropeptide precursor proteins by a BLASTp homology search of the compiled lists against the *O. biroi* non-redundant protein sequences (nr) database using default settings. Precursor proteins with high degrees of certainty were retrieved from additional insect species, with a focus on Hymenoptera. Homology of candidate *O. biroi* neuropeptide precursor proteins to query sequences was confirmed through reciprocal BLASTp on the nr database of the query sequence species. Signal peptides were identified using SignalP 6.0 (https://dtu.biolib.com/SignalP-6). Neuropeptides were annotated manually by identification of stereotypical proteolytic cleavage site motifs (RR, RK, KR and KK) and alignment to homologous neuropeptides from representative insect species (Supplementary Tables 1–3). Post-translational modifications were predicted by comparison with annotations of homologous insect neuropeptides in the UniProtKB database (https://www.uniprot.org). These annotations were performed in 2021 and reflect the neuropeptidome literature at that time.

### Synthesis, storage and preparation of neuropeptides

Neuropeptides of 35 amino acids or fewer in size were synthesized including known modifications such as amidation and cyclization. For the neuropeptide screen treatment control, a 31-amino-acid neuropeptide, DH31, was synthesized with a biotin tag at the Proteomics Resource Center at Rockefeller University and provided lyophilized at 15% purity. Lyophilized DH31–biotin was resuspended in pure dimethyl sulfoxide (DMSO) to 30 mM concentration, aliquoted and stored at −80 °C before use. For the primary round of screening, neuropeptides were synthesized at the Proteomics Resource Center at Rockefeller University and provided lyophilized at 15–92% purity. We opted for this low level of purity of neuropeptides in the primary screen because of the large number of peptides and the costs associated with achieving high-purity preparations. This implies, however, that the rates of false positives in this screen might have been high, which we then mitigated by a stringent secondary screen using high-purity neuropeptides (see below). Lyophilized neuropeptides were stored for up to 4 months at −80 °C before resuspension. Stock solutions were made by resuspending lyophilized neuropeptides in pure DMSO to 2 mM–100 mM, depending on solubility, and then aliquoted and stored at −20 °C for up to 6 months before use. For the experiment to test whether the size range of our neuropeptide screening library could penetrate the cuticle, 30 mM DH31–biotin stock solution was diluted in autoclaved RO water to a final concentration of 1 mM DH31–biotin and 1% DMSO. The validation was then performed by soaking ants in biotinylated neuropeptides, dissecting out their brains and treating the brains with fluorescent-dye-conjugated streptavidin before performing quantitative confocal microscopy. For the primary neuropeptide screen, stock solutions of neuropeptides were diluted in autoclaved, RO-purified water to a final concentration of 1 mM neuropeptides and 1% DMSO. Control solutions were 1% DMSO in autoclaved RO water.

For the secondary round of dose–response analysis screening, neuropeptides were synthesized by Bio-Synthesis and provided lyophilized at higher than 95% purity. Stock solutions were made by resuspending lyophilized neuropeptides to a concentration of 10 mM in pure DMSO, and then aliquoted and stored at −20 °C for up to 4 months before use. For all experiments, fresh aliquots of stock solutions were diluted to avoid freeze–thaw cycles that compromise protein integrity. For the secondary screen, stock solutions were diluted to test concentrations of 0.1 mM, 0.3 mM, 1 mM and 3 mM neuropeptides in 1% DMSO and autoclaved RO water. For the repeat of the NPF dose–response experiment, stock solution of NPF was diluted to 0.003 mM, 0.01 mM, 0.03 mM, 0.1 mM, 0.3 mM, 1 mM and 3 mM NPF in 1% DMSO. Control solutions were 1% DMSO in autoclaved RO water.

### Colony experiment

Colonies were housed in two-chamber arenas constructed from sandwiched layers of laser-cut acrylic and Tyvek cut to 100 × 100mm (length (L) × width (W)). From the bottom up, a base layer of 3-mm-thick acrylic was topped by a hollow, 6-mm-thick rim layer. The internal space of this layer was filled with plaster of Paris, which was kept moist. This was topped by a layer of water-vapour-permeable Tyvek that covered the plaster of Paris in one chamber but left it exposed in the other chamber. Because the ants prefer to nest on plaster of Paris, this partial Tyvek covering defines one chamber as the nest and the other chamber as the foraging arena. A layer of 3-mm-thick acrylic in which two rectangular 53 × 24mm (L × W) chambers were cut was placed on top of the Tyvek layer, forming the walls of the arena. The two chambers were connected by an entrance tunnel of 8 × 2mm (L × W). A final layer of 3-mm-thick clear acrylic was used to seal the arena, with a lid cut over each chamber so that they could be opened independently. Colonies of 60 ants (30 that were 12 days old; 30 that were 4 months old) and 60 larvae were transferred into these arenas after all ants were paint-marked with a unique two-dot colour code. Three such set-ups were constantly video-recorded at ten frames per second for 3 days. For each replicate, behaviour was scored manually in single frames subsampled every 8 h (nine frames). Frames either side of the focal frame were used to disambiguate behaviour in the focal frame. The following behaviours were scored: foraging (an ant is in the foraging arena when all larvae are in the nest) and physical contact with larvae. Physical contact with larvae was then subdivided into guarding (standing over a larva and stationary, with the caveat that this posture has not been functionally linked to aggressive responses towards threats); manipulating (mandibles and antennae in contact with larva and rapid movement of these body parts); antennating (antennal contact only, with antennae stretched forwards to contact the larva); carrying a larva; and incidental (not obviously tending a larva—either making contact as walking past or standing stationary in contact with the larva but oriented away from it).

### Brood-care behaviour units, recording set-up and assay

For the neuropeptide screening and nutritional status behavioural experiments, the brood-care behavioural arenas were constructed from sandwiched layers of laser-cut acrylic and Tyvek cut to 10 × 10 cm (L × W) (Extended Data Fig. 1a). From the bottom up, a base layer of 3-mm-thick acrylic was topped by a hollow rim layer of 6-mm acrylic into which water-dampened cotton gauze was placed. This was then topped by a layer of 1.5-mm-thick acrylic mesh, followed by a layer of Tyvek that provided the floor of the assay chambers. A layer of 6-mm-thick acrylic in which 24 radially symmetrically arranged 30 × 2 mm (L × W) rectangles were cut was then placed on top of the Tyvek layer, forming the walls of the assay chambers. For the peptide injection and RNAi experiments, the hollow rim layer was filled with water-dampened plaster of Paris that replaced the cotton gauze, acrylic mesh and Tyvek layers and provided the chamber floor. These layers were secured by acrylic screws, and each unit was then secured by metal screws to a tray. At this point, larvae were loaded into the centre of each chamber, followed by treated ants. Each unit was then topped with a layer of clear, antistatic 3-mm-thick acrylic that formed the lid, secured to the units by screws. Between experiments, the lid and chamber layers were washed in hot water with antibacterial soap free of dyes and odorants, and the Tyvek or plaster floors were replaced. Each tray held 8 units of 24 assay chambers each. Trays were placed within a light-protected box containing 8 webcams (Logitech C910) positioned around 10 cm above the centre of each unit such that all 24 chambers per unit were within view for video recording. Lighting in the box was provided by white light-emitting diodes. Units were filmed at 5 frames per second and 2,592 × 1,944-pixel resolution for 5 h. The recording boxes were setup in a climate control room at around 25 °C and around 60% humidity.

### Neuropeptide screening

In the screening assays, 12-day-old ants were removed from their colony using soft forceps and placed immediately into 1.5-ml Eppendorf tubes on ice, resulting in near instant anaesthesia. Solutions of neuropeptides were then added to the tubes. The tubes were briefly and vigorously shaken to completely submerge all ants in the solution and then placed back on ice for 20 min. The tubes of solution with ants were then poured onto dry paper towels, which pulled excess solution off the ants. Ants were then placed into the assay chambers for filming. In the primary screen, we tested each of 61 neuropeptides on *n* = 12 ants and compared their behaviour with that of *n* = 12 ants treated with control solution. The primary screen was designed to be high throughput and low stringency, with the goal of identifying a shortlist of candidates for a more rigorous secondary dose–response screen. Given that the neuropeptide solutions used in this screen were low quality (see ‘Synthesis, storage and preparation of neuropeptides’) and the statistical analyses were permissive, this approach is prone to produce many false negatives and false positives. In the secondary screen, the treatment method was the same as that used in the primary round, but each candidate neuropeptide was synthesized at high quality, tested on *n* = 24 ants per concentration and compared with *n* = 24 ants treated with control solution.

### Extra behavioural control experiment

In separate control experiments, we compared the responses of adult ants to live *O. biroi* larvae, dead *O. biroi* larvae, dead *O. biroi* larvae washed in hexane, acrylic beads and fire ant (*S. invicta*) larvae (that is, prey items). For this experiment, a colony of about 500 6-to-7-day-old ants and larvae in an approximately 1:1 ratio was established and fed every other day with bromophenol-blue-stained *S. invicta* brood. When ants were 11 days old and larvae were in the fourth instar, two groups of around 100 larvae each were separated from the colony for treatments. One group of larvae was frozen overnight at −80 °C to kill them. These dead larvae were thawed at room temperature before the behavioural experiment. The larvae in the second group were placed in a glass scintillation vial and washed five times in pure hexane. These larvae were stored in hexane overnight at 4 °C and then dried on paper towels at room temperature for 20 min to allow residual hexane to evaporate before the behavioural experiment. Small (approximately 0.5-mm diameter), roughly cylindrical beads were laser cut from 3-mm pink acrylic. These beads were approximately the size of a late fourth instar *O. biroi* larva.

### Behavioural tracking

Automated behavioural analyses of the single ant assays were performed using anTraX^102^, yielding (*x*,* y*) position co-ordinates for both the ant and the larva for each assay chamber. We wrote custom scripts that automatically calculated several measures used for analysis and tracking quality verification. These included an interaction score (1 if ant and larva positions aligned; 0 if not), the frame of larva detection (details below) and the percentage of frames in which the ant and larva identities were successfully classified. In 92% of chambers across all experiments, ant and larva identities were maintained in 99% or more of the video frames. We excluded data from chambers in which either the ant identity or the larva identity was maintained in 80% or less of the frames. However, this occurred rarely—in only 2.8% of chambers. In 5.2% of chambers, identities were maintained in 81–98% of frames. In these cases, most instances of identity and positional data loss occurred when an ant was carrying the larva and therefore occluding it from view. In these cases, the larva’s position data was manually corrected to match the ant’s position data over the frames from which the larva position was lost. Tracking data were visualized using Python with the packages numpy, pandas, scipy, matplotlib and seaborn. Scripts and example datasets for behavioural analyses and visualizations are available via GitHub (https://github.com/Social-Evolution-and-Behavior/Paul_Kay_Kronauer_2026).

### Annotation and analysis of behavioural tracking data

To provide a broad overview of how ants interact with larvae in the one-on-one assay, we manually annotated behaviour on the basis of an evenly distributed subsample of 24 frames per individual for a total of 47 ants across 4 conditions: 12-day-old ant with an *O. biroi* larva (*n* = 12), 4-month-old ant with an *O. biroi* larva (*n* = 12), 12-day-old with an *S. invicta* larva (*n* = 12) and 4-month-old ant with an *S. invicta* larva (*n* = 11). Physical contact with larvae was scored for each frame and subdivided into guarding, manipulating, antennating, carrying and incidental, as for the colony assay. From the results of this manual scoring, physical contact with larvae emerged as a robust proxy for brood-care behaviour and therefore motivated and justified the subsequent use of an automated image-processing pipeline to extract physical contact data. Relevant behaviour begins when the ant first detects the larva, typified by antennal drumming on the larva and physical engagement with it (Supplementary Video 1). Larva detection was defined as the moment in frames when the (*x*, *y*) co-ordinates of the ant and larva first converge. Larva detection was confirmed by comparing the visualized data with the raw video for every ant, and errors were corrected manually. We defined the P1 behavioural phase as the period beginning with larva detection and continuing while the ant remains mostly in physical contact with the larva, irrespective of whether the ant is carrying the larva around the assay chamber or remaining in one location. Larva carrying did not consistently occur across all assays or conditions, and it was therefore collapsed into the P1 phase. The P2 phase was defined as the period when the ant begins to leave the larva and subsequently spends most of its time away from the larva. This phase continued until the end of the video recording. We determined the transition point between the P1 and P2 phases algorithmically for each ant. In brief, we first converted the ant and larva (*x*,* y*) positions into single values ranging between 0 mm and 30 mm (the length of the assay chamber). We saved these values as ‘PositionData’. Then, beginning from the frame of larva detection, we applied a 200-frame (40-s) sliding window over the ant’s PositionData, in which we calculated the difference between the ant’s absolute minimum and absolute maximum positions. We ignored the ant’s PositionData in frames in which the ant was physically interacting with or carrying the larva, to only capture PositionData from frames in which the ant was away from the larva. We saved these values as the ‘AntMaxDistance’. We then smoothed the AntMaxDistance data by applying a second 400-frame (80-s) sliding window in which we averaged the measures. We calculated the first inflection point of the smoothed AntMaxDistance data (that is, the frame at which the data first exceed half of the absolute maximum value of the data) and defined this frame as the transition point between the P1 and P2 behavioural phases. In rare cases (less than 1%) in which the transition point occurred at fewer than 1,000 frames from the moment of larva detection, we defined the transition point as 0 and the ant as having no P1 phase. These cases were excluded from the analysis of the P1 phase and phase transitions as outliers. In other rare cases (less than 5%), ants would carry the larva from the moment of larva detection to the end of the assay period. In these cases, the entire interaction period was defined as P1, the length of this period was taken as the P1 duration and, because there was no transition point and no P2 phase, we excluded these cases from analyses of the P2 phase. We then quantified the duration of the P1 phase (time from first detection of larva to the transition point) and the proportion of time that the ant was in physical contact with the larva in each of the P1 and P2 phases, as well as over the total assay period. Scripts and example datasets used for these analyses are available via GitHub (https://github.com/Social-Evolution-and-Behavior/Paul_Kay_Kronauer_2026).

### Sample size and statistical analyses

We used power analyses to determine the sample size for all experiments. For the primary round of the neuropeptide screen, power analysis (parameters: 0.80 power, *α* = 0.05, two-sided comparison of means) showed that *n* = 12 ants per condition would give a power of 0.99, with an effect size based on estimates of mean and standard deviation from the average total proportion of time that young and old ants spent with larvae during the brood-care behaviour assay (Fig. 2g). We analysed the primary screen results with a *t*-test comparison of means, without correction for multiple comparisons or false discovery rate. Although this permissive statistical method increased false positives, it accommodated small effect sizes and optimized the sample size for throughput, allowing for a comprehensive test of the neuropeptidome. Effect sizes (Cohen’s *d*) of neuropeptide candidate behavioural measures (P1 duration, P1, P2 and total) were calculated as *d* = (mean_neuropeptide_ − mean_control_)/(standard deviation_control_).

In the secondary round of screening, power analysis (parameters: 0.80 power, *α* = 0.05, six groups, ANOVA) showed that a minimum of *n* = 12 ants per treatment would give a power of 1, with estimates of the *F* effect size taken from the time-course analysis of brood-care behaviour (Extended Data Fig. 1c–f). We used *n* = 24 ants per condition in the dose–response experiments of the secondary round of neuropeptide screening. For the experimental paradigms in which we compared mean NPF or AstA brain fluorescence measures, power analysis (parameters: power 0.80, *α* = 0.05, two-sided comparison of means) showed that a minimum of *n* = 5 brains per conditions would yield a power of 0.9, using estimates of mean and standard deviation from the AstA IHC aging analysis (Fig. 4c, AL measure). On the basis of these analyses, we used a minimum of *n* = 20 ants for all additional behavioural experiment paradigms (peptide injections, dsRNAi, siRNAi, fed or starved behaviour) and from these sampled a minimum of *n* = 5 brains or samples per condition for quantitative confocal microscopy and qPCR analyses, respectively. For all experiments, outliers were identified (ROUT method with *Q* = 1%) and removed before statistical testing. In some cases, data were normalized by square root transformation and statistical analyses were performed on the transformed data. Details on statistical analyses and the final sample sizes used for the data plotted in graphs are in the respective figure legends. Graphing and statistical analyses of data were performed using GraphPad Prism (versions 8–10).

### Brain dissection and tissue preparation

Live ants were dipped in 95% ethanol for 5–10 s and then submerged in sterile filtered 1× phosphate-buffered saline (PBS) in a silicone-coated Petri dish under a dissection microscope. The antennae and mandibles were cut off using microdissection scissors, and the head cuticle was gently opened using forceps to reveal the brain. The brain was lifted from the ventral head cuticle, removing the oesophagus, which runs through the brain. Small tracheae on the surface of the brain were then gently removed. Brains were transferred into fixative (sterile filtered 1× PBS, 4% paraformaldehyde) using glass Pasteur pipettes that had been pre-lubricated with sterile filtered 1× PBS and 0.1% Tween. Brains for RNA FISH were fixed overnight at 4 °C. Brains for IHC were fixed for 2 h at room temperature. Fixative was removed with three quick washes followed by three 20-min washes in sterile filtered 1× PBS. Brains were either processed immediately for staining, or stored in sterile filtered 1× PBS, 0.02% sodium azide for up to 2 weeks at 4 °C.

### RNA FISH staining

For RNA FISH staining of NPF and AstA, we obtained nucleic acid probes, fluorescently tagged hairpin amplifier sequences and buffers from Molecular Instruments. Brains were stained according to the Molecular Instruments HCR 3.0 Protocol. In brief, fixed brains were incubated in probe hybridization buffer for 30 min at 37 °C, and 8 pmol of NPF or AstA probes was added. The samples were incubated at 37 °C for 48 h rotating on a thermoblock at 300 rpm. Probes were removed with five 10-min washes of probe wash buffer, followed by two 5-min washes with sterile filtered 5× SSCT (5× sodium chloride citrate and 0.1% Tween-20). Samples were then switched into amplification buffer and incubated for more than 10 min at room temperature. Subsequently, 6 μl of 3 μM stock of each fluorescently tagged hairpin amplifier was prepared in separate tubes, heated to 95 °C for 90 s and then cooled to room temperature in the dark for 30 min. The appropriate hairpin sets for the probes were added to 100 μl of amplification buffer, transferred to the samples and incubated overnight in the dark at room temperature on a benchtop rocker. The next day, the amplification step was stopped by adding excess 5× SSCT, followed by five 10-min washes in 5× SSCT. The samples were then switched into sterile filtered 1× PBS through three quick washes, and brains were incubated in DAPI (1:500 of a 1 mg ml^−1^ stock solution) in sterile filtered 1× PBS for 10 min, followed by three 20-min washes in sterile filtered 1× PBS. Stained brains were mounted on silane coated glass slides (Electron Microscopy Sciences, 63411-02) in SlowFade Glass AntiFade mounting medium (Invitrogen, S36917).

### Antibodies and IHC staining

For IHC staining of AstA, we used a polyclonal rabbit-anti-AstA antibody from Jena Biosciences (anti-A-AST, ADB-062). For IHC of NPF, we used a custom polyclonal chicken-anti-NPF antibody generated by YenZym through their Chicken Antibody Service with IgY extraction and purification. Chicken-anti-NPF was raised against the *O. biroi* NPF neuropeptide sequence (YLDLVREYYSMTGTARF-amide). The custom chicken-anti-NPF antibody is available upon request. We used secondary donkey-anti-rabbit and donkey-anti-chicken antibodies conjugated to either a488 or a594 fluorophores (Jackson ImmunoResearch, codes: 711-545-152, 703-545-155, 711-585-152 and 703-585-155). Antibody solutions were diluted 1:1 in pure glycerol and stored at −20 °C. For IHC staining, fixed brains were incubated in blocking solution (sterile filtered 1× PBS, 0.1% Triton X-100, 0.02% sodium azide, 5% donkey serum) for 1 h. Primary rabbit-anti-AstA at a final concentration of 1:1,000, or chicken-anti-NPF at a final concentration of 1:2,000, was then added, and brains were incubated for a further 7 days. Primary antibodies were then washed off in three quick washes followed by three 20-min washes in sterile filtered 1× PBS. Brains were again incubated in blocking solution for 1 h. Secondary antibodies were added at a final concentration of 1:500, and brains were incubated overnight. Secondary antibodies were washed off in three quick washes in sterile filtered 1× PBS, and brains were incubated in DAPI (1:500 of a 1 mg ml^−1^ stock solution) in sterile filtered 1× PBS for 10 min, followed by three 20-min washes in sterile filtered 1× PBS. All staining steps were performed at room temperature on a benchtop rocker. Stained brains were mounted on silane-coated glass slides in SlowFade Glass mounting medium.

### Verification of antibody specificity

We used both antibody pre-adsorption experiments and dual RNA FISH + IHC staining experiments to verify the specificity of the AstA and NPF antibodies. The rabbit-anti-AstA antibody has been shown to bind to AstA neuropeptides that end in a terminal YXFGL-amide motif^103^. *O. biroi* AstA encodes five AstA neuropeptides, four of which end in a similar motif (AstA1–AstA4). Three of those are unique: AstA1 (YNFGL-amide), AstA2 (FSFGI-amide) and AstA4 (FSFGL-amide) (see Supplementary Table 3). For the AstA pre-adsorption control, rabbit-anti-AstA (1:2,000) was incubated with AstA1, AstA2 or AstA4 at a concentration of 1 mM in IHC blocking solution overnight at 4 °C. For the NPF pre-adsorption control, chicken-anti-NPF (1:1,000) was incubated with *O. biroi* NPF peptide at a concentration of 1 mM in IHC blocking solution overnight at 4 °C. Brains were then IHC stained with pre-adsorbed rabbit-anti-AstA solution or pre-adsorbed chicken-anti-NPF solution as the primary antibodies, or with non-pre-adsorbed rabbit-anti-AstA or non-pre-adsorbed chicken-anti-NPF as positive controls, respectively. All IHC staining steps for the pre-adsorption controls were performed as described above. For the dual RNA FISH + IHC experiments, brains were fixed and first processed for either AstA RNA FISH or NPF RNA FISH as described above, but at the last step brains were switched from 5× SSCT buffer into sterile filtered 1× PBS through three 20-min washes. Brains were then processed for AstA or NPF IHC staining, respectively, as described above.

### Quantitative confocal microscopy

All brain samples within an experiment to be imaged for quantitative confocal microscopy were dissected, fixed and stained in parallel. Stained brains were mounted on silane-coated slides in SlowFade Glass mounting medium, and coverslips were sealed with clear nail polish. Slides of mounted brains were allowed to clear at room temperature inside a dark drawer overnight before imaging. All confocal images were acquired with a Zeiss LSM 900 confocal microscope using a Zeiss LD LCI Plan-Apochromat 40×/1.2-NA multi-immersion objective lens and glycerol immersion medium. Confocal *z*-stacks of whole brains were captured in a single image plane at 0.5 zoom and 1-μm optical steps at 4,084 × 4,084-pixel resolution using Airyscan multiplex fast settings in Zen software. Airyscan postprocessing of raw images was done using standard strength settings in Zeiss Zen Blue software. All brains within an experiment were imaged using the same settings for laser power, gain and offset, such that fluorescence measures were comparable across conditions.

### Image analysis

Fluorescence was measured from confocal images using ImageJ. Both FISH and IHC staining of AstA had high signal-to-noise ratios, allowing thresholding to cleanly separate signal from background. To quantify regional differences in AstA FISH and IHC staining, ROIs were hand-drawn around the antennal lobes, protocerebrum (without mushroom bodies), SEZ, corpora allata and mushroom bodies. AstA signal was measured from every image slice in the *z*-stack. The total average fluorescence from each brain region was normalized by the total average fluorescence for the mushroom body (where AstA is not expressed) such that measurements were comparable between brains and across experimental conditions. Because NPF is expressed in only 16–20 cells per brain, we were able to hand-draw ROIs around the somas of every cell. The average NPF fluorescence was then measured from 3–12 image slices per cell for both FISH- and IHC-stained brains. NPF fluorescence measures were normalized to the total average brain fluorescence for each brain. Whole-brain NPF measures presented in the figures are the average of the normalized NPF fluorescence for the 16–20 cells per brain. Regional fluorescence measures for NPF are presented as averages of the normalized NPF fluorescence measures of subsets of cells within the antennal lobes, the adPC, the pdPC and the SEZ.

### Annotation of *NPF*

While annotating *NPF*, we found two loci with near perfect identity in different positions on chromosome 1 (chr. 1) in the current *O. biroi* genome assembly and annotation available at the National Center for Biotechnology Information (NCBI) (assembly Obir_v5.4, annotation release 100). These were pro-neuropeptide Y-like (LOC113562173; chr. 1: 6,886,485–6,893,071) and uncharacterized (LOC105286742; chr. 1: 1,022,113–1,028,788). LOC113562173 occurs on the positive strand, whereas LOC105286742 occurs on the negative strand, such that the two loci are reverse complements. Aligning these two approximately 6.5-kb gene models revealed that they are 99.9% identical, except for a total of 26 bp of 1–2-bp gaps spread throughout the two loci. These observations suggested that NPF might have undergone gene duplication in *O. biroi*. However, several lines of evidence argue that this ‘duplication’ is, in fact, an error introduced during genome assembly, rather than a true duplication. First, BLAST alignment of either LOC113562173 cDNA or LOC105286742 cDNA to a haploid male genome assembly recovered only the same single hit on chr. 1 (assembly generated using short-read whole-genome sequences assembled with SOAP denovo^104^ from published samples: BioSample SAMN33870437 from BioProject PRJNA947942)^28^. Next, we PCR-amplified *NPF* exon 1 from both genomic DNA and a library of brain-derived cDNA, followed by Sanger sequencing (primer sequences are in Supplementary Table 5). The resulting sequences were identical to those of the haploid male genome assembly and differed from Obir_v5.4 LOC113562173 exon 1 at the single 2-bp gap, showing no evidence of the ‘duplicated’ LOC105286742 in the Sanger sequence traces. However, attempts to amplify the entire (5′-untranslated region (UTR) to 3′-UTR) LOC113562173 from genomic DNA (gDNA) or to clone the *NPF* cDNA using primers based on LOC113562173 or LOC105286742 failed. Furthermore, alignment of gDNA and brain-derived RNA-sequencing reads to Obir_v5.4 revealed islands of coverage peppered with areas of zero coverage in both LOC113562173 and LOC105286742. Together, these observations suggest that there is only one *NPF* gene in *O. biroi*, that the duplicated loci in assembly Obir_v5.4 are due to assembly errors and that the *NPF* gene model might be partially incorrect.

To manually annotate the *NPF* gene model, we used several independent approaches. First, we aligned the *NPF* exon 1 sequence to a recently published diploid female genome assembled from high coverage PacBio long read sequences, Obir_v5.6 (ref. ^105^). Because this reference sequence was assembled using a diploid-aware assembler, it should be less prone to introducing false duplications into a haploid genome sequence. As expected, this revealed a single locus on chr. 1. Next, we generated candidate *NPF* transcripts by de-novo-assembling a published dataset of brain-derived RNA sequences (BioProject: PRJNA304722)^106^ using Trinity v.2.14.0 (ref. ^107^). We identified two candidate *NPF* transcripts, both of which aligned to the same location as *NPF* exon 1 on chr. 1 of Obir_v5.6. Finally, we aligned an independent set of brain-derived RNA-sequencing reads to Obir_v5.6 to add coverage data to support our annotation of the gene model. Together, these analyses revealed a 6,254-bp locus including 5′-UTR, two exons and 3′-UTR at position chr. 1: 5,408,614–5,414,868 in Obri_v5.6. Aligning Obir_v5.6 *NPF* to LOC113562173 revealed that the coding sequence, including the part encoding the NPF neuropeptide, is nearly identical, except for the 2-bp gap in exon 1 described above. However, the two versions have different sequences in the 5′-UTR beginning 88 bp upstream of the translational start site, explaining our failed attempts to clone the *NPF* cDNA using primers within the incorrect 5′-UTR sequence of LOC113562173. However, we successfully cloned the *NPF* cDNA using primers based on the Obir_v5.6 *NPF* sequence as described below (Supplementary Table 5). We expect that future NCBI reference assemblies and annotations for *O. biroi* will resolve the assembly errors and artificial duplication of the *NPF* locus in Obir_v5.4. However, given that the *NPF* coding sequence is mostly identical and that the *NPF* neuropeptide sequence is correct in both assemblies, we report the *NPF* locus as pro-neuropeptide Y-like (LOC113562173) as found in the currently available NCBI reference genome. On the basis of our analyses, the correct *NPF* cDNA sequence (Obir_v5.6) with which we designed our RNA FISH probes and antibody, is given below. The translation of this *NPF* cDNA, which highlights the NPF neuropeptide sequence, is reported in Supplementary Table 2.

>*NPF* cDNA (Obir_v5.6) 696 bp:

5′_GATCCGAACTACTGAAACGTTCGCCGGTACGTCAGGAAGATATACCAGAAAGCTGCCGACAGGTGAGGAAGAAATCGTCTATCAGACGGACTCGGATTGCTCTTAAGCCGATCTTCCCCCTTATTCGCGGTTTGCCATTCGATCTACGATCCGATCGTCAGGGAGATTTAATCCAACCGAGACTGCTACCTCAAGCCGTTCAAAATGCGGACAGAGATGAGTGCAGCTCGTTTTCTTTGTTGCTTACTGTTGATCGCCATTGTGGGAACTGTCATCGCATGTGCCGAGCCGGAATCCATGGCTAGATCGACGAGACCAAAAGTAATTGCTAATTCCGAAGAATTGAAGCGATATCTCGATCTTGTTCGAGAGTATTATTCCATGACAGGAACAGCAAGGTTTGGAAAGCGAAATGATCCACCAATCTCGGAGAATACTATTTGGGAAATATTTAGGATGATCCTGGACAATGCACAACGGCAAAATGAACAGCGAAGACGGGATAGGATCAGACAGAACAAAGAGCCAGTCCCGTTTAATGACTTCTAGAGTTTGGATGCTGACAAGTACACGTCGCTACACGACCTGGCTCTCATCCGAATGTAATTGACAAATACTTGGACAATGTACAATAAATTTCATAGCGTTTTCCCTTTTACTCTCCCTAATTTCCATTGAAATATATCAAAGATGTTA_3′.

### Cloning of *NPF*, *AstA* and *GFP* cDNAs

To clone *NPF* and *AstA* cDNAs, we prepared a general library of cDNAs from total RNA extracted from a sample of 100 pooled ant heads. In brief, heads were removed from live ants and placed in cold TRIzol reagent on ice and then flash-frozen on dry ice. The sample was homogenized using a QIAGEN TissueLyser II, centrifuged and transferred to a PhaseLock column (5PRIME) to extract the RNA. RNA was then purified using the QIAGEN RNeasy Mini Kit according to the manufacturer’s protocol, including on-column DNase treatment. RNA quality and concentration were assessed using a Bioanalyzer (Agilent). The extracted RNA was of high quality, with an RNA integrity number (RIN) higher than 8.0. cDNAs were then generated using the Superscript IV First Strand Synthesis System Kit (Invitrogen; 18091050) with oligo-dT primers. We designed primers tagged with T7 RNA polymerase transcription start sites for *NPF*, *AstA* and *GFP* (Supplementary Table 5). T7-NPF and T7-AstA cDNAs were PCR-amplified from the 100-head cDNA sample. T7-GFP was PCR-amplified from an NdeI-linearized plasmid containing a *D. melanogaster* codon-optimized *eGFP* gene (plasmid plM153; gift from R. Coleman). T7-NPF, T7-AstA and T7-GFP were then cloned into the pCR4-TOPO TA vector using the TOPO TA Cloning Kit for Sequencing according to the manufacturer’s protocol (Invitrogen, K457501). The resulting vectors (pCR4-T7-NPF, pCR4-T7-AstA and pCR4-T7-GFP) were transformed into competent *Escherichia coli* cells that were then streaked on penicillin BACTO Agar plates (BD, 214010) and incubated at 37 °C overnight. Colonies were selected and plasmid DNA was extracted using the QIAGEN Plasmid Mini Kit according to the manufacturer’s protocol (QIAGEN, 12123). Positive clones were identified by EcoRI restriction enzyme digest of plasmid DNA and gel electrophoresis analysis. Sequences were then confirmed by Sanger sequencing (GENEWIZ).

### Generation of long double-stranded RNAs for dsRNAi experiments

Long double-stranded *NPF*, *AstA* and *GFP* RNAs (dsRNAs) were generated using the MEGAscript RNAi Kit (Invitrogen, AM1626) according to the manufacturer’s protocol (Supplementary Table 6). In brief, we PCR-amplified T7-NPF, T7-AstA and T7-GFP from their respective pCR4-TOPO TA vectors and used 1 μg of each of these products as the template in a 4-h in vitro transcription reaction to generate dsRNA. The resulting dsRNA samples were then nuclease-digested with DNase and RNase to remove the T7-PCR templates and single-stranded RNAs. The samples were column-purified and eluted with two 50-μl doses of elution buffer preheated to 95 °C. The resulting dsRNAs were then precipitated by incubating with 3 volumes of ice-cold ethanol and 0.1 volumes of 3 M sodium acetate at −80 °C overnight. The samples were centrifuged, and the dsRNA pellets were washed three times in ice-cold ethanol, then dried at room temperature and resuspended to 10 μg μl^−1^ concentration in sterile filtered RNA Duplex Buffer (1× RDB: 30 mM HEPES and 100 mM potassium acetate, pH 7.5). The samples were then aliquoted and stored at −80 °C before use. Fresh aliquots of *NPF*, *AstA* and *GFP* dsRNAs were used for each experiment.

### Head injection protocol

Ants were anaesthetized on ice and then secured on their side on a small dab of adhesive removable poster putty (Duck Brand) under a dissection microscope, such that the lateral aspect of the head was in view. Forceps and a 25G syringe needle point (BD PrecisionGlide Needle; 305122) were used to gently open a small hole (less than 0.3 mm in diameter) in the cuticle on the lateral aspect of the ant head. Injection solutions were loaded into a quartz glass micropipette (Sutter Instrument; Q114-53-10NP) pulled needle connected to either a Nanoject-II microinjector (Drummond Scientific) or a FemtoJet 4i microinjector (Eppendorf) and mounted on a four-axis micromanipulator. Using the micromanipulator controls, the micropipette needle was gently inserted into the hole at an angle of around 30° to minimal depth to avoid damaging exposed mandible muscles that laterally surround the brain. Then, 10–14 nl of injection solutions were injected using slow speed on the Nanoject-II, or 300-hPa injection pressure (*p*_i_) and 0-hPa compensation pressure (*p*_c_) on the FemtoJet 4i. When using the FemtoJet 4i, the time to inject the desired volume was calibrated for each needle by continuously dispensing fluid from the needle for 30–90 s onto parafilm, then measuring the volume of the resulting drop of fluid (typically around 1 μl). After the injection, the needle was carefully pulled away from the head and ants were gently removed from the adhesive putty and placed into a Petri dish filled with water-dampened plaster of Paris. Ants were allowed to recover for a minimum of 2 h in the climate control room before subsequent experiments.

### dsRNAi injection experiments

dsRNAi injection solutions were prepared by mixing fresh aliquots of *NPF*, *AstA* or *GFP* dsRNAs in 1× RDB with sterile filtered artificial haemolymph (1× AHL: 103 mM NaCl, 3 mM KCl, 26 mM NaHCO_3_, 1 mM NaH_2_PO_4_, 8 mM trehalose dihydrate, 10 mM dextrose, 5 mM TES, 4 mM MgCl_2_ and 1.5 mM CaCl_2_; pH 7.3) and rhodamine B (Thermo Fisher Scientific; 296570100) to aid visual confirmation of injection. The components were mixed to final concentrations of 1 ng nl^−1^ dsRNA, 0.8× RDB, 0.2× AHL and 20 μM rhodamine B. Live, anaesthetized ants were injected with 13.2 nl of injection solutions into the lateral aspect of the head as described above. The ants were then grouped into colonies according to treatment condition together with fourth instar larvae in a 1:1 workers-to-larvae ratio. These colonies were fed and allowed to recover overnight. Then, at 24, 48 and 72 h after injection the surviving ants were tested in the brood-care behaviour assay. After the first two behavioural tests, the ants were returned to their colonies and fed again. In the *AstA* dsRNAi experiment, 66% of *AstA* dsRNAi and 75% of *GFP* dsRNAi ants were still alive at 72 h after injection. In the *NPF* dsRNAi experiment, 66% of *NPF* dsRNAi and 83% of *GFP* dsRNAi ants were alive at 72 h after injection. After the third behavioural test, we immediately dissected the brains from the ants and processed them for RNA FISH staining to measure *NPF* or *AstA* expression using quantitative confocal microscopy.

### siRNAi injection experiments and qPCR

We designed 27mer Dicer-substrate siRNAis targeting the coding sequences of *NPF*, *AstA* and *eGFP* (Supplementary Table 6) using the RNAi Design Tool provided by Integrated DNA Technologies (IDT). We resuspended the siRNAis in 1× RDB to 100 μM stock concentration and stored them at −20 °C before use. Injection solutions were prepared by mixing siRNAis with sterile filtered AHL and rhodamine B to final concentrations of 50 μM siRNAi, 0.5× RBD, 0.5× AHL and 10 μM rhodamine B. Live, anaesthetized ants were injected with 13.2 nl of injection solutions into the lateral aspect of the head as described above. The ants were then grouped into colonies according to treatment condition and grouped with fourth instar larvae in a 1:1 ratio. These colonies were allowed to recover for 72 h, during which they were fed daily. At 72 h after injection, we tested the surviving ants in the brood-care behaviour assay. In the *AstA* siRNAi experiment, 91% of *AstA* siRNAi and 86% of *GFP* siRNAi ants were alive at 72 h after injection. In the *NPF* siRNAi experiment, 83% of *NPF* siRNAi and 63% of *GFP* siRNAi ants were alive at 72 h after injection. Immediately after the behavioural assay, we removed the ant heads and processed them for qPCR to measure *NPF* and *AstA* expression. In brief, for each sample, heads were removed from two to four live ants, pooled, placed into cold TRIzol reagent on ice and flash-frozen on dry ice. For each of the siRNAi experiments, nine samples per treatment condition of control *GFP* siRNAi and either *AstA* siRNAi or *NPF* siRNAi were collected. The samples of ant heads in TRIzol were then homogenized using a QIAGEN TissueLyser II, centrifuged and transferred to PhaseLock columns (5PRIME) to extract the RNA. RNA was extracted and purified using the QIAGEN RNeasy Mini Kit according to the manufacturer’s protocol, including on-column DNase treatment. RNA quality and concentration were assessed using a Bioanalyzer (Agilent). RNA from all experiments was of high quality, with RIN > 8.0. cDNA was then generated using the SuperScript IV VILO Master Mix Kit with oligo-dT primers (Thermo Fisher Scientific; 11756050) and 30 ng RNA input per sample. Negative (no reverse transcriptase; RT−) controls for the cDNA generation were also made. qPCR primers specific to *AstA*, *NPF* and *GAPDH* were designed using NCBI Primer-BLAST to cross exon–exon junctions and with otherwise default settings. qPCR reactions were run using Applied Biosystems SYBR Green PCR Master Mix (Thermo Fisher Scientific, 4309155) with a 10-μl reaction volume on an Applied Biosystems QuantStudio3 thermocycler. We validated that the *AstA*, *NPF* and *GAPDH* primers (Supplementary Table 5) had higher than 90% efficiency using five tenfold serial dilutions of control cDNAs to generate standard curves before experiments. For the qPCR experiments, all samples were run in three technical replicates, including no-template and RT− controls. Average *C*_T_ values were calculated from the technical replicates with automatic outlier removal provided in the QuantStudio3 software. Expression was then analysed using the −ΔΔ*C*_T_ method^108^.

### Peptide injection experiments

NPF and AstA2 were synthesized by Bio-Synthesis to higher than 95% purity and were provided lyophilized. Lyophilized NPF and AstA were stored at −80 °C for up to 2 months before resuspension. Immediately before experiments, the lyophilized peptides were resuspended in pure DMSO to a stock concentration of 10 mM. Peptide injection solutions were prepared by mixing stock solutions of NPF and AstA peptides with 1× AHL and rhodamine B to aid in visual confirmation of injection. These components were mixed to final concentrations of 50 μM NPF or AstA peptide, 1× AHL, 10 μM rhodamine B and 0.1% DMSO. Control solutions were 1× AHL, 10 μM rhodamine B and 0.1% DMSO. Live, anaesthetized ants were then injected with 10 nl of injection solution into the lateral aspect of the head as described above. The ants were grouped into colonies according to treatment condition and allowed to recover for 2 h in the climate control room. In the NPF peptide injection experiment, all ants survived. In the AstA2 peptide injection experiment, 90% of AstA2-treated and 86% of control-treated ants survived. The surviving ants were then tested in the brood-care behaviour assay.

### Nutritional status modulation experiment

We prepared ants and larvae for this experiment from a naturally cycling colony in the foraging phase. We counted the day that the newest generation of young ants eclosed from pupae as day 0. We fed the colony on days 0 and 2 with *S. invicta* brood stained with bromophenol blue. Then, on day 4, two small colonies of 50 four-day-old ants with 50 larvae were separated into two 30-mm-diameter Petri dishes filled with a layer of moist plaster of Paris. One colony was fed four more times over the next 8 days (on days 5, 7, 9 and 11), whereas the other colony was starved during these 8 days. At the end of this period, all ants were 12 days old. We tracked larval survival and found that 48 out of 50 larvae and 49 out of 50 larvae survived in the fed and in the starved colony, respectively. All ants in both fed and starved conditions survived to 12 days of age. The 12-day-old fed and starved ants were then tested in the brood-care behaviour assay. Immediately after the behaviour assay, their brains were dissected and processed for RNA FISH staining to measure *NPF* and *AstA* expression using quantitative confocal microscopy as described above.

### Reporting summary

Further information on research design is available in the Nature Portfolio Reporting Summary linked to this article.

## Online content

Any methods, additional references, Nature Portfolio reporting summaries, source data, extended data, supplementary information, acknowledgements, peer review information; details of author contributions and competing interests; and statements of data and code availability are available at 10.1038/s41586-026-10747-6.

## Supplementary information

Reporting Summary

Supplementary Table 1 *O. biroi* neuropeptide precursor gene homology. This table lists the identified neuropeptide precursor genes in *O. biroi* and a representative species and neuropeptide precursor gene ortholog used in the homology search to annotate the *O. biroi* neuropeptidome.

Supplementary Table 2 Translated and annotated *O. biroi* neuropeptide precursor gene sequences. This table lists the *O. biroi* precursor protein sequences annotated with the signal peptide and neuropeptide(s).

Supplementary Table 3 *O. biroi* neuropeptidome. This table lists the *O. biroi* neuropeptide names and their sequences, along with their precursor gene, NCBI protein accession number, and whether we tested them in the primary screen.

Supplementary Table 4 Primary neuropeptide screen results. This table lists the *P* values and effect sizes (as Cohen’s *d*) for the neuropeptides that had some significant effect in the primary neuropeptide screen. The ranks of the neuropeptides are shown and those selected for the secondary round of screening are noted.

Supplementary Table 5 Primers used. This table lists the primer sequences used in this study and what they were used for, as well as their Tm values and amplicon sizes.

Supplementary Table 6 Sequences used for dsRNA and siRNA experiments. This table lists the sequences of the cloned AstA, NPF, and GFP fragments used for generating the dsRNA and the siRNA duplex complexes.

Supplementary Video 1 The brood-care behaviour assay. Top frame: representative video of a 2-month old ant paired with a 4th instar larva in the brood-care assay. Middle frame: animated representation of tracking blobs for the adult ant (blue), the larva (orange) and when the adult and larva are physically interacting (magenta). Bottom frame: animated position trace (*y* axis) over time (*x* axis) of the ant (blue), larva (orange) and when they are interacting (magenta). The video is played back at 8× speed and jumps forward in time (clock h:m:s; green, lower left corner) to show relevant phases of the behaviour. At the start of the movie, 00:10:50, the ant has awoken from anaesthesia and initiated a local search of the assay chamber. The ant first detects the larva at 01:33:30. From this point, phase P1 lasts until about 02:24:00 when the ant begins to transition to phase P2. Phase P2 then persists to the end of the recording.

Supplementary Video 2 *NPF* and *AstA* RNA FISH-stained brains. Representative brains from 12-day-old ants FISH-stained for *NPF* RNA (left) or *AstA* RNA (right). For both brains, RNA is shown in green, and nuclei (DAPI) are shown in blue. Brains are shown as rotating 3D projections to visualize the distribution of *NPF*^+^ and *AstA*^+^ cells around the brain. The initial frame in the movie shows the dorsal surface of the brains, with anterior at the bottom and posterior at the top. Note that the ventral side of the NPF-stained brain (left) is not shown because no *NPF*^+^ cells are present in that region.

Supplementary Video 3 NPF and AstA IHC-stained brains. Representative brains from 12-day-old ants IHC stained for NPF peptide (left) or AstA peptide (right). For both brains, peptide is shown in red, and nuclei (DAPI) are shown in blue. Brains are shown as rotating 3D projections to visualize the localization of NPF^+^ and AstA^+^ cells and neuropil throughout the brain. The initial frame in the movie shows the dorsal surface of the brains, with anterior at the bottom and posterior at the top. Note that the ventral side of the NPF-stained brain (left) is not shown because no NPF^+^ cells or neuropil are present in that region.

Peer Review File

## Source data

Source Data Fig. 1

Source Data Fig. 2 and Extended Data Figs. 1 and 2

Source Data Fig. 3 and Extended Data Fig. 4

Source Data Fig. 4 and Extended Data Figs. 5, 6 and 8

Source Data Fig. 5 and Extended Data Figs. 9 and 10

Source Data Fig. 6 and Extended Data Fig. 11

## Data Availability

All data are included with the publication as Supplementary Information. Source data are provided with this paper.

## Code availability

Code used for behavioural analysis and sample datasets is available via GitHub (https://github.com/Social-Evolution-and-Behavior/Paul_Kay_Kronauer_2026).
