# Supplementary material for: Ancient feeding-related neuropeptides regulate alloparenting in ants
Source: Nature. 2026 Jul 8;656(8129):948–56. doi: 10.1038/s41586-026-10747-6 (PMC13518240; doi:10.1038/s41586-026-10747-6)
Supplement: Supplementary file 1 [file 41586_2026_10747_MOESM11_ESM.pdf]

nature portfolio

# Peer Review File

## Ancient feeding-related neuropeptides regulate alloparenting in ants

Corresponding Author: Professor Daniel Kronauer

Any redactions in this file are there to maintain patient confidentiality, the confidentiality of unpublished data, or to remove third-party material.

**This file contains all reviewer reports in order by version, followed by all author rebuttals in order by version.**

**Attachments originally included by the reviewers as part of their assessment can be found at the end of this file.**

Version 0:

Reviewer comments:

Referee #1

(Remarks to the Author)
Review of Paul et al. Two feeding-related neuropeptides, NPF and AstA, regulate brood care behavior in the clonal raider ant.

The Kronauer lab has led social insect molecular neuroscience, including the development of CRISPR for application in insect sociobiology, using the clonal raider ant model. This is an advantageous model system due to genetic homogeneity and availability of molecular tools enabling the experimental alteration of gene expression. This molecular dissection of social behavior is step-by-step increasing our understanding of the mechanisms underlying sociality. The present submission represents potential for further contribution to this literature.

In this manuscript, the authors describe a series of detailed molecular analyses and experiments to investigate the role of neuropeptides in an aspect of potentially age-related behavior – care of larvae – in clonal raider ants. Starting from a large set of candidates, neuropeptides were synthesized and evaluated for potential influences on brood care. In a preliminary screening using less-pure neuropeptides, candidates were evaluated for their influence on worker/brood interactions by soaking individual workers in solutions containing each candidate molecule and then allowing treated workers to interact with larvae. In the behavioral assay, the time spent in contact with larvae was quantified using automated tracking; carrying larvae was also recorded. After selecting half of the neuropeptide candidates that showed the greatest effects on the type of interaction with larvae recorded in the assay, highly purified samples of the subset of neuropeptides were synthesized. Larval-care assays then focused on two neuropeptides: NPF and AstA2.
The authors measured change in expression levels of these two neuropeptides in young or old workers in specific areas of the brain using immunohistochemistry to quantify RNA and protein levels based on signal from fluorescent tags. This showed expression of NPF in the dorso-lateral protocerebrum and antennal lobes and expression of AstA2 throughout the brain. Increased worker age was associated with decreased NPF expression in four specific cells, with little change elsewhere and increased AstA2 only in the antennal lobes, with decreases or little change elsewhere. Levels of these neuropeptides were then manipulated using RNAi to decrease each in turn and by injecting each directly into the heads of ants to increase their titers.
The study is impressive in molecular detail. The conceptual framing and generalizability of the results and the validity of the larval care assay to the conclusions drawn, however, are problematic, as noted in “comments” below.
Comments:
Line 1 and throughout the manuscript: “brood-care behavior”
The title could have more impact reflecting the novelty and significance of the work if it stated that two ancestral regulators of feeding underpin age-related alloparental care in the clonal raider ant. Still, issues related to feeding per se need to be clarified, as noted below.

Line 17: This is the first reference to age-related division of labor being ubiquitous in social insects. Age-related worker behavior is presented as the simplified classical concept of temporal polyethism. While it may be common among the

relatively small number of ant species studied, it is not universal and it is inaccurate to imply that all social insect workers follow this pattern of behavioral development. Some basal ant species have evolved patterns of age-related behavior in association with variation in life history, colony age demography, and the coupling of foraging with the provisioning of larvae. Other social insect species show plasticity in age/task relationships that may arise from experimental demographic alterations. The characterization of newly eclosed workers as discretized nursing “specialists” originally proposed by Wilson is not a generality. Related concepts include response thresholds (mechanisms and evolution) and self-organization of age-related behavior (the “foraging for work” model).

The generalizability of results of the starved/fed and young/old worker treatments as drivers of interaction with brood across all ants is not convincing. Some workers receive nourishment by interacting with brood, while others forage to provide food to larvae. Some specialized workers seldom interact with brood. This diversity of types of age-associated worker behavior and its relationship to the study is important to consider.

Here and on line 37 and elsewhere it is therefore appropriate to refer to “age-related behavior” rather than “age polyethism” and to acknowledge the variation documented in the association of age and task performance, and related theories and concepts. With respect to this, Whitfield et al. 2003 should be cited, as it is a key paper on gene expression, behavioral development, and task transitions in honeybees.
Whitfield et al. (2006). Genomic dissection of behavioral maturation in the honey bee. PNAS 103, 16068316075

Additionally, physical caste evolution, like age-related behavior, should be noted in the context of the genetics/epigenetics of division of labor. Clonal raider ants are largely monomorphic, but other species of ants and other social insects produce specialized worker phenotypes, e.g., major workers or “soldiers,” with head structure and mandibles sometimes modified in extreme ways for phragmosis or defense. These workers are task-specialized, likely throughout their lifespan. There is a significant literature on the genetics and epigenetics of task switching in physical caste division of labor that should be recognized and discussed. Task acceleration and reversion are important processes of plasticity in worker behavioral development. See for example:
Lucas, C., & Ben-Shahar, Y. (2021). The foraging gene as a modulator of
division of labour in social insects. Journal of Neurogenetics, 35, 1683178.

Lucas, C., & Sokolowski, M. B. (2009). Molecular basis for changes in
behavioral state in ant social behaviors. PNAS, 106, 635136356.
Simola, D. F., et al. (2013). Social insect genomes exhibit dramatic evolution in gene composition and regulation while preserving regulatory features linked to sociality. Genome Research, 23, 123531247.
Simola D.F. et al. 2016. Epigenetic (re)programming of caste-specific behavior in the ant Camponotus floridanus. Science 351: 42
Line 51: Regarding the brood-care assay, was the enclosure narrow and linear as seen in the corresponding figure or are the linear sections just cropped to illustrate the distance between the worker and the brood?
Line 56: Here and in other places, detail is provided later on, but clarity would be enhanced by briefly describing how a numerical threshold was used to differentiate “bouts of exploration” from periods of “direct physical contact”.
Line 76: Please further clarify how you tested the ability of candidate molecules to penetrate the cuticle. This is alluded to but a description of how this was tested seems missing, or at least hard to locate.
Line 77: Similarly, here briefly summarize the method used for protein synthesis even though it is explained in detail later.
Line 133: Is there any reason to believe that NPF and AstA2 interact with each other, given that they have somewhat opposite effects? Is it more likely that their expression is independently regulated?
Line 138: Again, while this is described elsewhere, briefly state that injections were made through the lateral aspect of the head.
Line 167: Consider adding in discussion of workers receiving food from larvae, e.g., larval hemolymph feeding, which may not be a factor in clonal raider ants but would be in others, or digestion and regurgitation of solid foods by larvae to feed to workers. The reason for this is that it should be considered whether workers would be satiated after foraging in all cases.
Line 249: Does cleaning a porous ant nest floor with bleach while the colony is inhabiting the lab nest really not harm workers or alter their behavior or physiology? Chlorine dioxide is a fumigant pesticide known to produce oxidative stress in insects and mortality in ants. Mold inhibitors like Tegocept are non-toxic antimicrobial agents that could serve the same purpose.
Line 293: Does this indicate that NPF “passed” the first screening in that it had an upper 50% level of significance of effect on changes in brood interaction behavior, but that in the second round its effect was significant but in the opposite direction? This needs to be explained more clearly and the authors should include a comment on the impurities in the 92% pure synthesized compound that could have such a dramatic effect.
Line 306: Are the gel results or sequencing results included in a supplement?
Lines 376-381: The P1 behavioral phase is defined as “the period beginning with larva detection and continuing while the ant remains mostly in physical contact with the larva,” independent of carrying the larva around or remaining in one location. “Larva carrying did not consistently occur across all assays or conditions, and it was therefore collapsed into the P1 phase.” Do the authors rate larval transport higher than remaining in one position? What was the frequency of carrying larvae under control and experimental manipulations? Were grooming or feeding larvae recorded? Is there evidence of trophallactic transfer of crop or salivary gland complex secretions to larvae, perhaps based on the stain incorporated into the food?
The P2 phase “was defined as the period when the ant begins to leave the larva and subsequently spends most of its time exploring the assay chamber away from the larva.” Time durations of the phases were also recorded.
Quoting the Abstract, the main findings are:

1. NPF increases the tendency of ants to nurse their larvae

2. AstA promotes exploration away from larvae

A major issue is that the assay seems to mainly record olfactory recognition of larvae, an element of brood care, but not nursing, either in respect to feeding, which is a key behavior in the study, or grooming. Perhaps workers in contact with or carrying larvae are moving them to new locations based on microclimatic variation in the artificial nest. Perhaps workers are in the process of evacuating brood. What was the change in larval location at the end of the assay? These are brood-care behaviors that are not necessarily coupled with nutrition, and the present study is focused on neuropeptides having conserved functions in feeding. Trophallaxis (and grooming, although still not necessarily directly related to nutrition) would be more compelling metrics of nursing. Unfortunately, it appears that current automated tracking can identify worker/worker trophallaxis, but not worker/larval food exchange, at least in honeybees, which feed larvae in cells, thus obscuring the behavior.

Apparently, in the present study, workers did not groom or feed larvae, and responsiveness to larval “begging” behavior was not observed or recorded. Responsiveness to brood odors could be quantified in a simple olfactometer and negative or positive phototaxis, which could be associated with brood care or foraging (or at least nest departure), respectively, could easily be recorded. “Exploring” is undirected movement in an assay chamber with atypical dimensions and structure relative to a natural nest. “Exploring” is also behavior that is not described by its consequence, its function cannot therefore be identified with certainty. It would be speculative to refer to movement away from larvae as “foraging” or any behavior related to nutrition. To examine the effect of nutrition on brood-care behavior, starved and fed ants were tested in the above assay. “Starved ants spent significantly more time with larvae than fed ants.” However, this is positional data that although relevant to brood care does not establish a feeding relationship.

Lines 408-413: A statistician might be consulted to comment on this “permissive” test.
Line 743: Behav. Ecol. Sociobiol.
Other comments on references:

In referencing the genes/hormones/circuits approach, older Robinson lab studies could be updated by:

Sinha et al. (2020). Behavior- related gene regulatory networks: A new level of organization in the brain. PNAS 117, 23270323279.

In summary, this is an interesting study that could improve its conceptual framing and provide more compelling evidence of the role of neuropeptides in nursing behavior in the clonal raider ant as well as ants in general.

Referee #2

(Remarks to the Author)
This is an impressively complete and detailed examination of how specific neuropeptides, generally known to function in feeding behavior, regulate alloparental care in a clonal ant. The work is detailed, the evidence compelling and complete, and represents a major advance in our understanding of how social behavior might be regulated in social insects.

The experimental design is clever and especially so working on a non-model organism. The full toolbox of genetic techniques are not the same as you would have for genetic model organisms such as Drosophila, yet the authors provide multiple lines of evidence that go well beyond correlation and association to bolster their interpretations. The manipulations are important and appropriate, and the range of approaches and their analysis is well conceived and executed.

I have one minor question over the setup of the study. While the authors say "why neuropeptides", I think they can be clearer that pleiotropy is expected in specific neuropeptides rather than novel genes. There has been a number of studies that have suggested this hypothesis. The novelty and potential universality of the work would then be clearer.

Minor comments:

92 – note that NPF is homologus to NPY in vertebrates with the same association with feeding behavior. AstA receptor is homologus to Galanin receptor. Thus, I think you can expand the potential relevance of your work.
230 – finally get the information on ubiquity of genes studied. Could be made earlier.
Excellent study that carefully documents role of NPF and AstA in alloparental care. The work is unique in being complete – both manipulative studies, identification of brain regions as well as associations with behavior. The work is elegant and compelling.
My main concern is a lack of interpretation of the importance of the work. It somehow feels very specific to ants, but I think there is a greater global relevance. This could be made clearer if we were told about other studies of NPF and AstA and parenting – e.g., do these show up in trascriptomic association studies? I believe NPF has been associated with parenting, but I am less sure of AstA. What about NPY and GAL?
Perahaps part of the problem is a very ant-centric focus. Why is “alloparenting” important – is this just a form of care involving siblings or is it somehow different than parental care? I realize that these ants only have alloparental care (and not parental care), but for those of us who don’t study ants does this matter at all? What is the global importance – is it just for ants and perhaps other eusocial insects or does it imply relevance beyond eusocial insects and even perhaps insects?
I think the paragraph from lines 230-239 is very important (note my comment above when I first read line 92-) but we need

something in the introduction suggesting this is coming. Did you predict that you would see a co-option of feeding related neuropeptides? Others certainly have. Perhaps this can be highlighted in a sentence in the introduction.

Referee #3

(Remarks to the Author)
Summary: The authors developed an automated behavioural assay to monitor ant attentiveness to larvae and used it to screen 70 neuropeptides (exposed by submerging ants in a solution of the peptide). They found several potential candidates and explored two, one promoting nurse-like behaviour and one foraging. Through careful testing, with RNAi manipulations, neuropeptide supplementation and immunohistochemistry, they show that these change with ant age and feeding status, potentially underlying age-related polyethism.

Overall, they do a wonderful and thorough job of showing something that isn't terribly surprising, that neuropeptides regulate hunger and satiety in ants. Neuropeptides are well conserved. They've been studied in many insects. As the authors say, these ones are known to regulate feeding.

They do not however discuss much about how this work interrelates with their own work and others' on other neuropeptides involved in social life, caste behaviors and foraging. Also some relevant citations are missing/not discussed: e.g. https://doi.org/10.1002/cne.24109, https://doi.org/10.1371/journal.pone.0109590

The most interesting result in my mind is that starvation induced these ants to stay longer with brood. This is quite opposite to what was shown with another very different clonal ant: https://doi.org/10.1242/jeb.219238 (your ref 35). It leads to many more questions: Is cannibalism observed? Is this some more interesting decoupling of nutritional state from behavioural outcome via the neuropeptide signal? Fundamentally, is the clonal raider ant the odd one out here? And if so, why would that be?

Otherwise, I had a handful of issues and comments:
Figure 2: The notable differences in control (0 treatment) conditions between corresponding panels (b/f, c/g, d/h, e/i) require explanation. This inconsistency potentially impacts the interpretation of treatment effects and requires experimental design clarification.
L45-47: The perspective presented aligns with current understanding in the field, so not a particularly novel hypothesis.
The choice of the clonal raider ant as a model system presents certain limitations for studying age-related polyethism. Given their cycling behavior, the point at which they might exhibit age-related polyethism seems narrow and the dynamic range in the data seems to reflect that. It seems like a caveat that the reader should be aware of. Nearly all other ants have a much stronger shift between states. It might be worth showing that you can recapitulate these neuropeptide supplementation results in a more normal ant.
Including citations for the referenced work at L69 would enhance the paper's scholarly context and provide appropriate recognition of prior contributions to this research area. Also you have a lost citation on L71.
The soaking method. How do you think the neuropeptides get through the cuticle? Seems awfully risky to be so porous… Ingestion? And then to the brain??
Thank you (on behalf of future researchers) for providing the LOC numbers and XP numbers throughout!
L95-98: Can you clarify the differences here and what was inconsistent and why?
The manuscript would benefit from a clear explanation of the complementary roles of siRNAi and dsRNAi in your experimental design. Additionally, a rationale for emphasizing the siRNAi results over what seem to me as more robust dsRNAi data would help readers fully appreciate your methodological choices.
L185~ How does all of this actually relate to larval care? From the videos and descriptions, it doesn't sound as though they are actually doing much caring for the brood. Most other ants feed their brood orally, with prey or with trophic eggs – what are these ants doing with them during the brood care phase? It is just that they notice a larva and then look around fro something to feed it? Is this why they never come back to the larva?
The paragraph on the interplay with nutrition, JH, insulin, and these neuropeptide is nice, though I feel that the difference with the clonal raider ant results and other ants and solitary insects could be explored more clearly.
L288. It is hard to see how one can conclude much about something applied at 15% purity.
While power analyses were performed, the sample sizes (n=12 for behavioral assays and n=5 for brain analyses) appear insufficient given the data variability (e.g. Fig 3). Increasing sample sizes would strengthen the robustness of the findings. Figure 5 has larger n and is much more compelling.
Thank you for the Annotation of NPF section.

Referee #4

(Remarks to the Author)
This study presents intriguing results, obtained using an impressive variety of methods, on the role of two neuropeptides in interactions of workers and larvae in the clonal raider ant.

The main result in Fig 1g is that a young ant, when placed in a narrow track with a larva, spends more time about 3 mm closer to the larva than an older ant. The behavioral assay consisted of placing a single ant in a 30 by 2mm track with a

larva. The ant has to pass the larva as it goes back and forth along the track. The data were based on a measure by image analysis of how aligned were the bodies of the worker and larva, apparently indicating that the worker held the larva in its mandibles. The ant could have been inspecting the larva as a possible food item, or even moving it out of the way. In any case, proximity to a larva is not necessarily nursing, or 'brood care' as it is called in Figure 1. Brood care, or nursing, is a social activity performed by groups of ants in response to stimuli from groups of larvae (e.g. see Cassill's pioneering work on interactions between workers and larvae that stimulate feeding, and subsequent work by Dussutour). In some species, particular workers prefer to care for larvae at specific developmental stages (e.g. Pharaoh ant Warner et al. PLoS Genet 2019); if that occurs in clonal raider ants, the choice of worker may influence the results. It is also dubious whether an ant moving back and forth within a narrow track where it is confined could be called 'exploring'. The ant has no choice but to move along the track.

The results of this study may be more related to feeding behavior than to brood care. NPF and AstA regulate feeding other animals. Previous work by these authors showed that association with larvae is related to starvation. The results here on gene expression further support that: NPF levels increased in the WB, adPC, and AL of starved ants, while AstA levels increased in the WB, PC, AL, and SEZ of fed ants. Perhaps the young ants, who were taken from a brood chamber in the nest for the behavioral assay, were familiar with the smell of a larva, so spent slightly more time near it as a possible food source, while the older ants, who were likely to have been foragers in the nest they were taken from, (although this is not explained in the methods) were more likely to recognize that the conspecific larva was not the S. invicta larva they were usually fed, and to continue past it when going back and forth in the tube.

Thus the interpretation of the results far outreaches the behavioral data. Proximity of a lone worker and a larva in a narrow track, which may be related to feeding behavior rather than brood care, becomes 'affinity to larvae' which then becomes a 'nurse-like behavioral state', which then leads to the conclusion that the two neuropeptides 'regulate brood care' which then becomes an 'evolutionarily conserved' mechanism that regulates division of labor.

Regarding the results, aside from how they are interpreted: Lines 95-99 describe a result in the secondary screen that was inconsistent with the primary screen, as shown in Fig 2a and Extended Data Fig 4g. Fig. 2b–e shows that NPF increases P1 interaction time, while Extended Data Fig. 4h–k, shows that NPF increases P1 duration and total interaction time, but not P1 interaction time. The authors point out this inconsistency but do not explain why they decided to accept one result and reject the other.

To investigate how NPF and AstA regulate behavioral transitions, the authors manipulated the levels of both neuropeptides in the brains of young ants. To increase NPF and AstA levels, they injected synthetic neuropeptides; to decrease their levels, they used both dsRNAi and siRNAi. Both RNAi approaches produced consistent results. These manipulations were performed only in young ants, while the interpretation seems to suggest effects on older workers.

The authors suggest that the ratio of NPF and AstA regulates age polyethism (line 132). This interpretation requires further evidence. As shown in Figure 2, AstA is broadly expressed in the brain, with notable changes in the AL, SEZ, and CA. In contrast, NPF changes were limited to just four cells in the adPC. The authors proposed that an age-related shift in the NPF-to-AstA ratio underlies age polyethism. Because NPF and AstA are altered with age in distinct brain regions, and AstA shows a much broader distribution than NPF, further work is needed to explain where in the brain the effects of the two neuropeptides would act, or where the ratio between the amount of NPF in the adPC and the amount of AstA would be evaluated.

The general hypothesis presented here, that alloparental care is the result of the modulation of neuropeptides, is a form of the 'reproductive groundplan' hypothesis introduced by Mary Jane West-Eberhard in Developmental Plasticity and Evolution (2003), suggesting that the evolution of sociality is based on the modulation of physiological triggers for feeding larvae. It is odd that this body of work is never mentioned.

Referee #5

(Remarks to the Author)
I co-reviewed this manuscript with one of the reviewers who provided the listed reports.

Referee #6

(Remarks to the Author)
I co-reviewed this manuscript with one of the reviewers who provided the listed reports.

Version 1:

Reviewer comments:

Referee #1

(Remarks to the Author)
The authors have responded to the issues I raised in my first review; I appreciate their effort. However, a key concern remains.

It is important to completely understand the nature of brood care in order to confirm the validity of the lab brood-care assay, and thus the role played by neuropeptides. I read authors' recent publications to better understand the natural context of brood care in clonal raider ants and compared this to the design of their brood-care assay.
First, Snir et al. (2022) describe the role of pupal milk and provisioning with prey in nutritional nursing:

"Ant pupae extrude a secretion derived from the moulting fluid that is rich in nutrients, hormones and neuroactive substances. This secretion elicits parental care behaviour and is rapidly removed and consumed by the adults. This behaviour is crucial for pupal survival; if the secretion is not removed, pupae develop fungal infections and die. Analogous to mammalian milk, the secretion is also an important source of early larval nutrition, and young larvae exhibit stunted growth and decreased survival without access to the fluid." Furthermore, "social fluid contains a variety of micro- and macronutrients" and thus has nutritional value.

And:
"This fluid is secreted in large volumes by all pupae during a specific window of development, and elicits parental (sic) care behaviour that is necessary for pupal survival." From Fig. 3 in this article, "Pupae secrete their moulting fluid and adults place larvae on pupae. Larvae and adults consume the fluid, and fluid consumption prevents pupal infections and death." Therefore, pupal milk removal is an important, perhaps critical, element of brood care.
Second, concerning direct provisioning with prey, "Larvae have limited mobility and are thus dependent on adults to place them on food sources. Observations suggest that O. biroi adults readily place young larvae on pupae and older larvae on prey items."
Snir et al (2022) describe the complexity of brood care in clonal raiding ants. Therefore, I hope the authors can elaborate on how their relatively simple assay relates to this complexity. It seems that assays could have been developed that directly test elements of brood care that have functional assessments.
The authors state that such issues are addressed by the information presented in Figure 1. Nevertheless, brood-care behavior is characterized here as larval transport, larval manipulation, and larval "guarding" (what are they "guarding" against"?). The authors note in the response letter that "in the colony they also place larvae on food, but O. biroi adults do not feed larvae via trophallaxis."

Although I understand assays may have to be simplified to assess complex behavior, it is unclear why what would appear to be the most significant components of larval care are not reflected in the present design. The authors note "…a stereotypical two-phase response to larvae. In the first phase (P1), ants spend most of their time in direct physical contact with the larva. In the second phase (P2), ants tend to leave the larva for short bouts as they move around the corridor". "Transporting" and "manipulating" concern brood care in a general sense, and perhaps captures a portion of age-related alloparenting, but brood-care behavior is much more involved in clonal raider ants. I assume worker responsiveness to pupal milk can be measured, and prey could be provided to record their placement on larvae. These evaluations are ethologically robust and accurate reflections of brood care in this species.

Referee #2

(Remarks to the Author)
This is exciting work that demonstrates that NPF and AstA are coopted from an ancestral function in self-feeding to influence alloparental care. The novelty is threefold: first, this is the most complete examination, incorporating developmental changes as well as behavioral changes because of the unique system they have for study; second, the work is pleasingly complete with multiple avenues of investigation and experimental support; and third, this work shows that parenting in general, from mice to fish to eusocial insects, involves the cooption of neuropeptides with an ancestral role in feeding.

The authors have adequately addressed all of the previous concerns, with a much more robust demonstration of how their assay reflects alloparental care. Studies of behavioral genetics typically rely on an assay to reflect what can be a complex behavioral category (such as parenting) and I find that their assay is as good as any I've seen.

The approach here is pleasingly complete, with multiple lines of experimental evidence used to support their interpretations. The data are appropriately analyzed and interpreted.

The discussion of this paper is excellent - understated without obfuscation and complete without verbosity. This is a paper I expect to have a major impact on how we view the evolution of parental care, and to have an outsized effect on how others approach the mechanistic underpinnings of behavior as plastic and complex as parenting.

Referee #4

(Remarks to the Author)
The revised manuscript is much improved. The revision is thorough and extensive, and the authors responded to most of the concerns raised in the review. Most important, they added a behavioral assay to show that larvae are not treated as food.

There are two other points that could be clarified in the final version. First, about the contradictory results from the two peptide screens: The authors explain this by saying that round 1 was a rough screen to narrow down the candidates, and round 2 is more reliable, suggesting that the round 1 signal could reflect variation due to noise. That could be true, but it doesn't really resolve the contradiction or build confidence in the screen itself. If round 1 can produce signals that later flip or

disappear by chance, then what justifies using it to narrow candidates? In this version the authors are asking the reader to trust round 2, and discount round 1, without showing why round 1 was informative at all rather than just unreliable.

Second, about manipulating only NPF and AstA in young workers. This would be sufficient if their goal is explicitly developmental, asking how these peptides regulate the nurse to forager transition. However, they don't say that in their response. Instead, they just argue that testing young workers is sufficient for their conclusions, without directly acknowledging the alternative possibility that peptide effects in older workers could differ in direction or be reversible. It would be good to state explicitly that they are testing only developmental function during the transition period, not reversible or state-dependent effects in foragers.

Referee #5

(Remarks to the Author)
I co-reviewed this manuscript with one of the reviewers who provided the listed reports.

Version 2:

Reviewer comments:

Referee #1

(Remarks to the Author)
I've reviewed the authors' response to my brood-care assay concerns and have read the revised submission. Unfortunately, the difficulty I have with the design of the assay and interpretation of results remains. I admire the authors' work and hope my comments will result in a qualification of the assay and interpretation of results in the paper.

The nursing assay (edited for brevity without changing content):
individual ants are placed into a narrow corridor along with a single larva and are filmed for 5 hours. As in colonies, young ants spend twice as much time in contact with larvae as old ants.

Comment: I may have missed it – where is this data presented?

Ants also exhibit the same nursing behaviors towards larvae in this assay as in colonies. Importantly, if presented with a food item instead of a brood item in this assay, both young and old ants exhibit qualitatively different behaviors, and the age-linked behavioral differences disappear. Finally, ants never consumed or killed larvae in this assay. Together, this shows that our behavioral assay captures core aspects of alloparental behavior, rather than feeding behavior.

Comment: Isn't feeding behavior part of larval care (see Snir et al. 2016, below, red highlight).

Ants display a stereotypical two-phase response to larvae. In the first phase (P1), ants spend most of their time in direct physical contact with the larva. In the second phase (P2), ants tend to leave the larva for short bouts as they move around the corridor.

Comment: how is leaving larvae for short bouts an element of brood care"

This two-phase response is specific toward larvae and depends on hexane-soluble larval compounds, which are often conspecific recognition cues in ants.

Author response (italics): The important point here is that an ant's tendency to perform all nursing-related tasks within a natural colony are highly correlated. They are correlated because most ant workers are either nurses or foragers.

Is there data (covariance analysis and/or transition probabilities) to demonstrate the association of nursing acts, or are the authors referring to this broadly for social insects, without specificity for the present case?

Authors response: The elegance of our assay lies in the fact that it allows us to evaluate the ants' position on the nurse to forager spectrum by quantifying well-established aspects of nurse and forager behavior. However, it is evidently impossible (and unnecessary) to measure all aspects of the behavioral differences between these two states. This is what Referee #2 alludes to when they write that "Studies of behavioral genetics typically rely on an assay to reflect what can be a complex behavioral category (such as parenting) and I find that their assay is as good as any I've seen."

Authors refer to Reviewer 2's appraisal of the brood-care assay; I'm hoping that they will squarely address the issues I raised. I respectfully do not agree that the assay is "elegant" or "the best I've ever seen." There are for example straightforward assays of olfactory responses to brood as well as demonstrations of the effect of nursing and its effects on larval growth, as reported in Snir et al. 2016 and in other papers on ants. The frequencies of the most significant nursing behaviors can be quantified.

Authors response: Figure 1 shows that the behaviors captured in our assay constitute core aspects of brood care and that our assay serves as a reliable and quantifiable proxy of nurse-like behavior.

The behaviors in the authors' assay record worker position, which is difficult to assess nursing function. Proximity is a useful but very general metric. Behaviors with measureable effects are better. Quantifying the most significant acts of nursing (see Snir et al.) provides convincing data.

Authors response: Ants frequently exhibit guarding behavior in laboratory colonies – naturally these workers would be the front-line if an intruder entered the nest

My point is that unless a quantifiable response to a stimulus is recorded, a stationary position cannot conclusively be considered "guarding" unless this interpretation of position is validated by other data, for example, data demonstrating that these ants are in fact "first responders" to a threat. The alternative is that they might actually flee and other workers provide defense. If a defensive contingency is presented, and workers indeed show defensive behavior, this result would be convincing, but in the absence of such data, the function of the "guarding" behavior is inconclusive and essentially has the same problem as any proximity metric. One could interpret such "guarding" behavior as simply inactivity, and I did not find data to refute this hypothesis.

Snir et al. 2016 present compelling descriptions of the fitness consequences of nursing that should form the basis of brood-care assays.

Quoting Snir et al. 2016, "In ants, for example, adults groom, transport and feed the larvae. One important type of social interaction is trophallaxis, the sharing of liquid food in the form of 'social fluids' either mouth-to-mouth or anus-to-mouth. Trophallaxis is widespread among eusocial insects and it is common in many ant species, both between adults and between adults and larvae. William M. Wheeler, who coined the term, even suggested that trophallaxis would be the key to understanding eusocial behaviour in insects. Far from being passive colony members, pupae thus have an active and central role in ant colony organization.

We isolated pupae from the beginning of pupation until eclosion as adults, and found that they secrete a substantial amount of fluid towards the end of metamorphosis (Fig. 1a,b). Secretion
begins six days before eclosion, shortly after the pupae have melanized (Fig. 1a). The secretion initially accumulates in the exuvial space and then exudes from the rectal invagination of the pupal case (Extended Data Fig. 1), forming a stereotypical droplet on the abdominal tip
(Fig. 1b). Secretion continues until eclosion, ….. As long as we manually removed fluid daily, our social isolation protocol yielded high survival rates.

Adults consume the pupal fluid immediately as it is secreted, which is why the fluid does
not accumulate and become visible in the colony context. Indeed, adult ants pile up the pupae and remain in prolonged physical contact with them, frequently touching the pupae with their mouthparts…… adults were highly attracted to the fluid and rapidly consumed it…..

… if we did not remove fluid from isolated pupae under clean rearing conditions, they drowned in their own secretion … pupae depend on adults to remove the secretion, and would otherwise die of fungal infections… This fluid is secreted in large volumes by all pupae during a specific window of development, and elicits parental care behaviour that is necessary for pupal survival.

……young larvae are usually attached to pupae, often with their mouthparts, suggesting that they consume the liquid directly as it appears on the pupal surface (Supplementary Video 4).
Larvae have limited mobility and are thus dependent on adults to place them on food sources. Observations suggest that O. biroi adults readily place young larvae on pupae and older larvae on prey items

When given pupae alone, ants placed almost all larvae on pupae until eclosion (Fig. 3d). When given a choice, ants showed a strong preference for placing the larvae on pupae as opposed to prey

Fig. 1 | Ant pupae secrete a social fluid that elicits parental care behavior that is crucial for pupal survival.

It is difficult to understand why the nursing behaviors reported in Snir et al. were not the basis of nursing assays and less-convincing assays whose results are not clearly interpretable were substituted.

**Open Access** This Peer Review File is licensed under a Creative Commons Attribution 4.0 International License, which permits use, sharing, adaptation, distribution and reproduction in any medium or format, as long as you give appropriate credit to the original author(s) and the source, provide a link to the Creative Commons license, and indicate if changes were made.
In cases where reviewers are anonymous, credit should be given to 'Anonymous Referee' and the source.
The images or other third party material in this Peer Review File are included in the article's Creative Commons license, unless indicated otherwise in a credit line to the material. If material is not included in the article's Creative Commons license and your intended use is not permitted by statutory regulation or exceeds the permitted use, you will need to obtain permission directly from the copyright holder.
To view a copy of this license, visit https://creativecommons.org/licenses/by/4.0/

Referee #1 (Remarks to the Author):

The Kronauer lab has led social insect molecular neuroscience, including the development of CRISPR for application in insect sociobiology, using the clonal raider ant model. This is an advantageous model system due to genetic homogeneity and availability of molecular tools enabling the experimental alteration of gene expression. This molecular dissection of social behavior is step-by-step increasing our understanding of the mechanisms underlying sociality. The present submission represents potential for further contribution to this literature.

In this manuscript, the authors describe a series of detailed molecular analyses and experiments to investigate the role of neuropeptides in an aspect of potentially age-related behavior – care of larvae – in clonal raider ants. Starting from a large set of candidates, neuropeptides were synthesized and evaluated for potential influences on brood care. In a preliminary screening using less-pure neuropeptides, candidates were evaluated for their influence on worker/brood interactions by soaking individual workers in solutions containing each candidate molecule and then allowing treated workers to interact with larvae. In the behavioral assay, the time spent in contact with larvae was quantified using automated tracking; carrying larvae was also recorded. After selecting half of the neuropeptide candidates that showed the greatest effects on the type of interaction with larvae recorded in the assay, highly purified samples of the subset of neuropeptides were synthesized. Larval-care assays then focused on two neuropeptides: NPF and AstA2.
The authors measured change in expression levels of these two neuropeptides in young or old workers in specific areas of the brain using immunohistochemistry to quantify RNA and protein levels based on signal from fluorescent tags. This showed expression of NPF in the dorso-lateral protocerebrum and antennal lobes and expression of AstA2 throughout the brain. Increased worker age was associated with decreased NPF expression in four specific cells, with little change elsewhere and increased AstA2 only in the antennal lobes, with decreases or little change elsewhere. Levels of these neuropeptides were then manipulated using RNAi to decrease each in turn and by injecting each directly into the heads of ants to increase their titers.

The study is impressive in molecular detail. The conceptual framing and generalizability of the results and the validity of the larval care assay to the conclusions drawn, however, are problematic, as noted in “comments” below.

>> Thank you for the thorough review. We are pleased that the reviewer appreciated the molecular analyses. The main concern raised by this reviewer (and other reviewers) stems from an unfortunate ambiguity in the original submission as to how ants interact with larvae in our assay.

To address this, we have conducted an additional experiment and accompanying analyses (now presented in Figure 1) to thoroughly ground-truth the assay. In the new experiment, we generated behavioral ethograms of whole colonies to characterize natural nursing behavior, and we re-analyzed video data from our one-on-one assay to quantify

how ants interact with larvae in that context. The data clearly demonstrate that our assay captures nursing behavior rather than, for example, feeding. The tendency to perform specific components of nursing (such as guarding or transporting larvae) was highly correlated with time in physical contact with larvae. By contrast, interactions with food items - a control that was omitted in the original submission - were qualitatively different: food was largely ignored, and there was no difference between young and old ants in their behavior toward food.

Finally, following the reviewer's suggestion, we have removed references to 'exploration'. We now instead focus on 'time in contact with larva' and 'time away from larva', which more accurately capture the relevant behavioral dimensions.

Comments:

Line 1 and throughout the manuscript: "brood-care behavior"

>> Amended throughout.

The title could have more impact reflecting the novelty and significance of the work if it stated that two ancestral regulators of feeding underpin age-related alloparental care in the clonal raider ant. Still, issues related to feeding per se need to be clarified, as noted below.

>> We like this suggestion and have modified the title accordingly. It now reads: "Ancient feeding-related neuropeptides regulate alloparental behavior in the clonal raider ant". We have also updated the first line of the abstract to correspond. Thank you.

Line 17: This is the first reference to age-related division of labor being ubiquitous in social insects. Age-related worker behavior is presented as the simplified classical concept of temporal polyethism. While it may be common among the relatively small number of ant species studied, it is not universal and it is inaccurate to imply that all social insect workers follow this pattern of behavioral development. Some basal ant species have evolved patterns of age-related behavior in association with variation in life history, colony age demography, and the coupling of foraging with the provisioning of larvae. Other social insect species show plasticity in age/task relationships that may arise from experimental demographic alterations. The characterization of newly eclosed workers as discretized nursing "specialists" originally proposed by Wilson is not a generality. Related concepts include response thresholds (mechanisms and evolution) and self-organization of age-related behavior (the "foraging for work" model).

>> The reviewer is correct that temporal polyethism may not be ubiquitous in ants. We have revised the statement to read: "Social insect workers **typically** care for brood within the nest when they are young and transition to foraging outside the nest as they age[2–5]". This sentence is not intended to have any bearing on Wilson's proposal, the response thresholds model, or the foraging-for work-model; in our view it is consistent with all of these frameworks.

The generalizability of results of the starved/fed and young/old worker treatments as drivers of interaction with brood across all ants is not convincing. Some workers receive nourishment by interacting with brood, while others forage to provide food to larvae. Some specialized workers seldom interact with brood. This diversity of types of age-associated worker behavior and its relationship to the study is important to consider.

>> We agree with this point and have reworded the text where appropriate to avoid making that generalization. E.g., "Furthermore, we found that NPF and AstA brain levels change naturally as ants age, suggesting that these changes underlie the age-related reduction in brood-care **in the clonal raider ant**". We believe that the value of our work lies in showing that AstA and NPF have been co-opted to regulate brood care in *O. biroi*. It would be interesting if other neuropeptides prove to be important in other species, which may be the case given the ecological differences that the reviewer mentions.

Here and on line 37 and elsewhere it is therefore appropriate to refer to "age-related behavior" rather than "age polyethism" and to acknowledge the variation documented in the association of age and task performance, and related theories and concepts. With respect to this, Whitfield et al. 2003 should be cited, as it is a key paper on gene expression, behavioral development, and task transitions in honeybees.
Whitfield et al. (2006). Genomic dissection of behavioral maturation in the honey bee. PNAS 103, 16068316075

>> We have followed the reviewer's advice and have removed all references to 'age polyethism' (it was unnecessary field-specific vocabulary). We have also added a paragraph that contextualizes our study within broader work on the molecular regulation of other aspects of division of labour in other social insect species (L. 229-254), and we now cite Whitfield *et al*., 2003 in the abstract and main text (L. 22 & L. 38). We did not add discussion of the different theoretical frameworks of division of labor, as this seemed unnecessary after removing mention of age polyethism.

Additionally, physical caste evolution, like age-related behavior, should be noted in the context of the genetics/epigenetics of division of labor. Clonal raider ants are largely monomorphic, but other species of ants and other social insects produce specialized worker phenotypes, e.g., major workers or "soldiers," with head structure and mandibles sometimes modified in extreme ways for phragmosis or defense. These workers are task-specialized, likely throughout their lifespan. There is a significant literature on the genetics and epigenetics of task switching in physical caste division of labor that should be recognized and discussed. Task acceleration and reversion are important processes of plasticity in worker behavioral development. See for example:

Lucas, C., & Ben-Shahar, Y. (2021). The foraging gene as a modulator of division of labour in social insects. Journal of Neurogenetics, 35, 1683178.
Lucas, C., & Sokolowski, M. B. (2009). Molecular basis for changes in behavioral state in ant social behaviors. PNAS, 106, 635136356.
Simola, D. F., et al. (2013). Social insect genomes exhibit dramatic evolution in gene

composition and regulation while preserving regulatory features linked to sociality. Genome Research, 23, 123531247.
Simola D.F. et al. 2016. Epigenetic (re)programing of caste-specific behavior in the ant Camponotus floridanus. Science 351: 42

>> We have addressed the points raised here through a rewrite of the discussion, where we discuss the factors that have been found to be important in regulating worker behavior in *Harpegnathos*, *Camponotus* and *Atta.* We now cite the four papers mentioned by the reviewer along with other recent work on these species:

"AstA and NPF form part of an ancient, deeply conserved, and highly pleiotropic regulatory network[42,53]. This network centers around insulin, juvenile hormone (JH) and ecdysone and co-ordinates feeding, growth and reproduction with nutritional availability. Insulin modulates the synthesis of both JH and ecdysone, while JH and ecdysone are mutually antagonistic and ecdysone antagonizes the insulin and insulin-like signaling (IIS) pathway. NPF generally promotes JH synthesis, although the details of this relationship may vary between taxa[54,55]. The action of NPF-responsive neurons is inhibited by insulin, forming a negative feedback loop that co-ordinates feeding with nutritional status[56,57]. AstA generally inhibits JH synthesis and NPF neurons and stimulates insulin-producing cells[16,58,59]. Our data fit with an emerging pattern that elements of this regulatory network have been consistently co-opted to govern insect social behavior. This pattern is all the more striking for holding true across taxa with diverse social structures and (allo-)parental behaviors[24]. Both insulin and JH regulate the transition from nursing to foraging across social Hymenoptera[60–63]. Additional elements of this network have recently been shown to play important regulatory roles in ant species with social structures that differ from *O. biroi*[13,63,64]. In the leaf-cutter ant *Atta cephalotes*, labor is divided between morphologically distinct worker castes. Majors specialize in colony defense while minors specialize in brood-care. The behavioral differences between these castes are maintained by the neuropeptide Neuroparsin A (NPA), which is synthesized by insulin-producing cells in the brain and interacts with insulin signaling[13]. NPA additionally appears to regulate reproductive division of labor across social insects[65]. In the ponerine ant *Harpegnathos saltator*, worker behavioral roles are controlled by the neuropeptide corazonin[14]. Corazonin is homologous to vertebrate Gonadotropin-Releasing Hormone (GnRH) and in *D. melanogaster*, corazonin neurons modulate the activity of insulin-producing cells[66]. In the carpenter ant *Camponotus floridanus*, behavioral differences between majors and minors are controlled by a JH esterase in glial cells, which constitute the blood-brain barrier and control JH titers in the brain[63]. Across species, these age- and behavior-associated changes in neuropeptides are likely underpinned by epigenetic modifications[67,68]. Taken together, this suggests that NPF and AstA modulate brood-care behavior via their interactions with insulin and JH signaling, and helps explain why nutritional state, ageing, and division of labor are so intricately intertwined in social insects[69]."

Line 51: Regarding the brood-care assay, was the enclosure narrow and linear as seen

in the corresponding figure or are the linear sections just cropped to illustrate the distance between the worker and the brood?

>> The former. This has now been clarified in the text: “In this assay, individual ants are placed into a narrow corridor along with a single larva and are filmed for 5 hours (Fig.1d, Extended Data Fig. 1a, and Supplementary Video 1).”. We have also included an image of one of these setups in our new Figure 1 (panel 1d) to show the reader what exactly this looks like.

Line 56: Here and in other places, detail is provided later on, but clarity would be enhanced by briefly describing how a numerical threshold was used to differentiate “bouts of exploration” from periods of “direct physical contact”.

>> The measurements work as follows: ants and larvae are both modelled as ‘blobs’. Direct physical contact equates to any overlap between the ant and larval blobs and ‘bouts of exploration’ refers to periods where the blobs do not overlap. To improve the clarity of this section we have re-written it as follows (L. 68-70): “In the first phase (P1), ants spend most of their time in direct physical contact with the larva. In the second phase (P2), ants tend to leave the larva for short bouts as they move around the corridor (Fig. 2a-e, and Extended Data Fig. 1b).” There is no thresholding on the overlap versus non-overlap.

Line 76: Please further clarify how you tested the ability of candidate molecules to penetrate the cuticle. This is alluded to but a description of how this was tested seems missing, or at least hard to locate.

>> We have added the following sentence to the main text (L. 101-103): “The validation was performed by soaking ants in biotinylated neuropeptides and then dissecting out and treating their brains with fluorescent dye-conjugated streptavidin before conducting quantitative confocal microscopy.”

Line 77: Similarly, here briefly summarize the method used for protein synthesis even though it is explained in detail later.

>> We now incorporate methodological information at this point in the main text: “For our screening library, 61/70 neuropeptides were synthesized at the Proteomics Resource Center at The Rockefeller University using 25μM scale Fmoc solid-phase peptide synthesis[40]. Peptides were purified by liquid chromatography to purities between 15% and 92%. They were provided lipolyzed and ranged from 7 to 35 amino acids in length (Extended Data Fig. 4a).”

Line 133: Is there any reason to believe that NPF and AstA2 interact with each other, given that they have somewhat opposite effects? Is it more likely that their expression is independently regulated?

>> There is evidence of a direct interaction between NPF and AstA in *Drosophila* – NPF neurons are post-synaptic to AstA neurons and express the AstA receptor. Knockdown

of the AstA receptor in NPF neurons increases feeding (https://pubmed.ncbi.nlm.nih.gov/34018925/). However, it is currently unknown whether and how these two neuropeptides interact in ants. Therefore, to be more neutral about the mode of interaction, we have reworded the relevant sentence as follows (L. 153-156): "The age-associated changes in the abundances of these neuropeptides in the PC and AL could thus shift the internal state of the brain and underlie division of labor by modulating the neural circuitry that governs brood-care behavior (Fig. 4f)." We then discuss what is known about the interaction of NPF and AstA in other insects in the Discussion (L. 229-236).

Line 138: Again, while this is described elsewhere, briefly state that injections were made through the lateral aspect of the head.

>> These two sentences now read (L. 159-161): "To substantiate our screening results, we injected synthetic peptides to increase levels of NPF and AstA, and siRNAi to decrease levels of NPF and AstA. All solutions were injected through the lateral aspect of the heads of young ants."

Line 167: Consider adding in discussion of workers receiving food from larvae, e.g., larval hemolymph feeding, which may not be a factor in clonal raider ants but would be in others, or digestion and regurgitation of solid foods by larvae to feed to workers. The reason for this is that it should be considered whether workers would be satiated after foraging in all cases.

>> This is a nice example of how feeding-related neuropeptides may interact differently with brood care in different species, and we have incorporated this aspect of ant diversity into the new paragraph of the discussion (L. 283-285): "For example, in certain contexts, adults feed larvae mouth-to-mouth and interacting with larvae reduces the workers' nutritional status[80]. In other contexts, larvae regurgitate predigested food to feed adults, or adults pierce the sides of larvae and drink their hemolymph, in which case interacting with larvae increases the workers' nutritional status[81]."

Line 249: Does cleaning a porous ant nest floor with bleach while the colony is inhabiting the lab nest really not harm workers or alter their behavior or physiology? Chlorine dioxide is a fumigant pesticide known to produce oxidative stress in insects and mortality in ants. Mold inhibitors like Tegocept are non-toxic antimicrobial agents that could serve the same purpose.

>> We use this method regularly in the lab and have not observed any adverse effects. The quantities of bleach are minute. That said, we have not performed a rigorous survival analysis and perhaps the mentioned antimicrobial agent would be better. Thanks for this suggestion!

Line 293: Does this indicate that NPF "passed" the first screening in that it had an upper 50% level of significance of effect on changes in brood interaction behavior, but that in the second round its effect was significant but in the opposite direction? This needs to

be explained more clearly and the authors should include a comment on the impurities in the 92% pure synthesized compound that could have such a dramatic effect.

>> Yes, that is correct. Given the permissiveness of the first screen, we believe that NPF was in the upper 50% of significance effect there by chance (rather than due to impurities) and the results of the more stringent secondary screen should be considered valid. We have improved the clarity of that section, which now reads (L. 119-122): “However, because this result was inconsistent with the primary screen (Extended Data Fig. 4g), we repeated the dose response experiment. As expected, given the caveats associated with the primary screen, the dose response experiment confirmed the results from the secondary screen **and implied that the effect observed in the primary screen was due to chance** (Extended Data Fig. 4h-k).”

Line 306: Are the gel results or sequencing results included in a supplement?

>> There are no gel or sequencing results for protein synthesis. Purity was assayed by the supplier using analytical liquid chromatography and mass analysis by MALDI-MS. We have, however, now uploaded the sequences of the cloned AstA, NPF, and GFP fragments used for generating the dsRNA.

Lines 376-381: The P1 behavioral phase is defined as “the period beginning with larva detection and continuing while the ant remains mostly in physical contact with the larva,” independent of carrying the larva around or remaining in one location. “Larva carrying did not consistently occur across all assays or conditions, and it was therefore collapsed into the P1 phase.”
Do the authors rate larval transport higher than remaining in one position? What was the frequency of carrying larvae under control and experimental manipulations? Were grooming or feeding larvae recorded? Is there evidence of trophallactic transfer of crop or salivary gland complex secretions to larvae, perhaps based on the stain incorporated into the food?

>> The new Figure 1, which quantifies nursing behavior both in a colony context and in our one-on-one assay, should address this concern. We do not rate carrying higher than other behaviors. But carrying (and other nursing behaviors) were highly correlated with time in contact with larvae (Figure 1). Grooming was recorded within the category of ‘manipulating’, which was defined as the worker contacting the larva with both its mouthparts and antennae and moving these body parts. There was no feeding because no food was placed into the set-ups and workers do not feed larvae via trophallaxis in *O. biroi* – that has been experimentally tested by, as the reviewer suggests, feeding adults dyed food and later testing for the presence of the dye in larvae.

The P2 phase “was defined as the period when the ant begins to leave the larva and subsequently spends most of its time exploring the assay chamber away from the larva.” Time durations of the phases were also recorded.
Quoting the Abstract, the main findings are:

1. NPF increases the tendency of ants to nurse their larvae
2. AstA promotes exploration away from larvae

A major issue is that the assay seems to mainly record olfactory recognition of larvae, an element of brood care, but not nursing, either in respect to feeding, which is a key behavior in the study, or grooming. Perhaps workers in contact with or carrying larvae are moving them to new locations based on microclimatic variation in the artificial nest. Perhaps workers are in the process of evacuating brood. What was the change in larval location at the end of the assay? These are brood-care behaviors that are not necessarily coupled with nutrition, and the present study is focused on neuropeptides having conserved functions in feeding. Trophallaxis (and grooming, although still not necessarily directly related to nutrition) would be more compelling metrics of nursing. Unfortunately, it appears that current automated tracking can identify worker/worker trophallaxis, but not worker/larval food exchange, at least in honeybees, which feed larvae in cells, thus obscuring the behavior.

>> This point is important and was ambiguous in our initial submission. To address this, we have now recorded ants in a colony context to show that time in physical contact with brood is a robust proxy for the diverse facets of brood care (e.g., carrying and guarding) (Figure 1A). This is also true in the context of our assay (Figure 1B). Moreover, the same age-based differences in tendency to spend time with larvae emerge in the colony context and in our assay (Figure 1), but these differences do not emerge when ants are presented with food in our assay (Figure 1B). Importantly, adults do not feed larvae via trophallaxis in *O. biroi* and other doryline ants.

Apparently, in the present study, workers did not groom or feed larvae, and responsiveness to larval "begging" behavior was not observed or recorded. Responsiveness to brood odors could be quantified in a simple olfactometer and negative or positive phototaxis, which could be associated with brood care or foraging (or at least nest departure), respectively, could easily be recorded. "Exploring" is undirected movement in an assay chamber with atypical dimensions and structure relative to a natural nest. "Exploring" is also behavior that is not described by its consequence, its function cannot therefore be identified with certainty. It would be speculative to refer to movement away from larvae as "foraging" or any behavior related to nutrition. To examine the effect of nutrition on brood-care behavior, starved and fed ants were tested in the above assay. "Starved ants spent significantly more time with larvae than fed ants." However, this is positional data that although relevant to brood care does not establish a feeding relationship.

>> Grooming accounted for a small fraction of time spent in physical contact with larvae (Figure 1) and the reviewer is correct that workers did not feed the larvae. It would not have been possible for them to feed the larvae in our set up as no food was introduced and *O. biroi* workers do not feed larvae via trophallaxis. The reviewer is correct that we do not know the function of the 'exploring' and so have reworded throughout the manuscript to talk about 'time in contact with the larva' and 'movement away from the

larva'. The final point appears to be a misunderstanding. We do not claim that there is a feeding relationship but rather that starved ants perform more brood care than fed ants.

Lines 408-413: A statistician might be consulted to comment on this "permissive" test.

>> The results of this permissive test are used only for an initial screening to select promising candidates, and these candidates are then tested in a statistically robust manner, including with dose-response curves. The only downside of this approach is that at the primary screen we may have eliminated neuropeptides that play important roles. We are consequently careful not to claim that neuropeptides other than AstA and NPY are not important.

Line 743: Behav. Ecol. Sociobiol.

>> Corrected, thank you.

Other comments on references:

In referencing the genes/hormones/circuits approach, older Robinson lab studies could be updated by:

Sinha et al. (2020). Behavior- related gene regulatory networks: A new level of organization in the brain. PNAS 117, 23270323279.

>> Done, thank you.

In summary, this is an interesting study that could improve its conceptual framing and provide more compelling evidence of the role of neuropeptides in nursing behavior in the clonal raider ant as well as ants in general.

>> We are grateful to this reviewer, whose feedback has substantially improved our manuscript.

Referee #2 (Remarks to the Author):

This is an impressively complete and detailed examination of how specific neuropeptides, generally known to function in feeding behavior, regulate alloparental care in a clonal ant. The work is detailed, the evidence compelling and complete, and represents a major advance in our understanding of how social behavior might be regulated in social insects.

>> Thank you.

The experimental design is clever and especially so working on a non-model organism. The full toolbox of genetic techniques are not the same as you would have for genetic model organisms such as Drosophila, yet the authors provide multiple lines of evidence that go well beyond correlation and association to bolster their interpretations. The manipulations are important and appropriate, and the range of approaches and their analysis is well conceived and executed.

>> We are glad to see this appreciated!

I have one minor question over the setup of the study. While the authors say "why neuropeptides", I think they can be clearer that pleiotropy is expected in specific neuropeptides rather than novel genes. There has been a number of studies that have suggested this hypothesis. The novelty and potential universality of the work would then be clearer.

>> We agree with the reviewer that this could have been clearer. We have reworded part of the introduction (now L. 44-45): "Accumulating evidence suggests that novel social behaviors frequently evolve through the co-option of ancient neuromodulators, including neuropeptides, biogenic amines, and hormones[6–11,13,14,18]". We have also added a discursive paragraph where we consider our results in light of existing hypotheses within the social insect literature regarding the evolution of social behaviors (L. 267-277):

"Various overlapping hypotheses have been proposed to explain the molecular evolution of social insect behavior. The ovarian ground-plan and reproductive ground-plan hypotheses predict that molecular factors that ancestrally governed foraging and reproduction have been co-opted to control queen-worker dimorphism[20,76,77]. The maternal heterochrony hypothesis posits that worker behavior evolved through the molecular mechanisms that ancestrally governed parental care becoming active prior to or without having reproduced[78]. Our data, together with recent data from across taxa, support these hypotheses and simultaneously reveal them to capture only part of a broader evolutionary trend: An ancient and deeply conserved regulatory network pleiotropically controls feeding, growth and reproduction. Independent origins of (allo-)parental behavior, from insects to mammals, appear to have convergently co-opted elements of this network[21,22,24]. This suggests that the evolution of (allo-)parental behavior is mechanistically more constrained than previously thought[24]."

Minor comments:

92 – note that NPF is homologus to NPY in vertebrates with the same association with feeding behavior. AstA receptor is homologus to Galanin receptor. Thus, I think you can expand the potential relevance of your work.

>> We have reworded these sentences to emphasize the homologies (L. 114-118): "Out of the 12 tested candidate neuropeptides, the secondary screen recovered two hits: NPF (encoded by the precursor gene *pro-neuropeptide-y-like* (LOC113562173)) and AstA (the precursor gene *AllatostatinA* (LOC105283611) encodes five AstA peptides but the effect was specific to AstA2) (Fig. 3a). These neuropeptides are evolutionarily deeply conserved, with NPF being homologous to vertebrate NPY and the AstA receptor being homologous to the vertebrate galanin receptor[41,42]."

230 – finally get the information on ubiquity of genes studied. Could be made earlier. Excellent study that carefully documents role of NPF and AstA in alloparental care. The work is unique in being complete – both manipulative studies, identification of brain regions as well as associations with behavior. The work is elegant and compelling.

>> Thank you! Hopefully emphasizing the homologies earlier in the main text will help.

My main concern is a lack of interpretation of the importance of the work. It somehow feels very specific to ants, but I think there is a greater global relevance. This could be made clearer if we were told about other studies of NPF and AstA and parenting – e.g., do these show up in trascriptomic association studies? I believe NPF has been associated with parenting, but I am less sure of AstA. What about NPY and GAL?

>> This is an important concern - to address this, we have reworked the discussion, including other studies on NPY and AstA (/GAL), and providing a broader conceptual framework to interpret the results (L. 256-265): "Parallel research into the regulation of vertebrate parental care has found that, as in social insects, molecular factors that ancestrally governed feeding, growth and reproduction have been convergently co-opted to regulate caregiving across taxa. The vertebrate neuropeptide NPY, a homolog of NPF, is a primary driver of feeding, and appears to have been co-opted to modulate caregiving at independent evolutionary origins of parental behavior in frogs, birds and mammals[70–72]. Virgin female mice typically care for pups but will aggress pups when hungry. Recent work shows that this switch is mediated by the release of NPY, which inhibits care-promoting neurons in the medial preoptic area (MPOA) of the hypothalamus[73]. Galanin, the receptor for which is homologous to the insect AstA receptor, is also expressed by hypothalamic neurons in mice, and optogenetic manipulation of these cells can toggle male behavior between parental care and pup-directed aggression[72,74,75]."

Perahaps part of the problem is a very ant-centric focus. Why is "alloparenting" important – is this just a form of care involving siblings or is it somehow different than parental care? I realize that these ants only have alloparental care (and not parental

care), but for those of us who don’t study ants does this matter at all? What is the global importance – is it just for ants and perhaps other eusocial insects or does it imply relevance beyond eusocial insects and even perhaps insects?

>> The re-written discussion now highlights the broader relevance of our results. The reviewer is correct that the results are useful in understanding how parenting behaviors evolve in general, rather than being limited to allo-parenting in ants.

I think the paragraph from lines 230-239 is very important (note my comment above when I first read line 92-) but we need something in the introduction suggesting this is coming. Did you predict that you would see a co-option of feeding related neuropeptides? Others certainly have. Perhaps this can be highlighted in a sentence in the introduction.

>> We have reworked the introduction to emphasize this and agree with the reviewer that the text is improved by this restructuring (L. 44-48): “Accumulating evidence suggests that novel social behaviors frequently evolve through the co-option of ancient neuromodulators, including neuropeptides, biogenic amines, and hormones[6–11,13,14,18]. An emerging pattern is that neuromodulators that have been co-opted to regulate social behavior have conserved pleiotropic roles in feeding, growth and reproduction, suggesting that involvement in these processes may predispose neuromodulators to be co-opted into regulating parenting-associated social behavior[19–25].”

Referee #3 (Remarks to the Author):

Summary: The authors developed an automated behavioural assay to monitor ant attentiveness to larvae and used it to screen 70 neuropeptides (exposed by submerging ants in a solution of the peptide). They found several potential candidates and explored two, one promoting nurse-like behaviour and one foraging. Through careful testing, with RNAi manipulations, neuropeptide supplementation and immunohistochemistry, they show that these change with ant age and feeding status, potentially underlying age-related polyethism.

Overall, they do a wonderful and thorough job of showing something that isn't terribly surprising, that neuropeptides regulate hunger and satiety in ants. Neuropeptides are well conserved. They've been studied in many insects. As the authors say, these ones are known to regulate feeding.

>> We appreciate this reviewer's feedback, which drew our attention to an important ambiguity in the original submission that we have now rectified. Our experiments show that, in addition to their ancestral association with nutritional status, the neuropeptides specifically regulate brood care behavior (not feeding). This is an entirely new discovery, and it represents a major piece in the puzzle of how alloparental behavior evolved in ants. We have added a new Figure 1 to illustrate this. In a first experiment, we characterize the behaviors that comprise 'nursing' in a colony context, demonstrate that time in contact with larvae is a suitable proxy for these behaviors, and that these behaviors are performed more frequently by young ants than by old ants (Figure 1A-C). Second, we characterize how young and old ants interact with a larva *vs* with an item of food in our brood care assay. We show that (i) the behavior towards the larva in our assay recapitulates brood care behavior in the colony context, (ii) that behavior towards a food item is clearly and qualitatively distinct, and (iii) that young and old ants behave differently towards brood but not differently towards food in our assay (Figure 1D-F). Importantly, in none of our assays did we observe any cannibalism of larvae, and when isolated with a food item as a control the ants did not feed (which is typical of isolated ants). In sum, the new Figure 1 provides an important ground-truth that was missing from the initial submission.

They do not however discuss much about how this work interrelates with their own work and others' on other neuropeptides involved in social life, caste behaviors and foraging. Also some relevant citations are missing/not discussed:
e.g. https://doi.org/10.1002/cne.24109, https://doi.org/10.1371/journal.pone.0109590

>> Following this advice (and similar advice from Referee #2) we have added a discussion paragraph where we place our results in context of other recent studies on how neuropeptides regulate social behavior in insects (L. 229-254). And thank you for providing these references – they are useful and we now cite both.

The most interesting result in my mind is that starvation induced these ants to stay longer with brood. This is quite opposite to what was shown with another very different clonal ant: https://doi.org/10.1242/jeb.219238 (your ref 35). It leads to many more

questions: Is cannibalism observed? Is this some more interesting decoupling of nutritional state from behavioural outcome via the neuropeptide signal? Fundamentally, is the clonal raider ant the odd one out here? And if so, why would that be?

>> This point is indeed interesting (even though, in our opinion, it is certainly not the most important finding of our study). We observed no cannibalism, and all larvae were alive after our experiments. We also observed no feeding when the ants were provided with food instead of a larva. In *O. biroi*, larval hunger rather than adult hunger is the stronger determiner of adult foraging effort, but the relative contributions of adult and larval hunger to adult foraging appear species-specific. The *P. punctata* and *O. biroi* results do contrast, and *O. biroi* is the outlier in that better nourished individuals forage more (https://doi.org/10.1242/jeb.219238). This difference could be linked to the phasic reproductive cycles of *O. biroi*. *P. punctata* produces larvae and forages continuously, but brood development is synchronized in *O. biroi,* and when the larvae pupate, foraging arrests for three weeks. This could have provided a selective pressure to decouple nutritional state from behavioral outcome. We emphasize this point on L. 200-206: "However, this result is consistent with a previous study that showed that well-nourished clonal raider ants in fact forage more than starved ones, possibly due to evolutionary changes in the underlying endocrine pathways and neural circuits that link nutritional state to foraging behavior in this species[52]. The inversion of the ancestral relationship between hunger and foraging may relate to the phasic lifecycle of the species, in which workers undergo regular periods of hunger during a reproductive phase where foraging activity ceases. These periods are then followed by a foraging phase where both foraging and feeding increase[27]."

Otherwise, I had a handful of issues and comments:

Figure 2: The notable differences in control (0 treatment) conditions between corresponding panels (b/f, c/g, d/h, e/i) require explanation. This inconsistency potentially impacts the interpretation of treatment effects and requires experimental design clarification.

>> The top and bottom row panels represent different experiments, meaning that workers were collected from different stock colonies at different times. While we gave ants *ad libitum* access to food 24 hours prior to conducting the experiments and used colonies with 4$^{th}$ instar larvae, colonies would still vary in exact hunger level, exact day through the colony cycle, stress level, *etc,* which could all affect the baseline level of interaction with larvae. Importantly, this variation is blocked within experiments.

L45-47: The perspective presented aligns with current understanding in the field, so not a particularly novel hypothesis.

>> The reviewer is correct. The interesting perspective is not so much whether neuropeptides are involved, but rather which neuropeptides are important, and what were their ancestral roles. We have reworked the introduction accordingly.

The choice of the clonal raider ant as a model system presents certain limitations for studying age-related polyethism. Given their cycling behavior, the point at which they might exhibit age-related polyethism seems narrow and the dynamic range in the data seems to reflect that. It seems like a caveat that the reader should be aware of. Nearly all other ants have a much stronger shift between states. It might be worth showing that you can recapitulate these neuropeptide supplementation results in a more normal ant.

>> This seems to be a misunderstanding. Clonal raider ants can live for well over 6 months, during which they will cycle through the reproductive phase and foraging phase many times. During the foraging phase, the colony functions like a normal ant colony, with nurses and foragers. Individuals <3 months old typically engage in nursing while foraging is biased towards individuals > 4 months old. The shift appears to be as strong in the clonal raider ant as in "typical" formicine and myrmicine ants. We have clarified this at the end of the introduction: "Workers exhibit a typical age-linked transition from brood-care to foraging. Young ants (12 days old) spend over twice as much time performing brood-care as old ants (4 months old) and less than half as much time foraging (Fig. 1a,b)."

Including citations for the referenced work at L69 would enhance the paper's scholarly context and provide appropriate recognition of prior contributions to this research area.

>> These have been added, thank you for pointing this out.

Also you have a lost citation on L71.

>> Corrected – thank you.

The soaking method. How do you think the neuropeptides get through the cuticle? Seems awfully risky to be so porous… Ingestion? And then to the brain??

>> We think that the neuropeptides most likely get in through the trachea, which contain a liquid/gas interface where oxygen absorption and carbon dioxide release occur - the liquid may enter the body where the trachea terminate between cells. The clonal raider ant brain has trachea, obvious in brain dissections, that cover it and enter the brain tissue. This may be the route neuropeptide solutions take to the brain in the soaking method. Similarly, trachea elsewhere in the body may allow neuropeptide solutions to enter more broadly.

Thank you (on behalf of future researchers) for providing the LOC numbers and XP numbers throughout!

>> We are glad that this is appreciated.

L95-98: Can you clarify the differences here and what was inconsistent and why?

>> We have clarified and explained this inconsistency (L. 118-123): “NPF increased the amount of time ants spent with larvae in the secondary screen (Fig. 3b-e). However, because this result was inconsistent with the primary screen (Extended Data Fig. 4g), we repeated the dose response experiment. As expected given the caveats associated with the primary screen, the dose response experiment confirmed the results from the secondary screen and implied that the effect observed in the primary screen was due to chance (Extended Data Fig. 4h-k).”

The manuscript would benefit from a clear explanation of the complementary roles of siRNAi and dsRNAi in your experimental design. Additionally, a rationale for emphasizing the siRNAi results over what seem to me as more robust dsRNAi data would help readers fully appreciate your methodological choices.

>> The dsRNAi method used long double stranded RNA covering the coding sequences of the targeted mRNAs to trigger the RNAi response. AstA used a ~566bp dsRNA, NPF a ~345bp dsRNA, and GFP a 546bp dsRNA. These long dsRNA’s get cleaved into several individual short RNA molecules (21-23bp) by Dicer creating several siRNA products, theoretically as many as 17 – 26 targeting the mRNAs for AstA, NPF, and GFP. These siRNAs then can couple with the RNA-induced silencing complex (RISC), where each siRNA guides the silencing complex to the target RNAi. As multiple RNAi silencing complexes target multiple sites across the mRNA the knockdown effect can be robust. This likely led to the robust behavioral effects, and the ability to detect knockdown both quantitatively and qualitatively.

In contrast, the siRNAi method used single 27bp Dicer substrate RNAs (DsiRNAs) targeting each of AstA, NPF, and GFP. These DsiRNAs are efficiently cleaved by Dicer to siRNAs that complex with RISC leading to knockdown of the targeted mRNAs. However, these siRNAs only target a single site on each mRNA, leading to a less robust knockdown effect.

One rationale for including both methods is this: they are independent methods for knockdown that provide confidence to the RNAi method and resulting behavioral effects, which importantly changed the behavior of the ants in the same direction regardless of the method. However, the dsRNA yielded robust knockdown, hence greater behavioral effect sizes and more of our behavioral measures changed enough to reach statistical significance despite the inherent variability in behavioral measures. The siRNAi method yielded milder, though significant, knockdown, hence smaller behavioral effect sizes and only the P1 phase measure achieved statistical significance.

The rationale for using the siRNAi results in the main figure was that we performed this experiment first and the results are simpler and take less space to display and highlight the main point. We have now included the following sentences (L. 175-179): “We confirmed the siRNAi results using an independent RNAi method. In this experiment, we injected long double-stranded NPF or AstA RNA into ant heads to stimulate RNAi knockdown (dsRNAi). These long RNA strands are cleaved into multiple siRNA products

that cover multiple sites across the target mRNA leading to more robust knock-down than the siRNAi method, which targets only a single site per mRNA.”

L185~ How does all of this actually relate to larval care? From the videos and descriptions, it doesn’t sound as though they are actually doing much caring for the brood. Most other ants feed their brood orally, with prey or with trophic eggs – what are these ants doing with them during the brood care phase? It is just that they notice a larva and then look around fro something to feed it? Is this why they never come back to the larva?

>> This is addressed with Figure 1, where we characterize ‘brood-care’ in a colony context, and characterize the behavioral interactions between the ant and the larva in our brood care assay. In both contexts, brood-care entails (i) larval transport, (ii) larval manipulation (i.e., contact from the workers antennae and mouthparts to the larva and rapid movement of these body parts), (iii) larval guarding (standing stationary over the larva). In the colony they also place larvae on food, but *O. biroi* adults do not feed larvae via trophallaxis.

The paragraph on the interplay with nutrition, JH, insulin, and these neuropeptide is nice, though I feel that the difference with the clonal raider ant results and other ants and solitary insects could be explored more clearly.

>> We have now re-written the Discussion and expanded upon this in paragraphs 2-5 of the new discussion.

L288. It is hard to see how one can conclude much about something applied at 15% purity.
While power analyses were performed, the sample sizes (n=12 for behavioral assays and n=5 for brain analyses) appear insufficient given the data variability (e.g. Fig 3). Increasing sample sizes would strengthen the robustness of the findings. Figure 5 has larger n and is much more compelling.

>> We have increased the sample size for the brain analyses. The new results have revealed a greater similarity between the effects of ageing and nutritional status and have updated the model of our overall understanding (Fig. 6g). Thank you for this suggestion!

15% purity is indeed far from ideal, but we only used the low purity neuropeptides for the initial screen and performed a more robust secondary screen with higher purity neuropeptides (>95%). Consequently, the low purity could only have led us to disregard some neuropeptides that are in fact important to brood care. Because of this we are careful not to claim that AstA and NPY are the only important neuropeptides.

Thank you for the Annotation of NPF section.

>> Thank you for the thorough review. The feedback has helped us to clarify the

manuscript and the additional immunohistochemistry data has made the results more compelling.

Referee #4 (Remarks to the Author):

This study presents intriguing results, obtained using an impressive variety of methods, on the role of two neuropeptides in interactions of workers and larvae in the clonal raider ant.

The main result in Fig 1g is that a young ant, when placed in a narrow track with a larva, spends more time about 3 mm closer to the larva than an older ant. The behavioral assay consisted of placing a single ant in a 30 by 2mm track with a larva. The ant has to pass the larva as it goes back and forth along the track. The data were based on a measure by image analysis of how aligned were the bodies of the worker and larva, apparently indicating that the worker held the larva in its mandibles. The ant could have been inspecting the larva as a possible food item, or even moving it out of the way. In any case, proximity to a larva is not necessarily nursing, or 'brood care' as it is called in Figure 1. Brood care, or nursing, is a social activity performed by groups of ants in response to stimuli from groups of larvae (e.g. see Cassill's pioneering work on interactions between workers and larvae that stimulate feeding, and subsequent work by Dussutour). In some species, particular workers prefer to care for larvae at specific developmental stages (e.g. Pharaoh ant Warner et al. PLoS Genet 2019); if that occurs in clonal raider ants, the choice of worker may influence the results. It is also dubious whether an ant moving back and forth within a narrow track where it is confined could be called 'exploring'. The ant has no choice but to move along the track.

>> The reviewer highlights an important issue that was also raised by reviewers 1 and 3, namely what is entailed by physical contact between the ant and the larva in the brood care assay. To address this, we have conducted additional experiments and behavioral annotations. We now included a new Figure 1 where we characterize the facets of brood care in a colony context and related them explicitly to the behaviors that the ants show towards larvae in our assay. This ground-truths our assay in a way that substantially improves the manuscript, and we are grateful for the feedback. Importantly, Figure 1 shows that ants display nursing behavior in the assay that is similar to the behavior exhibited in a colony context, and that time in physical contact with the larva is a suitable proxy for nursing behavior.

The reviewer is also correct that 'exploring' ascribes a motivation to the ant's movement away from the larva that we do not have evidence for. Consequently, we have removed reference to 'exploration' and instead discuss simply 'time in contact with larva' and 'time away from larva'.

The results of this study may be more related to feeding behavior than to brood care. NPF and AstA regulate feeding other animals. Previous work by these authors showed that association with larvae is related to starvation. The results here on gene expression further support that: NPF levels increased in the WB, adPC, and AL of starved ants, while AstA levels increased in the WB, PC, AL, and SEZ of fed ants. Perhaps the young ants, who were taken from a brood chamber in the nest for the behavioral assay, were familiar with the smell of a larva, so spent slightly more time near it as a possible food

source, while the older ants, who were likely to have been foragers in the nest they were taken from, (although this is not explained in the methods) were more likely to recognize that the conspecific larva was not the S. invicta larva they were usually fed, and to continue past it when going back and forth in the tube.

>> This is another important issue. We have now included a control experiment where we pair ants with a food item (an *S. invicta* larva) instead of with one of their own larvae. Both young and old ants largely ignore the food item and display qualitatively different behavior towards the food item compared to their own brood. Additionally, there was no feeding when the ants were isolated either with a food item or with their own brood (i.e., no cannibalism). This, together with the characterization of the interactions between the adults and larvae in the colony context and in our assay (Figure 1), demonstrates that the ants display brood care in our assay and are not treating their own larva as a possible food source.

Thus the interpretation of the results far outreaches the behavioral data. Proximity of a lone worker and a larva in a narrow track, which may be related to feeding behavior rather than brood care, becomes 'affinity to larvae' which then becomes a 'nurse-like behavioral state', which then leads to the conclusion that the two neuropeptides 'regulate brood care' which then becomes an 'evolutionarily conserved' mechanism that regulates division of labor.

>> Thank you for raising this issue so clearly. It has prompted us to conduct additional experiments and analyses to show that time in proximity with larvae is a suitable proxy for nursing, and that the neuropeptides indeed regulate brood care behavior specifically. We believe that our interpretations are now fully supported by experimental evidence.

Regarding the results, aside from how they are interpreted: Lines 95-99 describe a result in the secondary screen that was inconsistent with the primary screen, as shown in Fig 2a and Extended Data Fig 4g. Fig. 2b–e shows that NPF increases P1 interaction time, while Extended Data Fig. 4h–k, shows that NPF increases P1 duration and total interaction time, but not P1 interaction time. The authors point out this inconsistency but do not explain why they decided to accept one result and reject the other.

>> The primary screen was intended to narrow down the exhaustive and long candidate list (>60 peptides), but not to conclusively show that any given neuropeptide is involved in brood care. The secondary screen uses pure, high-quality neuropeptides and includes dose response curves, which are the gold-standard. Hence the results of the secondary screen are accepted over the primary screen. We have clarified this on L. 120-123: “As expected given the caveats associated with the primary screen, the dose response experiment confirmed the results from the secondary screen and implied that the effect observed in the primary screen was due to chance (Extended Data Fig. 4h-k)”.

To investigate how NPF and AstA regulate behavioral transitions, the authors manipulated the levels of both neuropeptides in the brains of young ants. To increase NPF and AstA levels, they injected synthetic neuropeptides; to decrease their levels,

they used both dsRNAi and siRNAi. Both RNAi approaches produced consistent results. These manipulations were performed only in young ants, while the interpretation seems to suggest effects on older workers.

>> Our initial results indicate (i) that NPF levels are high in young ants, which do more brood care, and that (ii) AstA levels are high in old ants, which do less brood care. Consistent with this, our functional manipulations indicate that increasing NPF levels can increase brood care (at least in young ants) and increasing AstA levels can decrease brood-care (at least in young ants). This is sufficient to show that these neuropeptides causally regulate brood care behavior in *O. biroi*, which is the strongest of the claims that we make. It is not clear to us why one would hypothesize that the effects of these neuropeptides vary qualitatively by age – there is no basis to hypothesize that from our data or from the literature.

The authors suggest that the ratio of NPF and AstA regulates age polyethism (line 132). This interpretation requires further evidence. As shown in Figure 2, AstA is broadly expressed in the brain, with notable changes in the AL, SEZ, and CA. In contrast, NPF changes were limited to just four cells in the adPC. The authors proposed that an age-related shift in the NPF-to-AstA ratio underlies age polyethism. Because NPF and AstA are altered with age in distinct brain regions, and AstA shows a much broader distribution than NPF, further work is needed to explain where in the brain the effects of the two neuropeptides would act, or where the ratio between the amount of NPF in the adPC and the amount of AstA would be evaluated.

>> Perhaps the use of 'ratio' was a poor choice of wording. We did not intend to imply direct molecular interaction between these neuropeptides. That paragraph now ends (L. 151-156): "The decrease of NPF peptide in the PC and AL, and the increase of AstA peptide in the same neuropils with age, correlate with the results of our pharmacological screen and are consistent with our finding that NPF promotes nursing and AstA promotes foraging. The age-associated changes in the abundances of these neuropeptides in the PC and AL could thus shift the internal state of the brain and underlie division of labor by modulating the neural circuitry that governs brood-care behavior (Fig. 4f)."

The general hypothesis presented here, that alloparental care is the result of the modulation of neuropeptides, is a form of the 'reproductive groundplan' hypothesis introduced by Mary Jane West-Eberhard in Developmental Plasticity and Evolution (2003), suggesting that the evolution of sociality is based on the modulation of physiological triggers for feeding larvae. It is odd that this body of work is never mentioned.

>> This is an important point. We now mention this work in the introduction and dedicate a paragraph of the discussion to how our work relates to this hypothesis. As the reviewer points out, our results are consistent with the reproductive groundplan hypothesis in that ancestral neuropeptides have been co-opted, but differ in that the relevant peptides regulate feeding rather than reproductive behavior. The paragraph in the discussion (L.

267-277) reads: “Various overlapping hypotheses have been proposed to explain the molecular evolution of social insect behavior. The ovarian ground-plan and reproductive ground-plan hypotheses predict that molecular factors that ancestrally governed foraging and reproduction have been co-opted to control queen-worker dimorphism[20,76,77]. The maternal heterochrony hypothesis posits that worker behavior evolved through the molecular mechanisms that ancestrally governed parental care becoming active prior to or without having reproduced[78]. Our data, together with recent data from across taxa, support these hypotheses and simultaneously reveal them to capture only part of a broader evolutionary trend: An ancient and deeply conserved regulatory network pleiotropically controls feeding, growth and reproduction. Independent origins of (allo-)parental behavior, from insects to mammals, appear to have convergently co-opted elements of this network[21,22,24]. This suggests that the evolution of (allo-)parental behavior is mechanistically more constrained than previously thought[24].”

Referee #1 (Remarks to the Author):

The authors have responded to the issues I raised in my first review; I appreciate their effort. However, a key concern remains.

>> We are glad that, apart from the below concern, the reviewer feels that the issues raised during review have been addressed.

It is important to completely understand the nature of brood care in order to confirm the validity of the lab brood-care assay, and thus the role played by neuropeptides. I read authors' recent publications to better understand the natural context of brood care in clonal raider ants and compared this to the design of their brood-care assay.
First, Snir et al. (2022) describe the role of pupal milk and provisioning with prey in nutritional nursing:

"Ant pupae extrude a secretion derived from the moulting fluid that is rich in nutrients, hormones and neuroactive substances. This secretion elicits parental care behaviour and is rapidly removed and consumed by the adults. This behaviour is crucial for pupal survival; if the secretion is not removed, pupae develop fungal infections and die. Analogous to mammalian milk, the secretion is also an important source of early larval nutrition, and young larvae exhibit stunted growth and decreased survival without access to the fluid." Furthermore, "social fluid contains a variety of micro- and macronutrients" and thus has nutritional value.

And:
"This fluid is secreted in large volumes by all pupae during a specific window of development, and elicits parental (sic) care behaviour that is necessary for pupal survival." From Fig. 3 in this article, "Pupae secrete their moulting fluid and adults place larvae on pupae. Larvae and adults consume the fluid, and fluid consumption prevents pupal infections and death." Therefore, pupal milk removal is an important, perhaps critical, element of brood care.

Second, concerning direct provisioning with prey, "Larvae have limited mobility and are thus dependent on adults to place them on food sources. Observations suggest that O. biroi adults readily place young larvae on pupae and older larvae on prey items." Snir et al (2022) describe the complexity of brood care in clonal raiding ants. Therefore, I hope the authors can elaborate on how their relatively simple assay relates to this complexity. It seems that assays could have been developed that directly test elements of brood care that have functional assessments.

>> The pupal milk described in Snir *et al*., 2022 is interesting but not relevant for most of larval development because of the particularities of the clonal raider ant life cycle. Importantly, it does not play a role for any of the life stages examined in the current study.

In clonal raider ant colonies, reproduction is synchronized: workers lay eggs together over the same 2-3 days. Larvae hatch from these eggs after 10 days. The larval stage lasts for ca. 3 weeks and is subdivided into four larval instars. At the end of larval development, all individuals synchronously pupate and enter metamorphosis. Adults lay the next cohort of eggs ~5 days after the previous cohort has pupated. This means that two cohorts of offspring overlap – one at the pupal stage and one at the egg stage. The eggs typically hatch ~4 days before the pupae of the previous cohort emerge as adults. For these ~4 days, the colony does not forage and has no external food available. Colonies only begin to forage once the young adults have emerged from the pupae and the larvae of the next cohort have reached the 3rd instar.

The dynamics described by Snir *et al*., 2022 occur during the short 4-day window in which pupae overlap with young (1st and 2nd instar) larvae. The 4th instar larvae used in this study never encounter pupae and never consume pupal milk. This was previously unclear from the main text, and we now disambiguate this in the first paragraph of the results by specifying the larval instar that we are studying (L. 59 & 61). Additionally, we have added a sentence in the methods section (L. 320-321) stating that: “At this point in the colony cycle, only larvae and adults are present”.

The authors state that such issues are addressed by the information presented in Figure 1. Nevertheless, brood-care behavior is characterized here as larval transport, larval manipulation, and larval “guarding” (what are they “guarding” against”?). The authors note in the response letter that “in the colony they also place larvae on food, but O. biroi adults do not feed larvae via trophallaxis.”

Although I understand assays may have to be simplified to assess complex behavior, it is unclear why what would appear to be the most significant components of larval care are not reflected in the present design. The authors note “…a stereotypical two-phase response to larvae. In the first phase (P1), ants spend most of their time in direct physical contact with the larva. In the second phase (P2), ants tend to leave the larva for short bouts as they move around the corridor”. “Transporting” and “manipulating” concern brood care in a general sense, and perhaps captures a portion of age-related alloparenting, but brood-care behavior is much more involved in clonal raider ants. I assume worker responsiveness to pupal milk can be measured, and prey could be provided to record their placement on larvae. These evaluations are ethologically robust and accurate reflections of brood care in this species.

>> The important point here is that an ant's tendency to perform all nursing-related tasks within a natural colony are highly correlated. They are correlated because most ant workers are either nurses or foragers – distinct behavioral states underpinned by distinct brain states (Richardson *et al*., 2020; https://doi.org/10.1016/j.cub.2020.05.038). Across ants, there is little evidence for additional behavioral specialization among nurses. The nurse brain state makes ants more likely to perform all nursing-related tasks and more generally underpins a higher tendency towards interacting with brood. The elegance of our assay lies in the fact that it allows us to evaluate the ants' position on the nurse to forager spectrum by quantifying well-established aspects of nurse and forager behavior. However, it is evidently impossible (and unnecessary) to measure all aspects of the behavioral differences between these two states. This is what Referee #2 alludes to when they write that "Studies of behavioral genetics typically rely on an assay to reflect what can be a complex behavioral category (such as parenting) and I find that their assay is as good as any I've seen." Figure 1 shows that the behaviors captured in our assay constitute core aspects of brood care and that our assay serves as a reliable and quantifiable proxy of nurse-like behavior.

Ants frequently exhibit guarding behavior in laboratory colonies – naturally these workers would be the front-line if an intruder entered the nest, although of course, that does not happen in the laboratory context. The reviewer is correct that trophallaxis is absent in *O. biroi*.

Referee #2 (Remarks to the Author):

This is exciting work that demonstrates that NPF and AstA are coopted from an ancestral function in self-feeding to influence alloparental care. The novelty is threefold: first, this is the most complete examination, incorporating developmental changes as well as behavioral changes because of the unique system they have for study; second, the work is pleasingly complete with multiple avenues of investigation and experimental support; and third, this work shows that parenting in general, from mice to fish to eusocial insects, involves the cooption of neuropeptides with an ancestral role in feeding.

The authors have adequately addressed all of the previous concerns, with a much more robust demonstration of how their assay reflects alloparental care. Studies of behavioral genetics typically rely on an assay to reflect what can be a complex behavioral category (such as parenting) and I find that their assay is as good as any I've seen.

The approach here is pleasingly complete, with multiple lines of experimental evidence used to support their interpretations. The data are appropriately analyzed and interpreted.

The discussion of this paper is excellent - understated without obfuscation and complete without verbosity. This is a paper I expect to have a major impact on how we view the evolution of parental care, and to have an outsized effect on how others approach the mechanistic underpinnings of behavior as plastic and complex as parenting.

>> We thank the reviewer for their kind words and for their earlier feedback, which greatly helped us improve the Discussion of the manuscript.

Referee #4 (Remarks to the Author):

The revised manuscript is much improved. The revision is thorough and extensive, and the authors responded to most of the concerns raised in the review. Most important, they added a behavioral assay to show that larvae are not treated as food.

>> We are grateful to have been prompted to include the new behavioral assay, which we agree has strengthened the manuscript.

There are two other points that could be clarified in the final version. First, about the contradictory results from the two peptide screens: The authors explain this by saying that round 1 was a rough screen to narrow down the candidates, and round 2 is more reliable, suggesting that the round 1 signal could reflect variation due to noise. That could be true, but it doesn’t really resolve the contradiction or build confidence in the screen itself. If round 1 can produce signals that later flip or disappear by chance, then what justifies using it to narrow candidates? In this version the authors are asking the reader to trust round 2, and discount round 1, without showing why round 1 was informative at all rather than just unreliable.

>> We agree with the reviewer that our round 1 screening approach was “rough”, and we are forthcoming about this in the manuscript. The reason is that the ant genome simply encodes too many neuropeptides to allow simultaneous in-depth evaluation of all of them. Our round 2 screening approach, on the other hand, was thorough and robust, and we validated the effects of the two emerging candidates in additional experiments via various targeted methods.

Unfortunately, it is impossible in retrospect to say to what extent round 1 was informative in identifying candidates, given that we do not have a “ground truth” comparison. As the reviewer implies, and as we state in the manuscript, it is of course possible that additional neuropeptides that modulate alloparental behavior were missed by this approach. However, the important point is that this approach, in combination with the rigorous second round of screening, ultimately lead to the identification and detailed characterization of our two candidate neuropeptides.

We decided to retain the round 1 screening experiment in the supplement of the manuscript because this was in fact how we narrowed down our list of candidates. The alternative would have been to only present the round 2 screening results with a smaller set of initial candidates. However, we ultimately felt that omitting the round 1 experiment seemed disingenuous, and that it was appropriate to include it with explicit mention of the associated caveats.

Second, about manipulating only NPF and AstA in young workers. This would be sufficient if their goal is explicitly developmental, asking how these peptides regulate the nurse to forager transition. However, they don't say that in their response. Instead, they just argue that testing young workers is sufficient for their conclusions, without directly acknowledging the alternative possibility that peptide effects in older workers could differ in direction or be reversible. It would be good to state explicitly that they are testing only developmental function during the transition period, not reversible or state-dependent effects in foragers.

>> This is a fair point. We have now reworded the last sentence of that section of the results from "Together, these results show that shifting the balance of NPF and AstA in the brain drives age-appropriate responses to larvae." to "Together, these results show that shifting the balance of NPF and AstA in the brain drives the age-linked decrease in responsiveness to larvae as ants mature from nursing to foraging."

We have additionally reworded the last sentence (L. 224-228) of the opening paragraph of the discussion, changing: "These findings imply that, in addition to governing alloparental care, NPF and AstA also mediate age-related division of labor in the clonal raider ant." to "These findings imply that, in addition to governing alloparental care, NPF and AstA also mediate the age-related shift from nursing to foraging in the clonal raider ant. Since our experimental manipulations were performed in young workers, it remains unclear whether behavioral reversions in older workers could be underpinned by the same molecular mechanisms."

Referee #5 (Remarks to the Author):

I co-reviewed this manuscript with one of the reviewers who provided the listed reports.

Referee #1

I've reviewed the authors' response to my brood-care assay concerns and have read the revised submission. Unfortunately, the difficulty I have with the design of the assay and interpretation of results remains. I admire the authors' work and hope my comments will result in a qualification of the assay and interpretation of results in the paper.

>> We have added the following qualification of the assay, which should address the concerns of the reviewer (L. 64-68): "While this assay is simple and high throughput, it has limitations. First, because no food source is provided, and because *O. biroi* adults do not feed larvae via trophallaxis, our assay does not capture brood-care behaviors associated with larval feeding. Second, the short duration precludes assessment of the functional consequences of brood care on larval growth and survival"

The nursing assay (edited for brevity without changing content):
individual ants are placed into a narrow corridor along with a single larva and are filmed for 5 hours. As in colonies, young ants spend twice as much time in contact with larvae as old ants.

Comment: I may have missed it – where is this data presented?

>> This is in Fig. 1b (for the colony data) and Fig. 1e (for the one-on-one assay data).

Ants also exhibit the same nursing behaviors towards larvae in this assay as in colonies. Importantly, if presented with a food item instead of a brood item in this assay, both young and old ants exhibit qualitatively different behaviors, and the age-linked behavioral differences disappear. Finally, ants never consumed or killed larvae in this assay. Together, this shows that our behavioral assay captures core aspects of alloparental behavior, rather than feeding behavior.

Comment: Isn't feeding behavior part of larval care (see Snir et al. 2016, below, red highlight).

>> To address this comment, we have added the following sentence where we first introduce the assay (L. 65-68): "First, because no food source is provided, and because *O. biroi* adults do not feed larvae via trophallaxis, our assay does not capture brood-care behaviors associated with larval feeding." (See also our response to the reviewer's comment above).

Ants display a stereotypical two-phase response to larvae. In the first phase (P1), ants spend most of their time in direct physical contact with the larva. In the second phase (P2), ants tend to leave the larva for short bouts as they move around the corridor.

Comment: how is leaving larvae for short bouts an element of brood care"

>> The two phases describe how the ants behave towards the larvae rather than modes of brood-care. We do not define leaving the larvae as an element of brood-care beyond noting that, according to our definitions, in P2 ants perform less brood-care than in P1.

This two-phase response is specific toward larvae and depends on hexane-soluble larval compounds, which are often conspecific recognition cues in ants.

Author response (italics): The important point here is that an ant's tendency to perform all nursing-related tasks within a natural colony are highly correlated. They are correlated because most ant workers are either nurses or foragers.

Is there data (covariance analysis and/or transition probabilities) to demonstrate the association of nursing acts, or are the authors referring to this broadly for social insects, without specificity for the present case?

>> This is true for ants in general and likely follows from the tight connection between task specialization and space-use (Mersch *et al.,* 2016; Richardson *et al*., 2021). This pattern is also evident from the data presented in Fig. 1, where young ants spend more time in physical contact with larvae and also more time on specific sub-aspects of brood care.

Authors response: The elegance of our assay lies in the fact that it allows us to evaluate the ants' position on the nurse to forager spectrum by quantifying well-established aspects of nurse and forager behavior. However, it is evidently impossible (and unnecessary) to measure all aspects of the behavioral differences between these two states. This is what Referee #2 alludes to when they write that "Studies of behavioral genetics typically rely on an assay to reflect what can be a complex behavioral category (such as parenting) and I find that their assay is as good as any I've seen."

Authors refer to Reviewer 2's appraisal of the brood-care assay; I'm hoping that they will squarely address the issues I raised. I respectfully do not agree that the assay is "elegant" or "the best I've ever seen." There are for example straightforward assays of olfactory responses to brood as well as demonstrations of the effect of nursing and its effects on larval growth, as reported in Snir et al. 2016 and in other papers on ants. The frequencies of the most significant nursing behaviors can be quantified.

>> As we have described above, we have addressed the reviewer's concern by adding the following three sentences (L. 64-68): "While this assay is simple and high throughput, it has limitations. First, because no food source is provided, and because *O. biroi* adults do not feed larvae via trophallaxis, our assay does not capture brood-care behaviors associated with larval feeding. Second, the short duration precludes assessment of the functional consequences of brood care on larval growth and survival."

Authors response: Figure 1 shows that the behaviors captured in our assay constitute

core aspects of brood care and that our assay serves as a reliable and quantifiable proxy of nurse-like behavior.

The behaviors in the authors' assay record worker position, which is difficult to assess nursing function. Proximity is a useful but very general metric. Behaviors with measureable effects are better. Quantifying the most significant acts of nursing (see Snir et al.) provides convincing data.

>> This is a fair point and it should be addressed by the caveats now provided on L. 64-68 where we first introduce the assay.

Authors response: Ants frequently exhibit guarding behavior in laboratory colonies – naturally these workers would be the front-line if an intruder entered the nest

My point is that unless a quantifiable response to a stimulus is recorded, a stationary position cannot conclusively be considered "guarding" unless this interpretation of position is validated by other data, for example, data demonstrating that these ants are in fact "first responders" to a threat. The alternative is that they might actually flee and other workers provide defense. If a defensive contingency is presented, and workers indeed show defensive behavior, this result would be convincing, but in the absence of such data, the function of the "guarding" behavior is inconclusive and essentially has the same problem as any proximity metric. One could interpret such "guarding" behavior as simply inactivity, and I did not find data to refute this hypothesis.

>> This is a fair point, and we have included this caveat in our description of guarding behavior (L. 382-384): "guarding (standing over a larva and stationary, with the caveat that this posture has not been functionally linked to aggressive responses towards threats)".

Snir et al. 2016 present compelling descriptions of the fitness consequences of nursing that should form the basis of brood-care assays.

Quoting Snir et al. 2016, "In ants, for example, adults groom, transport and feed the larvae. One important type of social interaction is trophallaxis, the sharing of liquid food in the form of 'social fluids' either mouth-to-mouth or anus-to-mouth. Trophallaxis is widespread among eusocial insects and it is common in many ant species, both between adults and between adults and larvae. William M. Wheeler, who coined the term, even suggested that trophallaxis would be the key to understanding eusocial behaviour in insects. Far from being passive colony members, pupae thus have an active and central role in ant colony organization.

>> Snir *et al*. discusses ants in general – *O. biroi* do not engage in trophallaxis – we now make this point on L. 65. ("*O. biroi* adults do not feed larvae via trophallaxis").

We isolated pupae from the beginning of pupation until eclosion as adults, and found that they secrete a substantial amount of fluid towards the end of metamorphosis (Fig.

1a,b). Secretion
begins six days before eclosion, shortly after the pupae have melanized (Fig. 1a). The secretion initially accumulates in the exuvial space and then exudes from the rectal invagination of the pupal case (Extended Data Fig. 1), forming a stereotypical droplet on the abdominal tip
(Fig. 1b). Secretion continues until eclosion, ….. As long as we manually removed fluid daily, our social isolation protocol yielded high survival rates.

Adults consume the pupal fluid immediately as it is secreted, which is why the fluid does not accumulate and become visible in the colony context. Indeed, adult ants pile up the pupae and remain in prolonged physical contact with them, frequently touching the pupae with their mouthparts…… adults were highly attracted to the fluid and rapidly consumed it…..

… if we did not remove fluid from isolated pupae under clean rearing conditions, they drowned in their own secretion … pupae depend on adults to remove the secretion, and would otherwise die of fungal infections… This fluid is secreted in large volumes by all pupae during a specific window of development, and elicits parental care behaviour that is necessary for pupal survival.

……young larvae are usually attached to pupae, often with their mouthparts, suggesting that they consume the liquid directly as it appears on the pupal surface (Supplementary Video 4).
Larvae have limited mobility and are thus dependent on adults to place them on food sources. Observations suggest that O. biroi adults readily place young larvae on pupae and older larvae on prey items

When given pupae alone, ants placed almost all larvae on pupae until eclosion (Fig. 3d). When given a choice, ants showed a strong preference for placing the larvae on pupae as opposed to prey

Fig. 1 | Ant pupae secrete a social fluid that elicits parental care behavior that is crucial for pupal survival.

It is difficult to understand why the nursing behaviors reported in Snir et al. were not the basis of nursing assays and less-convincing assays whose results are not clearly interpretable were substituted.

>> The dynamics we described in Snir et al. occur during a ~1 week period where pupae and newly hatched (1$^{st}$ and 2$^{nd}$ instar) larvae overlap during the 5-week colony cycle. For the subsequent two weeks, the colonies comprise adults and larvae only. We now make this point where we introduce the assay (L. 56-59): “Naturally, *O. biroi* colonies oscillate between reproductive phases, where colonies comprise workers, eggs and pupae, and foraging phases, where colonies comprise workers and larvae. The composition of developmental stages in our assay thus represents colonies in the

foraging phase." In the Methods section, we clarify as follows: "Towards the end of the reproductive phase, eggs hatch into larvae. The foraging phase begins when larvae enter the 3rd instar ~1 week later, coinciding with the emergence of the previous cohort of callow workers. During the foraging phase, which lasts ~2 weeks, colonies are composed entirely of workers and larvae." In summary, the behavioral dynamics studied by Snir *et al.* do not occur during the periods of the colony cycle examined in the current study and are not relevant to our experiments.
