# Supplementary material for: Ancient feeding-related neuropeptides regulate alloparenting in ants
Source: Nature. 2026 Jul 8;656(8129):948–56. doi: 10.1038/s41586-026-10747-6 (PMC13518240; doi:10.1038/s41586-026-10747-6)
Supplement: Supplementary file 2 [file 41586_2026_10747_MOESM1_ESM.pdf]

nature portfolio

Corresponding author(s): Daniel Kronauer

Last updated by author(s): Apr 22 2026

Reporting Summary

Nature Portfolio wishes to improve the reproducibility of the work that we publish. This form provides structure for consistency and transparency in reporting. For further information on Nature Portfolio policies, see our Editorial Policies and the Editorial Policy Checklist.

Statistics

For all statistical analyses, confirm that the following items are present in the figure legend, table legend, main text, or Methods section.

n/a	Confirmed	
☐	☒	The exact sample size (*n*) for each experimental group/condition, given as a discrete number and unit of measurement
☐	☒	A statement on whether measurements were taken from distinct samples or whether the same sample was measured repeatedly
☐	☒	The statistical test(s) used AND whether they are one- or two-sided<br>*Only common tests should be described solely by name; describe more complex techniques in the Methods section.*
☒	☐	A description of all covariates tested
☐	☒	A description of any assumptions or corrections, such as tests of normality and adjustment for multiple comparisons
☐	☒	A full description of the statistical parameters including central tendency (e.g. means) or other basic estimates (e.g. regression coefficient) AND variation (e.g. standard deviation) or associated estimates of uncertainty (e.g. confidence intervals)
☐	☒	For null hypothesis testing, the test statistic (e.g. F, t, r) with confidence intervals, effect sizes, degrees of freedom and P value noted<br>*Give P values as exact values whenever suitable.*
☒	☐	For Bayesian analysis, information on the choice of priors and Markov chain Monte Carlo settings
☒	☐	For hierarchical and complex designs, identification of the appropriate level for tests and full reporting of outcomes
☐	☒	Estimates of effect sizes (e.g. Cohen's *d*, Pearson's *r*), indicating how they were calculated

Our web collection on statistics for biologists contains articles on many of the points above.

Software and code

Policy information about availability of computer code

Data collection	We used the open-source software anTraX (referenced in the main text) to track ant and larva (x,y) position coordinates. We used custom scripts to quantify and visualize behavioral tracking data. These scripts were written in Python v. 3.11.3, using Numpy v. 1.26.0, Pandas v. 2.2.0, Scipy v. 1.11.3, Matplotlib v. 3.8.0, Seaborn v. 0.13.1. Our custom scripts and example datasets are provided in a GitHub repository and the link to this repository is provided in the Methods. We used the open-source software Image) to quantify fluorescence intensity of confocal images of RNA FISH and peptide IHC stained brains.
Data analysis	We used the commercial software GraphPad Prism versions 8-10 for statistical analyses and graphing of all quantifications.

For manuscripts utilizing custom algorithms or software that are central to the research but not yet described in published literature, software must be made available to editors and reviewers. We strongly encourage code deposition in a community repository (e.g. GitHub). See the Nature Portfolio guidelines for submitting code & software for further information.

Data

Policy information about availability of data

All manuscripts must include a data availability statement. This statement should provide the following information, where applicable:

- Accession codes, unique identifiers, or web links for publicly available datasets
- A description of any restrictions on data availability
- For clinical datasets or third party data, please ensure that the statement adheres to our policy

All data are included with the publication as Supplementary Information. Code used for behavioral analyses and sample datasets is available on GitHub (https://github.com/Social-Evolution-and-Behavior/Paul_Kay_Kronauer_2026).

Research involving human participants, their data, or biological material

Policy information about studies with human participants or human data. See also policy information about sex, gender (identity/presentation), and sexual orientation and race, ethnicity and racism.

Reporting on sex and gender	n/a
Reporting on race, ethnicity, or other socially relevant groupings	n/a
Population characteristics	n/a
Recruitment	n/a
Ethics oversight	n/a

Note that full information on the approval of the study protocol must also be provided in the manuscript.

Field-specific reporting

Please select the one below that is the best fit for your research. If you are not sure, read the appropriate sections before making your selection.

☒ Life sciences ☐ Behavioural & social sciences ☐ Ecological, evolutionary & environmental sciences

For a reference copy of the document with all sections, see nature.com/documents/nr-reporting-summary-flat.pdf

Life sciences study design

All studies must disclose on these points even when the disclosure is negative.

Sample size	Sample sizes were determined by power analysis
Data exclusions	We excluded behavioral tracking data from ants that died or otherwise appeared unhealthy during the brood care assay. We analyzed all behavioral measures for outliers, and excluded outlier values from statistical analyses, as described in the Methods. Otherwise, no data were excluded from analyses.
Replication	We verified the reproducibility of the top half of the primary screening candidates in the secondary dose response screen. We replicated the NPF dose response experiment, as described in the text. The screening results for NPF and AstA were confirmed by additional experiments using peptide injection and two separate RNAi approaches. Due to feasibility constraints we did not replicate the experiments in Fig. 4 measuring differences in RNA and peptide levels with age, nor the nutritional status experiments in Fig. 6.
Randomization	Individuals were collected from a stock colony and distributed across experimental colonies/setups at random. The order of collection and place in which the colonies/setups were arranged was haphazard.
Blinding	Blinding to the age or treatment condition was used for analyses of brain fluorescence measures, and analyses were performed by two independent observers to minimize bias and check for concordance of results. Blinding was not used for behavioral analyses, because all analyses were done algorithmically using custom scripts and were therefore standardized across all experimental conditions. Blinding was not used for qPCR experiments as they involve standardized sample processing and instrument-based measurement.

Reporting for specific materials, systems and methods

We require information from authors about some types of materials, experimental systems and methods used in many studies. Here, indicate whether each material, system or method listed is relevant to your study. If you are not sure if a list item applies to your research, read the appropriate section before selecting a response.

Materials & experimental systems

n/a	Involved in the study
☐	☒ Antibodies
☒	☐ Eukaryotic cell lines
☒	☐ Palaeontology and archaeology
☐	☒ Animals and other organisms
☒	☐ Clinical data
☒	☐ Dual use research of concern
☒	☐ Plants

Methods

n/a	Involved in the study
☒	☐ ChIP-seq
☒	☐ Flow cytometry
☒	☐ MRI-based neuroimaging

Antibodies

Antibodies used

Rabbit-anti-AstA antibody from Jena Biosciences (anti-A-AST, cat.# ADB-062). Custom polyclonal chicken-anti-NPF antibody generated by YenZym (additional details in the Methods). Secondary donkey-anti-rabbit and donkey-anti-chicken antibodies conjugated to either a488 or a594 fluorophores (Jackson ImmunoResearch, codes: 711-545-152, 703-545-155, 711-585-152 and 703-585-155).

Validation

We validated the antibodies for NPF and AstA using two independent methods: dual RNA FISH plus IHC staining, and pre-adsorption with blocking peptides. These experiments are shown in Extended Data Figures 5 and 6, and details are described in the Methods.

Animals and other research organisms

Policy information about studies involving animals; ARRIVE guidelines recommended for reporting animal research, and Sex and Gender in Research

Laboratory animals

Clonal raider ant Ooceraea biroi. Female. Aged 0-6 months.

Wild animals

No wild animals were used in this study

Reporting on sex

All female.

Field-collected samples

No field collected samples were used in this study

Ethics oversight

Rockefeller University, NY, USA.

Note that full information on the approval of the study protocol must also be provided in the manuscript.

Plants

Seed stocks

n/a

Novel plant genotypes

n/a

Authentication

n/a
