# Supplementary material for: Ancient feeding-related neuropeptides regulate alloparenting in ants
Source: Nature. 2026 Jul 8;656(8129):948–56. doi: 10.1038/s41586-026-10747-6 (PMC13518240; doi:10.1038/s41586-026-10747-6)
Supplement: Supplementary file 3 [file 41586_2026_10747_MOESM3_ESM.docx]

**Supplementary Table 2 | Translated and annotated *O. biroi* neuropeptide precursor gene sequences.**

For each record, the *O. biroi* neuropeptide precursor gene name (including parenthetical alternate names found in the literature), protein accession number, and precursor protein sequence is shown. The region highlighted in light gray is the signal peptide (determined with Signal-P6.0), magenta highlights the neuropeptide sequence(s), blue highlights the amidation signal(s), if any, and red highlights the protease cleavage recognition sites.

Hypertrehalosaemic prohormone (Adipokinetic hormone / AKH): EZA51391

LFPFFSRFRKMPVSLFAVTFLFLILISVGEAQLNFSTGWGQGKRSSDMRIKSSNTDCSSQNTSLEQLLKLYTFIQIEAQRILDCENLNK

Allatostatin A: XP_011344812

MNSRTSLTVNSIIIFCLLCVVGKSIAAMEEAPSSPLHSPRLNPLLNNLKFEEPSEKRAPYTYTVSGYKRLPLYNFGLGKRWVDNSEDKRTWPFSFGIGKRLRDYNFGLGKRNNGYHPLNLDYFSADNLESYHSREDGSDDFMEDKRGIKPFSFGLGKRAWKLATGESPASGKEANDVVGPKYLLSLGKGMIEDEELAE

Allatostatin C (Prohormone1): XP_011338324

MMLSVRSAIALALVLLVLAEWSAAMPATDKDKERLLNTVDLIDDDGSIETALINYLFTKQIVKRLRNQLDIGDLQRKRSYWKQCAFNAVSCFGK

uncharacterized LOC105287280 (Allatostatin CC): XP_011351133

MGISIIPLIVVAMVPIIVGDFGQRVIGQRSDYPFDDCLDYCADEHPTVKKKSTLLVDQLLLALQRAAQKDVIRGKGIDRSASVLSARSRMGPDDDQLKGMQRRGQTKGQIYWRCYFNPVACFKRK

Allatotropin: XP_011348251 (signal peptide not detected)

MIDQFERLSGVLRRRQEIMRAVLAIALVLAVGLVMVTTATKAHNYPRFFKGHRAKLREIRGFKPEYLSTAIGFGKRVGPAEAQDLSARERILLSLLGNFPREMPAEVLLRTMQANPALANRLTQLVAQNDQGDEEPPMMEHPKLERTLALI

Bursicon subunit alpha: XP_011331715 (low quality protein) (signal peptide not detected)

MCQKAFSYPKEGRKHGHLWCIKAYDTDLTSTVKCXERTAMYRSRSRLFHYFVINGLLMCLLYGGVTAIIGVDECQAMRVIHFLQYPGCVPKPIPSYACKGRCSSYLQVSGSKMWQMERSCMCCQESGEREASVSLFCPRAKPGEKKFRKVITKAPLECMCRPCTSVEEYAIVPQEIAGFADEGPFATSAHFRRSSGLE

Partner of Bursicon (Bursicon subunit beta): XP_011331713

MPLRQQGIKSTTPGFYTLDSCDAEISDDENCETLQSEIRITKDEYDEIGRVRRVCSGDISVTKCEGFCNSQVQPSVVTTTGFLKECYCCRESYLKERFVVLNQCYDADGIKLIGTEDETMEIKLREPAECKCIKCGDLA

CAPA peptides (CAPA Periviscerokinins): XP_011333465

MQHNRFFILVVLLVFSTSFNLARCLVGQHFQSAREAQKLKVNDRRSAGLVPYPRIGRNSEMATLPRSERALGMIHQPRIGRSDSLGNLNRFHDLSADDIEFYIMHDMDPDLLDLDYEEYANRPIAFKHTDKMQKDGSWLVPDHVHGYKDHHFAQKIDYPRSYYSALRDSRNTQGQGGYTPRLGRENEHDTASFL

Cardioactive peptide-like (CCAP): XP_026827340

MKITEYLCCVLTCMCLLKVANTEVSKDKNSLLQLERQLLDALTSEKTVKPKRPFCNAFTGCGRFFPDEKRSFVITRRDSQDFQATSNSVRLPISLYKALLNAAKENTWNKIGREENNYRLQQMPQAYLLDRVPLRDTLES

CCHamide-1 (CCHamideA): XP_011344844

MALALRNPRTLTIVQTCIIMLIFCFAGCAAGSCLSYGHSCWGAHGKRSEDQVGATMRILAAKTSSNGSPHNDVVPSSKTQWILSRLIAGQPVLPSSDKYRVRWSDFTKDKDVYASPKWEYNVAPINDESIEPMRIPINNESNEKKINDAQGIMRTVNEKLGNVPEIVLVSSNGYDKPINGFRKLDVLELLVRLVILCSYSFDLLIIFLINFRVKNYLTGITMRLISE

Neuropeptide CCHamide-2 (CCHamideB): XP_011344853

MKIVTSSLTFSFYIAIALIILVSVSDKAHAKPWAEEHIDRKSDKQIRGYVRKGGCASFGHSCFGGHGKRFDSAMQQNMLQASNRRNFQESEVSSPNDELFYVLPSDGFRERSEQAFMRARKDTQQQFDLNPLSSLVNQWIASHRRSHRTDSDVTNK

uncharacterized LOC105279907 (CNMamide): XP_011338330

MTERASQQTAARTSLCLLAALVLSCASGSKKVLAQPIEPLAPGFVYGDNFADDPESLMLLNRLKQLTELKHRVVEKEHEITDVQIAIQAMLEAKAQERERTISPDYSEPEALPVPAAMIHHGNQQHPGKRQGTNAVSYMSLCHFKICNMGRKRQSK

Corazonin: XP_011339019

MTSRRVLVFLVLLLLVNAAICQTFQYSRGWTNGKRSEFPSAADERFTNGDLKRLKMLVHGNADEQPLLFHCDFVDKRKLVHADNYAPRLRHEKEQNDDNY

Diuretic hormone class 2 (DH31 / DH2 / Calcitonin-like diuretic hormone): XP_011331351

MQPKMTVLCTLLAFIVVVAVSSLTADAMPHSHESYWDQQDDIDRDEFLEILSRLSRTVMNRPEMENSKRGLDLGLSRGFSGSQAAKHLMGLAAANYAGGPGRRRRSEQA

Diuretic hormone 44 (DH44 / CRF-like diuretic hormone): XP_011335082

MAMNVINLHACTMVLIGILMLTTTINLISSAPLSPYEKRELSDRRPKIFLLPDRAMPELESELLETDNNFGTLRAKRIGSLSIVNNLDVLRQKFLLELARRKALQDQRQINENRLVLDSIGKRSVPEFYETDAASFVKPGTRNGHDWTAPSNRLEWIEEAPGPQDDRMAKVQANELRL

Ecdysis triggering hormone: XP_026827105 (signal peptide not detected)

MMLQRAYIQQIGGVFFNAFLVGMVTLLICENITKADEVPAFFLKIAKIPTLPRVGRSDRFEDFFYKAEKHIPRIGRNSQQDETYTDLTKRRIDYPPKVDGWSSQNFPLAIEGPRELWRTLAGYSRDGSDDIENEVWHRKKRTETEPITTQEK

Eclosion hormone: XP_026827607

MNLSNRLILLLAILCLSIHMTVAERNTGCLNLAVCHMPISLNHFTHNINISDNNRRGWTTFTKFVTKKGICIRNCAQCQIMLGGYFMGEKCANFCVKHKGKVIPDCEDEFSIRPFLQKAPENEY

uncharacterized LOC105280686 (Elevenin): XP_011339702

MTTAFLQRSIIIFAILLCCFQSWVDAVDCELFPYHSSCRGMMTKKRAFNRGHFIESEDSRVPRMRYLIALLDNAHKIKHERQAKRMLDDMPEEEIVYHDY

uncharacterized LOC105274583 (Flitkin): XP_026826695

MASQCWLLPLLILGTSAIALVPAARQSNQESLRSALDAVARKQRFLDETETPEYYNELHSFKYHGGDADRKFDRDFDEDDEEFLNEDNGQPETMGEDIQGLNNKLFERALHDYLEDLPQQEEPVASIFRERELQRSSSRKRGGAGDRSNLDDKDLARLFVEELQDSSPYSDRSRLLEEMDDDDYLTALQTLYDRYRAGRGNKVYETAGPMSWGELLSKSLPPRGQTKGGNEEFMHHDQQNLLYPSLAQRRNVNGRYPIGRELGSYRGLAKRYLVAKRSPTQTKLKTTDPKVAQDLGALFGTQSTDIHNHTHKSDHEHDHKHDDGHDHGHDHDHNHEHDHDHGHENSSEALLKVTPSSKDQKENDTKAAKSKFLQVRKKSVDWSQYFGIDRRKKKATFMAGQGTQNQDDEWMLQRYYENMAENFKPKDDENERKDKLEQMDSKLKYIKDLIIEEALKYANLEDEADLQKVKDKLISRMAAAYSLQKMRKALGDLRDNVAALKEAQKTQKEGQNFTSNFRENNANNANNGAGNKVNDKRSDTANNLDDGEFIEEPRSCPELDAIEWRCRTVDNLAGDTSRMLYGHCVKLQICKACAQDEETLLPCLANYAVEAGRVCDALDSRERLPAGSRLAAARDEEKQSCANAALLLSQLQPPTAASVQCRVQSPRDSCLRHYYARYEHRYLRTSPVYGDGRARLHHAGALDVLQQTMSER

FMRFamide: XP_026829891 (signal peptide not detected)

MCALRVLSLLYKGCHLQPRHPLHHQPPPTRRKTPTVATFATVCALFPFFFLSISASAFVASCVSLRGKKTESRQYARFYSQIAVWTFNAMAILCNWALVSSSVLIPPKDESSLRIFKEPSNEFEYVAKRHGIDVRKEDADSKERRSSMSSSFIRFGRSQSAFNTDNSDFDSEADSKVSRYPRWKSPDIVIRFGRSSFKTANVEQLKRGRNDNFIRFGRNIQIAPADVDLSTVCSALTLNGVSSDAELHPDGLRLLHLCNSLNKIAGEISLDTFEDQAVSRHRE

Insulin-like growth factor 1 (ILP1): XP_011344802

MRHVMNKTKTVLLLALLLLNVLLAAGYVPYKKSHLKMQKLCSKSLSDALYVVCRESGYNEPFSYSGEDEPRADTGPGLVEECCYHSCSYDQLTQYCKPLPEEKRADSRDVIGESNVANLPYSTSTRDLSSEQNPPLEMDHVGGAIKRKVDDLKRGRHRGKGGRNDDECRGKADAKKRHRGRHCRCRHRRLQCRRASKVSHGNVKPLVNKFTTAPPTQNTTSFPVTS

Insulin-like peptide 2 (LIRP / ILP2): XP_011335977 (low quality protein)

(the two indicated sequences are joined by 3 disulfide bonds to form mature ILP2)

MRETSPVAVTIIIIIVVVVVVVVSPVVTPVRSASSETECQTVILPRVIERSPCDPPCFTSMYSMFANRLNVVVTLVFAIAFLVAESGNAQVDGFPQFNTKRSSISAPQRYCGKKLSNALQIVCDGVYNSMFKKSGQEMELADYPFPYESFPFVPPERANGMLDRTSARFRRSRGIHEECCVNACTISELSSYCGP

Neurophysin 1 (Inotocin): XP_011329433

MLKKLVVFATLIFLSYACLITNCPRGGKRGDIAPSLGTVARECPSCGPNHLGQCFGPHICCGPSIGCFIGTSETYRCRKESLYTRPCVAGYAMCRGNTARCATNGICCSQESCHMDTSCRISDVGNDRKIDADLNVMFPGNEASSETFP

Ion transport peptide: XP_011334559 (signal peptide not detected)

MMHRQQNTQSSPSNDEFAMYPSAAYYSSCSTLPSSTSTSRSSSPASSCPLLMSVLTWSLMLLLISSCIGLGADAATLNGHPLGKRSFFDIQCKGVYDKSIFARLDRICEDCYNLFREPQLHMLCKKECFTTDYFKGCLDVLLLTDEVGKIQMWIKQLHGADPGV

Invertebrate parathyroid hormone 1: XP_011343552

MNLSVSAAILCVLVVALLPETTYARPPHWLLNRHRRVSDQRLAEIETMLGLEKVKGVAVPVGLGKIDPAIIGRKRRSIPESDLAKPQGPSMLINPDVDSAPGQSGQVPLLWKKDPREKQRNYQLI

Invertebrate parathyroid hormone 2: XP_019888401

MQRTLMVIWILVALTLPGFIHGRPAADDGRSVLSMFTAHASKLEDVLDYEQKPTSLFAQGGSLVPTKLRVNRSSDHRLSELETLMTMSKINSKRSPLPTGQASRQLDPARIGRRKRFIEKVPFEVIPIHQHTDRPKDEGNSAYSLIRYILSHQLHSITINHTSNFRGLHIIVSLYNRTYRQKSGGRTCMFKAIH

Myosuppressin: XP_011336204

MMSSTLIIVISVTAMTILSGEAFAALPAQCNSGFLEELPPRLRKICVAIARIWDVRDMNDFVDDKEYRENLPRYDSGVKRQDVDHVFLRFGKR

Queen brain-selective protein-1 (Neuroparsin A): XP_011333498

MLTFQFSQAIVLSAAIFLVCAHPTRRQEVRPLCVGCGDECDKCEFGFTISALCGIPECRRGPGQICGGPSEAWGVCGDGLICNCNRCAGCSLASLECFTNPCLPHQSLESRGHLELLDRYSPVDRVAK

Pro-neuropeptdie Y-like (Neuropeptide F / NPF): XP_026826397 (the underlined amino acid is corrected vs reference sequence)*

MRTEMSAARFLCCLLLIAIVGTVIACAEPESMARSTRPKVIANSEELKRYLDLVREYYSMTGTARFGKRNDPPISENTIWEIFRMILDNAQRQNEQRRRDRIRQNKEPVPFNDF

*The annotated protein at XP_026826397 for NPY-like has an ambiguous amino acid in the precursor protein due to a genome assembly error we discovered at the NPY-like locus. The sequence provided above is corrected based on our findings, as discussed in the Methods.

Neuropeptide-like-precursor 1: XP_011333909

MDFSRSYLLSLLFYVFIINEIHQPTVTCLDEEGTQQCIPKKIFMELIRLPEFSSGIAAYMRTTRVAQRTKGYNDIDHLKVQFSSPENDDAEICIPASVYLELLRDPALRGYLSVMARTHKLLEFPGSLLNSSEEADSRELTREAEKRSLATLAKNGDLPSLPIATQEREGDGDDEEKRSDLSNDNVNGLMPILMDDDRRRMPEVYDYLPVDLDPEVPEYSLDKRNVGSLARDFALPTGRRHIGSVVRDYGLPSGKRNIGSLARQSMLPSGGKRNVASLARYYLLPQAGKRNVAALARDASLPYGKRYLGSLIRSSGYPVRGYDEGKRSVASLARNADWPFLAKRGGTAGGKMIVLGMLNRHGRSLNDDREVHNEPLDLQQLIRQNGDQIKEDVPQWWAIPSMSESLDETKTRNRSNRRIDGASQTRYKRQIDFSDEYPLSVMQNTNMLEYEDMIESFPDHYPEKRFMGSAPEMPADSAGYPEVLRASKRHIGALARLGWLPSFRAPRFSRSPRENRYLVERENPADGSSSNYTPNSSTRSLRPMFTLKTRLQSLRGDCHQGFKRFLLLPDADNFLRTPNSHIMPRSM

Orcokinin peptides: EZA55422

MNVRPGNFDEIDRSVFDRFTKRNIDEIDTAFDSFFKRNFDEIDRVGWSGFVKRLDNYLADRQRR

Pigment dispersing factor (Pigment dispersing hormone): XP_026828115

MANYARYVVAIVVVIVGVVCGQTFNPLEDTDRNILGLNLPYGRGLDSELQLARLLLVTPRMCHSKRNSELINSLLGLPKNMHNAGK

Prohormone-2: XP_011336369

MTYNWAWLLLFSLVSIGTCLPIEDVKKVDQVMKPKSKRAQEMLMFGNQQNRRANAASESFTSNAEKRTLASSGLGSLKAALAEETNSKPTQPKSSSSNAMYEQETYAPYDASYDYGKVLVNEVEEDGPRVWDVPPYSRYYTSEDRRKRSEKSTATARPATTLQPPASSYQTPTPTQQPVQAQVKRSIPFYQEPRYKRELPLDIDPEDVLAILSLWENERRNGNWRKYANEEYENIMDDNGLLADEEDPRNALPWLDTPVYPSRHYAADTLSPSDIGIVRTHPPSYYEQLGNQLDQQYGGTTQYGNPQYGSSQYGNSQYGFVYPQRAYYPQEKRFMVSKKRSQSYDPYSAAQLQLSSQPRSYQYPHRVVY

ITG-like peptide (Prohormone-3): XP_026828232

MKACTAITLVLVANTAYIGVEAWGGLFNRFSPEMLSNLGYGGHGGYINRPGLLQEGYGGIYGEAAEPTEEPCYERKCVYNDHCCPGAICVNVDGVVGTCVYDFGMKQGELCRRDSDCETGLMCAEMPGRETKSCQPPTTSNKLYNEECTMSSECDISRGLCCQLQRRHRQTPRKVCSYFKDPLVCIGPVATDQIKSVIQYTSGEKRITGQGSRLFKRMPFA

IDLSRF-like peptide (Prohormone-4): XP_011350282

MVRGFCNGAVALGIALTACAAFPRAVMAIDLSRFYDHFNTKRSDACHPYEPFKCPGDGACISIQYLCDGAPDCQDGYDEDSRLCTAAKRPPVEETASFLQSLLASHGPNYLEKLFGTKARDTLKPLGGVEKVAIALSESQTIEDFGAALHLMRSDLEHLRSVFMAVENGDLGMLKSIGIKDSELGDVKFFLEKLVKTGFLD

Prothoracicotropic hormone (PTTH): XP_026831335

MLETSPQCTTMARARRVLGRMKLLILCVMVDGLLAEDPGQVLSIAQWKEQVVAPEFLLDDREDISGSRNSFFYEDKRSFRPEGIGEQTKRVTGADDVDLQQPRLATRSLQCTCETQYEIRDLGEGHYPRYLTASRCKPKACQSKFNSCRLLYYMVHILSQRDLNGLSEDRFSDDSALPETPLPEALRHKWQLKPMKIPVACVPATR

PBAN-type neuropeptides (Pyrokinins): XP_011330631

MVEMVVIGNHISRGTSVCILAMVLCLASRASGEYESREMSNGGPNDRSSSNDFSSCTEGKCVKRTMKEITNGMWFGPRLGRRRRSEVDKQEINSEIEALANVLGNERWAFITIPASEKRQPTQFTPRLGRGSEEDPFSYADAIDRYEFEESDQPLPPIFAPRLGRRLPWAPSPRLGRQLRGAMRKI

Short neuropeptide F (sNPF): XP_011340543

MYAKRCAAFILLVIAGLVSATENYMDYGEEMAEKSPAENIHELYRLLLQRNALENALGGIPLEHLMIRKSQRSPSLRLRFGRSGPHVSAGGLPRPLGAATAAGYDNN

SIFamide: XP_011342125

MISIRFTIALAVIIAILALSVDAAYRKPPFNGSIFGKRSNTVTDYEVTSRALSAICEVASETCTAWQSRQESN

Drosulfakinin (Sulfakinin): XP_026831426

MAPMRRPSLSSSVHLQTLTRDYRLRHLETEKNMNQFFMLFYIMMVMWMFYGKCKAAPGFIGIHRNLENYPYVHRYALESLMNDFAEDLVGEDEDNLQLSKRQQLDDYGHMRFGRRSLGDDIGYGHL

Tachykinins: XP_011347699

MIISSILFLAIWTSSSFAEESGDVASVKRAPMGFQGMRGKKDPLILTSSEHDNELSKRTLMDFQDMHKKDASAPEIEDNLLHEEFEKRAPMGFQGMRGKKDYLLPDFEDAYFRDDYEKRAPMGFQGMRGKKTLLEEEYYKRAPMGFQGMRGKKSLEEVLGEIEKRAAMGFQGTRGKKTYIFEYPQGYEKRLLAMGFQGMRGKKDEIPTEWEKRAPMGFQGMRGKKTLLDEIEELEKRAIMGFQGTRGKKDGYENYIDYYMDPMDFDKRAPMGFQGMRGKKDSDKRAPMGFQGMRGKRNMAQRFGTDADFETRSSIEYQGTGDRRNALALCQVEKRSPFRYFEMRGKKNPRWELRGMFVGVRGKKWAPAPYEDRSPFISVLDNTGRTGVVDGDSSAALDSQ

uncharacterized LOC113562312 (Trissin): XP_026827162

MNRTWMLLFLAACALWTFGEAIPCDQCGRECADICGTRQFRACCFNNMRKRHSDTLGLKIWMRPRRYAFNLYPIYDA
