# Supplementary material for: Ancient feeding-related neuropeptides regulate alloparenting in ants
Source: Nature. 2026 Jul 8;656(8129):948–56. doi: 10.1038/s41586-026-10747-6 (PMC13518240; doi:10.1038/s41586-026-10747-6)
Supplement: Supplementary file 4 [file 41586_2026_10747_MOESM7_ESM.docx]

**Supplementary Table 6 | Sequences used for generation dsRNA and siRNA.** Sequences of the cloned AstA, NPF, and GFP fragments used for generating the dsRNA, and also the siRNA duplex sequences.

> AstA dsRNA sequence used for RNAi (566bp)

GCATCATTATATTCTGTCTGCTGTGCGTCGTCGGGAAATCAATAGCGGCGATGGAAGAGGCACCGTCTTCTCCTTTACACAGTCCGCGATTGAATCCATTATTGAACAATCTGAAATTCGAGGAACCTTCTGAAAAAAGGGCTCCGTATACTTACACCGTTTCCGGGTACAAAAGGCTACCTCTGTACAACTTTGGTCTCGGAAAACGATGGGTCGACAATAGCGAGGATAAAAGAACGTGGCCGTTCTCGTTCGGCATCGGCAAGCGCCTGCGGGATTACAATTTCGGCCTAGGAAAACGCAATAACGGATACCACCCTCTGAACCTGGACTACTTTTCGGCCGACAATCTGGAGAGTTACCATTCTCGTGAGGACGGCTCGGACGATTTTATGGAGGACAAACGCGGCATCAAACCATTCAGCTTCGGCCTCGGGAAACGAGCCTGGAAGTTGGCGACGGGAGAATCGCCAGCGTCTGGAAGAGAAGCGAACGACGTCGTTGGCCCGAAATACTTGCTCAGCCTGGGCAAGGGAATGATCGAGGATGAAGAGCTGGCCGAATAG

> AstA DsiRNA sequences (short oligos that duplex to dsRNA used for RNAi) (27bp)

5'--- GAUGUGCUAAAGAAUCAUAUUUACC -3'

3'- AGCUACACGAUUUCUUAGUAUAAAUGG -5'

(Duplex MW 16523.0)

> AstA coding sequence (579bp)

ATGAATTCAAGAACAAGTCTGACTGTTAACAGCATCATTATATTCTGTCTGCTGTGCGTCGTCGGGAAATCAATAGCGGCGATGGAAGAGGCACCGTCTTCTCCTTTACACAGTCCGCGATTGAATCCATTATTGAACAATCTGAAATTCGAGGAACCTTCTGAAAAAAGGGCTCCGTATACTTACACCGTTTCCGGGTACAAAAGGCTACCTCTGTACAACTTTGGTCTCGGAAAACGATGGGTCGACAATAGCGAGGATAAAAGAACGTGGCCGTTCTCGTTCGGCATCGGCAAGCGCCTGCGGGATTACAATTTCGGCCTAGGAAAACGCAATAACGGATACCACCCTCTGAACCTGGACTACTTTTCGGCCGACAATCTGGAGAGTTACCATTCTCGTGAGGACGGCTCGGACGATTTTATGGAGGACAAACGCGGCATCAAACCATTCAGCTTCGGCCTCGGGAAACGAGCCTGGAAGTTGGCGACGGGAGAATCGCCAGCGTCTGGAAAAGAAGCGAACGACGTCGTTGGCCCGAAATACTTGCTCAGCCTGGGCAAGGGAATGATCGAGGATGAAGAGCTGGCCGAATAG

> AstA mRNA sequence (includes 5' and 3' UTR) (2329bp)

AGGCCGGCGTCGCGACGCCCTCGTCGTCATTCCCGCCAGCAGACACCGAAGTCGCGGTACCCGGCGTTGAACGTCGCATCCTCACGTGAGAACCAGAACGGAAGTGACGGTCTCGCTTGTGCGAACGGAAGTTTCATCCCTCGGAAGAAAGAAAAGTAACGCGGCGGGGAGTTCCCTTCCGTCTTTTGGCAGACCGTGAAGACGTCGCGTACCCTCACCTCACCTGCAGGATTGTGCCGGTAGTTGTTCAGCAACAGATTCTAACCGGCATCATGAATTCAAGAACAAGTCTGACTGTTAACAGCATCATTATATTCTGTCTGCTGTGCGTCGTCGGGAAATCAATAGCGGCGATGGAAGAGGCACCGTCTTCTCCTTTACACAGTCCGCGATTGAATCCATTATTGAACAATCTGAAATTCGAGGAACCTTCTGAAAAAAGGGCTCCGTATACTTACACCGTTTCCGGGTACAAAAGGCTACCTCTGTACAACTTTGGTCTCGGAAAACGATGGGTCGACAATAGCGAGGATAAAAGAACGTGGCCGTTCTCGTTCGGCATCGGCAAGCGCCTGCGGGATTACAATTTCGGCCTAGGAAAACGCAATAACGGATACCACCCTCTGAACCTGGACTACTTTTCGGCCGACAATCTGGAGAGTTACCATTCTCGTGAGGACGGCTCGGACGATTTTATGGAGGACAAACGCGGCATCAAACCATTCAGCTTCGGCCTCGGGAAACGAGCCTGGAAGTTGGCGACGGGAGAATCGCCAGCGTCTGGAAAAGAAGCGAACGACGTCGTTGGCCCGAAATACTTGCTCAGCCTGGGCAAGGGAATGATCGAGGATGAAGAGCTGGCCGAATAGAATTCCTCCCCGTCGGCCACAGCACGGACACTATCCTTTTACCCCTCGTTCCCTTCAACCCCTCGGACGATCACAGGAAACGCATCACTTATATGTGTAACACTACTTCCGAAAACTCCGACTGAACTCGAACTAAGGAGAATGTATAAAATTTCATTACTTTCTTGACATTCCGTCGTTCGTCTTTAATAAGTATCTTATGATCTCTATGTCGTGCAGAAACAAATTAATTATTGATTAATCCGATTCGTTCCTTGATAGAATCACTCTATGATAAATTACTCTTCTCTTTCTGGCGAACTCGGGGTTAAATTCATTGAAATTGCGGAACGCAATTTCTGTGATAAGTATCTGTGTTAACAGCACATGTATGATATCAGCGAACGCCCTTCAGAGAGAGAGAGAGAGAGAGAGAGAGAGAGAGCGAACCGTATCGTTCGTGGCATCGTTAACAGCGATTCCGAGAATCTCTTTTTTCGAAAATTTCCTGTAAAATTCATGTGTCATCGGCAATCCGCGCTCCTCTTGCACATGTCTGGAAACCGATGAGGGCACTTTAGCGAAGAGGTATCTGATGTAGTAGTAGAGAAATTCCTCGAATCACACTTGTCCGATGCTCTGTCCGATTTGTTTGTTCTCAATTCCTGTAAATTAATAATTATTTTGATCAGAGTGTTAAATCGTAATTTAACGAATCGTTACAAAACAATATCTCGATGTGCTAAAGAATCATATTTACCTGCGTAACCGCGGCGGTACTATACTCATTCTTATTGCTCGACAATTTAATCCTTGTAAAAAGAAGAAGCAGAAATTATTCTACGCATAGTATAATTTTTTTCTTTTTCTTACAATAATTAAATTGTCGAACAACAAACATCAATTTAGCTTCCCATGCATACTTTGAATCTGAAATAAATAATTGTTAACAAAATTTAAAGATCTCGAATTTTCATCTTTAATTTTTAATTCTTATGGATGTACTTTAGACACATTGACATAAAATTTTCATTTTGTAAATGTATTTGAGACATGCATTAATGATTCTTCAATTACAATAATGATAATCGCAAACTCGCTGAGTCCTGTTGAGCCCTCTCAGCGAGAGGGTTGCAACGCGAATTAATAATAACACGCTTGCTATTATCTGTAGCACATTCACCACAATCTAGACATTGAATGTACAGGACGTCCCGTATAATATTCTTTATTTAACACTTCCTCAAATCTCACAATCCTCAAGCCTCGCTCACTTGATCTACTGATCAGCAGTGCTCTGCTGCCAGTTATCTTTTACCGTGTATGTCTCCATAATGTCTCCCGCTCGTTCGTCATCTCGTGATGACAAAACATCACAGTGCAGACGTATATCAATCTCTCACTTCCACAATTCCTGTATTTTAATGCAAATGCGCATGAGCATTGATACCAAAGAATTAAAAATAAACATTTTTAAGAGT

> AstA protein (199aa)

MNSRTSLTVNSIIIFCLLCVVGKSIAAMEEAPSSPLHSPRLNPLLNNLKFEEPSEKRAPYTYTVSGYKRLPLYNFGLGKRWVDNSEDKRTWPFSFGIGKRLRDYNFGLGKRNNGYHPLNLDYFSADNLESYHSREDGSDDFMEDKRGIKPFSFGLGKRAWKLATGESPASGKEANDVVGPKYLLSLGKGMIEDEELAE*

> NPY coding sequence (345bp) > This is also the sequence used for dsRNA RNAi.

ATGCGGACAGAGATGAGTGCAGCTCGTTTTCTTTGTTGCTTACTGTTGATCGCCATTGTGGGAACTGTCATCGCATGTGCCGAGCCGGAATCCATGGCTAGATCGACGAGACCAAAAGTAATTGCTAATTCCGAAGAATTGAAGCGATATCTCGATCTTGTTCGAGAGTATTATTCCATGACAGGAACAGCAAGGTTTGGAAAGCGAAATGATCCACCAATCTCGGAGAATACTATTTGGGAAATATTTAGGATGATCCTGGACAATGCACAACGGCAAAATGAACAGCGAAGACGGGATAGGATCAGACAGAACAAAGAGCCAGTCCCGTTTAATGACTTCTAG

> NPY mRNA sequence (includes 5' and 3' UTR) (696bp)

GATCCGAACTACTGAAACGTTCGCCGGTACGTCAGGAAGATATACCAGAAAGCTGCCGACAGGTGAGGAAGAAATCGTCTATCAGACGGACTCGGATTGCTCTTAAGCCGATCTTCCCCCTTATTCGCGGTTTGCCATTCGATCTACGATCCGATCGTCAGGGAGATTTAATCCAACCGAGACTGCTACCTCAAGCCGTTCAAAATGCGGACAGAGATGAGTGCAGCTCGTTTTCTTTGTTGCTTACTGTTGATCGCCATTGTGGGAACTGTCATCGCATGTGCCGAGCCGGAATCCATGGCTAGATCGACGAGACCAAAAGTAATTGCTAATTCCGAAGAATTGAAGCGATATCTCGATCTTGTTCGAGAGTATTATTCCATGACAGGAACAGCAAGGTTTGGAAAGCGAAATGATCCACCAATCTCGGAGAATACTATTTGGGAAATATTTAGGATGATCCTGGACAATGCACAACGGCAAAATGAACAGCGAAGACGGGATAGGATCAGACAGAACAAAGAGCCAGTCCCGTTTAATGACTTCTAGAGTTTGGATGCTGACAAGTACACGTCGCTACACGACCTGGCTCTCATCCGAATGTAATTGACAAATACTTGGACAATGTACAATAAATTTCATAGCGTTTTCCCTTTTACTCTCCCTAATTTCCATTGAAATATATCAAAGATGTTA

> NPY protein sequence (115aa)

MRTEMSAARFLCCLLLIAIVGTVIACAEPESMARSTRPKVIANSEELKRYLDLVREYYSMTGTARFGKRNDPPISENTIWEIFRMILDNAQRQNEQRRRDRIRQNKEPVPFNDF*

> GFP dsRNA sequence used for RNAi (546bp)

ACGTAAACGGCCACAAGTTCAGCGTGTCCGGCGAGGGCGAGGGCGATGCCACCTACGGCAAGCTGACCCTGAAGTTCATCTGCACCACCGGCAAGCTGCCCGTGCCCTGGCCCACCCTCGTGACCACCCTGACCTACGGCGTGCAGTGCTTCAGCCGCTACCCCGACCACATGAAGCAGCACGACTTCTTCAAGTCCGCCATGCCCGAAGGCTACGTCCAGGAGCGCACCATCTTCTTCAAGGACGACGGCAACTACAAGACCCGCGCCGAGGTGAAGTTCGAGGGCGACACCCTGGTGAACCGCATCGAGCTGAAGGGCATCGACTTCAAGGAGGACGGCAACATCCTGGGGCACAAGCTGGAGTACAACTACAACAGCCACAACGTCTATATCATGGCCGACAAGCAGAAGAACGGCATCAAGGTGAACTTCAAGATCCGCCACAACATCGAGGACGGCAGCGTGCAGCTCGCCGACCACTACCAGCAGAACACCCCCATCGGCGACGGCCCCGTGCTGCTGCCCGACAACCACTACCTGAGCA

>GFP DsiRNA used for RNAi (27bp) sequence unclear.

EGFP-S1 Positive Control DsiRNA. IDT catalog #: 51-01-05-07

Have to reach out to company for sequence.

>GFP coding sequence (720bp)

ATGGTGAGCAAGGGCGAGGAGCTGTTCACCGGGGTGGTGCCCATCCTGGTCGAGCTGGACGGCGACGTAAACGGCCACAAGTTCAGCGTGTCCGGCGAGGGCGAGGGCGATGCCACCTACGGCAAGCTGACCCTGAAGTTCATCTGCACCACCGGCAAGCTGCCCGTGCCCTGGCCCACCCTCGTGACCACCCTGACCTACGGCGTGCAGTGCTTCAGCCGCTACCCCGACCACATGAAGCAGCACGACTTCTTCAAGTCCGCCATGCCCGAAGGCTACGTCCAGGAGCGCACCATCTTCTTCAAGGACGACGGCAACTACAAGACCCGCGCCGAGGTGAAGTTCGAGGGCGACACCCTGGTGAACCGCATCGAGCTGAAGGGCATCGACTTCAAGGAGGACGGCAACATCCTGGGGCACAAGCTGGAGTACAACTACAACAGCCACAACGTCTATATCATGGCCGACAAGCAGAAGAACGGCATCAAGGTGAACTTCAAGATCCGCCACAACATCGAGGACGGCAGCGTGCAGCTCGCCGACCACTACCAGCAGAACACCCCCATCGGCGACGGCCCCGTGCTGCTGCCCGACAACCACTACCTGAGCACCCAGTCCGCCCTGAGCAAAGACCCCAACGAGAAGCGCGATCACATGGTCCTGCTGGAGTTCGTGACCGCCGCCGGGATCACTCTCGGCATGGACGAGCTGTACAAGTAA

>GFP protein sequence (240aa)

MVSKGEELFTGVVPILVELDGDVNGHKFSVSGEGEGDATYGKLTLKFICTTGKLPVPWPTLVTTLTYGVQCFSRYPDHMKQHDFFKSAMPEGYVQERTIFFKDDGNYKTRAEVKFEGDTLVNRIELKGIDFKEDGNILGHKLEYNYNSHNVYIMADKQKNGIKVNFKIRHNIEDGSVQLADHYQQNTPIGDGPVLLPDNHYLSTQSALSKDPNEKRDHMVLLEFVTAAGITLGMDELYK*
